# A Dirichlet-multinomial mixed model for determining differential abundance of mutational signatures

**DOI:** 10.1186/s12859-025-06055-x

**Published:** 2025-02-18

**Authors:** Lena Morrill Gavarró, Dominique-Laurent Couturier, Florian Markowetz

**Affiliations:** 1 Cancer Research UK Cambridge Institute, University of Cambridge, Cambridge, UK; 2 MRC Weatherall Institute of Molecular Medicine, University of Oxford, Oxford, UK; 3 Human Oncology & Pathogenesis Program, Memorial Sloan Kettering Cancer Center, New York, USA; 4 Medical Research Council Biostatistics Unit, University of Cambridge, Cambridge, UK

**Keywords:** Compositional data, Mutational signatures, Mutational processes, Differential abundance, Compositional regression, Mixed-effects model, Cancer evolution, Cancer aetiology, Clonal evolution, Cancer genomics

## Abstract

**Background:**

Mutational processes of diverse origin leave their imprints in the genome during tumour evolution. These imprints are called *mutational signatures* and they have been characterised for point mutations, structural variants and copy number changes. Each signature has an *exposure*, or abundance, per sample, which indicates how much a process has contributed to the overall genomic change. Mutational processes are not static, and a better understanding of their dynamics is key to characterise tumour evolution and identify cancer cell vulnerabilities that can be exploited during treatment. However, the structure of the data typically collected in this context makes it difficult to test whether signature exposures differ between conditions or time-points when comparing groups of samples. In general, the data consists of multivariate count mutational data (e.g. signature exposures) with two observations per patient, each reflecting a group.

**Results:**

We propose a mixed-effects Dirichlet-multinomial model: within-patient correlations are taken into account with random effects, possible correlations between signatures by making such random effects multivariate, and a group-specific dispersion parameter can deal with particularities of the groups. Moreover, the model is flexible in its fixed-effects structure, so that the two-group comparison can be generalised to several groups, or to a regression setting. We apply our approach to characterise differences of mutational processes between clonal and subclonal mutations across 23 cancer types of the PCAWG cohort. We find ubiquitous differential abundance of clonal and subclonal signatures across cancer types, and higher dispersion of signatures in the subclonal group, indicating higher variability between patients at subclonal level, possibly due to the presence of different clones with distinct active mutational processes.

**Conclusions:**

Mutational signature analysis is an expanding field and we envision our framework to be used widely to detect global changes in mutational process activity. Our methodology is available in the R package *CompSign* and offers an ample toolkit for the analysis and visualisation of differential abundance of compositional data such as, but not restricted to, mutational signatures.

**Supplementary Information:**

The online version contains supplementary material available at 10.1186/s12859-025-06055-x.

## Author summary

The genome is permanently subject to alterations due to errors in replication, faulty replication machinery, and external mutational processes such as tobacco smoke or UV light. Cancer is a disease of the genome, characterised by an abnormal growth of cells that harbour the same set of “clonal” mutations. In turn, these mutations might transform how cells accrue new “subclonal” mutations or the extent to which they tolerate them. The mutational signature framework lets us extract the information of which mutational processes have been active, and in which intensity, in creating a set of mutations. We extend this framework to statistically test the change in the relative intensity of mutational processes between conditions. In samples of 23 cancer types of the PCAWG project, we test the difference between mutational processes that contribute to mutations prior to cancer onset (clonal group), and upon cancer onset (subclonal group), whilst keeping into consideration patient-to-patient differences. We find differences in the majority of cancer types, and identify mutational processes which contribute preferentially to either group.

## Introduction

**Fig. 1:**
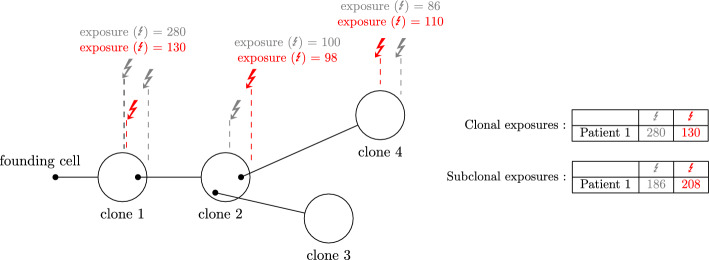
Example of a clonal tree. Four clones are drawn by big circles. A smaller black circle indicates the founder cell of the subsequent clone. The left-most dark circle is the MRCA cell of the tumour, which undergoes clonal expansion to create the first clone, which is defined by clonal mutations of a cancer cell fraction (CCF$=1$). Subsequent clones are defined by subclonal mutations of CCF$<1$. The mutations that define each clone might be of diverse origin: here we depict two active mutational processes (grey and red arrows) creating mutations at two distinct rates. Whilst the mutational process represented by grey arrows is more active at early stages of tumour development, in creating the mutations that define the first clone, the mutational process presented by red arrows has a constant mutation rate. We can quantify the number of mutations created by each mutational processes by extracting signature exposures, which are indicated for each of the clones. These signature exposures can be finally aggregated to clonal exposures (those of clone 1) and subclonal exposures (in this case, those of clone 2 and clone 4). Clone 3 might be interpreted as a clone which is not represented in the sample that has been taken, and for which we therefore not have mutation information

Cancer is a disease of the genome, which is permanently subject to alterations due to external factors, to inevitable errors in replication, and to faulty replication machinery [1–5]. Cancer cell populations grow by clonal expansions. A typical tumour carries several clones—populations of cells with a common genotype. A mutation shared by all cancer cells in the genome is called a clonal mutation, in opposition to a subclonal mutation, which appears in subsequent clones. Fig. 1 shows a diagram of clonal evolution in a cancer sample.

**Mutational signatures** Computational methods on large-scale, genome-wide, genomic data (commonly whole-genome sequencing, WGS) have enabled the use of *mutational signatures* as a proxy for mutational processes to measure the number of processes active in a tumour and quantify the number of mutations created by each one. Mutational signatures represent the processes of mutation and of any subsequent repair [2, 6–8]. They are particularly well established in the context of point mutation signatures, which are repeatedly re-defined and curated in the COSMIC database [9]. In COSMIC signatures, six types of mutations are considered (C$\rightarrow$A, C$\rightarrow$G, C$\rightarrow$T, T$\rightarrow$A, T$\rightarrow$C, and T$\rightarrow$G), as well as their immediate context (their 5’ and 3’ flanking bases), generally without including information about their strandness. This leads to a classification into 96 ($=6\cdot 4\cdot 4$) trinucleotide substitution categories. Therefore, a COSMIC signature is a vector of probabilities of length 96 which sums up to one and indicates the preferences in creating each type of mutation. In the latest version of these signatures (v3.4, October 2023) there are 86 such signatures: SBS1 to SBS99, with some intermediate labels referring to deprecated signatures. As explained in more detail below, in a sample, each signature has a corresponding *mutational signature exposure*, indicating the number of mutations attributable to the signature in the sample. Mutational signatures and their exposures are extracted assuming that the total number of mutations are a linear combination of signature contributions, and where exposures are the coefficients in this linear combination. We denote the exposure matrix $\textbf{Y}$: these are the key quantities we wish to model.

**A short history of signature analysis** Established in 2012, the field of mutational signatures promised not only to bridge the gap between the observable, and possibly very complex, mutational landscape of a tumour at the time of sequencing and its aetiology, but also to be clinically relevant. In the first instance, patient stratification [10] based on exposures suggested treatment groups. This approach has delivered in the case of homologous recombination deficiency (HRD), where mutational signatures are used to determine suitability of PARPi treatment [11]. The use of signatures has, moreover, enabled to discern the nature of other mutational processes, notably APOBEC [12, 13]. However, the number of mutational signatures has been ever-increasing—at least partially due to the increased number of samples used for signature derivation—but few have an aetiology unequivocally linked to them, hampering the interpretation of results and even making the analyses difficult from a statistical point of view. Finally, several variations on single-base substitution signatures have been proposed and adopted: doublet-base substitution signatures [14], indel signatures [14] and copy number signatures [15, 16]. [17] list several statistical challenges in the field, including the difficult choice between alternative methods of signature extraction (each with different assumptions; see Sect. S1) and the inclusion of uncertainty quantification around exposures, which warrants new statistical methods. Even with simpler signature extraction methods, downstream analyses of these data—such as the differential abundance analyses in which we focus—can be complex.

**Motivating example**

We wish to detect differential abundance between clonal and subclonal mutations in the PCAWG cohort, in a histopathological cancer type-specific analysis of 23 cancer types of 27 samples or more. Signatures exposures are extracted using quadratic programming as shown in [18]. The comparison to values obtained by considering mutSigExtractor [19], as well as to values reported in [20] and extracted using sigProfiler and without splitting the mutations into two groups, yield comparable results (Fig. S1). Signatures are extracted using the subset of COSMIC signatures that are considered to be active in each cancer type, taken from [14]. Mutations are classified as clonal and subclonal following [21], considering as clonal mutations which appear in all cells of the tumour, and as subclonal mutations only appearing in a fraction of cells. Details on the assignment of mutations to these categories are beyond the scope of this paper, as this is a step that requires inference of the clonal population structure of the model, is done in a per-sample basis and requires careful consideration—in [21] 11 clonal structure identification methods are used, from which a consensus is derived.

**Signature exposures as compositional data**

In the case under consideration—clonal and subclonal mutations—as well as in most comparisons of mutational signature exposures, the total number of mutations in each observation (i.e. in each patient and group) is not of interest; instead, the crucial information is the relative allocation of these signatures into categories (where categories are mutational signatures, or some other type of mutation categorisation). This renders their analysis suitable in a compositional setting. [22] noted that compositional data can be encoded, without loss of information, using log-ratios. Compositional data in the form of probabilities can be transformed to log-ratios and back using the additive log-ratio transformation (${{\,\textrm{ALR}\,}}$) and inverse additive log-ratio transformation (${{\,\textrm{ALR}\,}}^{-1}$) respectively. In short, compositional data are relative data which must be analysed in relative terms, an important consideration that has usually been overlooked in the mutational signature literature (with few exceptions: [23, 24], only the latter mentioning compositionality), as well as in several other scientific disciplines where compositional data are frequent. Not doing so can lead to spurious correlations and negative biases. Section S2 gives an introduction to compositional data and expands on what makes the data under consideration compositional.

**Precedents in determining differential abundance of signatures** Two papers to date—HiLDA [23] and TCSM [24]—have shown statistical analyses specific to the comparison of exposures in two groups. TCSM tests for differential abundance between two groups of samples following signature extraction and by simulation through the logistic-normal distribution. In HiLDA, the Dirichlet-multinomial is used to model signatures in an MCMC based inference framework. Neither are suitable for a post-hoc analyses of exposures (e.g. using COSMIC signatures) nor do they include random effects. The aim of HiLDA, TCSM and the models proposed here aim to detect changes at a cohort level, while other methods focus on individual samples. Notably, TrackSig [25] is a single-sample method that detects changepoints throughout tumour development, and PhySigs [26] between clones. On the other hand, as compositional data arise in multiple situations, models that analyse equivalent data in other disciplines are more widespread. Recently, [27] implemented a Dirichlet-multinomial model with mixed effects, of which the model described here can be seen as an intension. Table 1 proposes a list of the most relevant existing methods for studying the dynamics of mutational signatures, which is extended in Table S1 with other methods of detecting differential abundance in compositional data.

## Methods

In this paper, we propose an estimator for the Dirichlet-multinomial mixed effect model with multivariate random effects as well as a group-specific precision parameter. The random effects have a multivariate and unconstrained structure to be able to model within-patient correlations as well as correlations between the categories (mutational signatures). The model is used to detect differences in mutational signatures between groups of observations, which are captured in the fixed effects. We opted for the Laplace analytical approximation (LA) to evaluate the high dimensional integrals induced by the random effect structure (Sect. S3). Speed is one of the attractive features of the Laplace approximation compared to alternatives [28]. We present the R package *CompSign*, in which this model and variations of which that have been implemented are made available. With comparable data to that presented, most can be run on a laptop in a few seconds.

The remaining of this paper is organised as follows: the Methods section presents our model and its implementation with Laplace approximation. The first part of the results section describes the operating characteristics of our estimator by Monte Carlo simulations, and compares the results to those of the existing methods HiLDA and TCSM. The second part of the results section is dedicated to the application of our model to the PCAWG dataset, first by using the simple categorisation of mutations into six nucleotide substitutions, and then by using mutational signatures as categories. Finally, we present a discussion on the biological implications of the results, we include two smaller additional use cases where the R package *CompSign* is used, and we provide some guidance on each step of the pipeline of *CompSign*.

### Mixed effects Dirichlet-multinomial regression

**Data** Our goal is to compare signature exposures between two sets of samples. The notation used throughout the paper is found in Table 2. For simplicity we assume that we have paired observations for $N_s$ patients and thus $N=2N_s$ observations in total, although our method is general and can also be applied to un-paired cohorts of different sizes, or to cohorts containing multiple samples per patient. As for each patient a single biological sample is taken, in this document we use the word “sample” to denote the biological sample, and it is therefore used interchangeably with “patient”. In turn, each sample is split into two subsamples, which populate the first (clonal) and second (subclonal) groups. Subsamples are called “observations” in the rest of the manuscript for simplicity. These observations correspond to the response in the model. For the data under consideration, clonal and subclonal labels were assigned after the laborious subclonal inference analysis of the PCAWG study, involving several subclonal inference tools [20], but this step is left at the user’s discretion. Let *K* denote the number of signatures or categories, *i* index patients (or any other group defined by the mixed effects), *k* index either the first $K-1$ signatures or $K-1$ signature log-ratios, and *j* index observations. Let *P* be the number of covariates. Comparing two groups and including a shared intercept leads to $P=2$.

Our data are as follows. We have two exposure matrices of counts corresponding to the clonality-based groups (clonal and subclonal). Each element in each of the two matrices corresponds to the same patient and the same category or signature. Let’s denote these matrices by $\textbf{Y}^{(1)}\in \mathbb {N}^{N_s\times K}$ and $\textbf{Y}^{(2)}\in \mathbb {N}^{N_s\times K}$ where (1) and (2) refer to clonal and subclonal groups. In the case under consideration, for each sample and clonality-based group (i.e., for each observation), mutations are classified according to their trinucleotide context, and signature exposures are estimated independently. We row-concatenate the clonal and subclonal matrices to create $\textbf{Y}\in \mathbb {N}^{N \times K}$ where $N=2N_s$.

**Fixed-effects** Firstly, we express the log-ratios of signature abundances as a function of the fixed effects. The fixed effects are captured by the $\textbf{X}\in \mathbb {R}^{N\times P}$ design matrix, and their corresponding coefficients are a $\varvec{\beta }\in \mathbb {R}^{P\times (K-1)}$ parameter matrix. Unless specified otherwise, in the applications shown $\textbf{X}$ is a binary matrix of ones in the first column, and zeros or ones in the second (with a zero if the sample belongs to the clonal group, and a one otherwise). In practice, the implementation we propose does not have this constraint: $\textbf{X}$ can take any value in $\mathbb {R}$ and *P* can be greater than 2, as will be shown.

The first row of $\varvec{\beta }$, $\varvec{\beta }_0$, is a vector of length $K-1$ which corresponds to the general abundance of signatures in the reference mutational group (clonal group), expressed as the ALR transformation using the last signature as reference, i.e. as the log-ratio of a signature to a baseline signature. Let $M_i^{(1)}\in \{1,\ldots ,K\}^{T_i^{(1)}}$ be the sequence of mutations for patient *i* in the clonal group, and $M_i^{(2)}\in \{1,\ldots ,K\}^{T_i^{(2)}}$ the equivalent for the subclonal group. Let these mutations be indexed by *l*. Thus, for $k<K$ and a case without patient-dependence (i.e. no random effect), we have

1 $$\begin{aligned} \varvec{\beta }_{{0}_{k}} = \ln \frac{{{\,\textrm{Pr}\,}}\left( m_{il}^{(1)} = k\right) }{{{\,\textrm{Pr}\,}}\left( m_{il}^{(1)} = K\right) }, \end{aligned}$$

where $\varvec{\beta }_{0_{k}}$ denotes the *k*th element of $\varvec{\beta }_{0}$, and ${{\,\textrm{Pr}\,}}(m_{il}^{(1)} = k)$ denotes the probability that the *l*th mutation of patient *i* in the reference group is generated by signature *k*. For $k<K$, this probability is then given by

2 $$\begin{aligned} {{\,\textrm{Pr}\,}}\left( m_{il}^{(1)} = k\right) = {{\,\textrm{Pr}\,}}\left( m_{il}^{(1)} = K\right) e^{\varvec{\beta }_{{0}_{k}}} = \frac{e^{\varvec{\beta }_{{0}_{k}}}}{1+\sum _{k=1}^{K-1}e^{\varvec{\beta }_{{0}_{k}}}}. \end{aligned}$$

The second row of $\varvec{\beta }$, $\varvec{\beta }_1$, is our main parameter of interest, as it indicates whether there are shifts in the general abundance of signatures for samples of the subclonal group compared to the clonal reference, in ALR space. Similarly to above, $\varvec{\beta }_{{0}_{k}}+\varvec{\beta }_{{1}_{k}}$ is the log-ratio of the probability that the *l*th count of patient *i* in the second group is generated by signature *k*, over the probability that it is generated by signature *K*, is

3 $$\begin{aligned} \varvec{\beta }_{{0}_{k}}+\varvec{\beta }_{{1}_{k}} = \ln \frac{{{\,\textrm{Pr}\,}}\left( m_{il}^{(2)} = k\right) }{{{\,\textrm{Pr}\,}}\left( m_{il}^{(2)} = K\right) }. \end{aligned}$$

$\varvec{\beta }_{{1}_{k}}$ equal to zero indicates that the *k*th log-ratio is the same in both groups, and that therefore the relative mutation rates of the signatures of both groups are equal. A zero vector of $\varvec{\beta }_1$ corresponds to cases without differential abundance between groups. A test for overall differential abundance can be computed by using the generalised Wald statistic *w* in a $\chi ^2$ test with $K-1$ degrees of freedom, combining all the estimates of $\varvec{\beta }_1$ and their correlations:

4 $$\begin{aligned} \begin{aligned} w = \widehat{\varvec{\beta }}_1^{\top } \Sigma _{\widehat{\varvec{\beta }}_1}\widehat{\varvec{\beta }}_1,\\ w \sim \chi ^2_{K-1} \end{aligned} \end{aligned}$$

where $\widehat{\varvec{\beta }}_1$ and $\mathbf {\Sigma }_{\widehat{\varvec{\beta }}_1}$ respectively denote the estimated $\varvec{\beta }_1$ parameter vector and its corresponding covariance matrix.

**Random intercepts** Secondly, the log-ratios of abundances of signatures also depend on the random effects, in which we include information about the patient from whom each observation derives. In the PCAWG dataset, having one observation per patient and group, i.e. two observations per patient, we use the matrix $\textbf{Z}\in \{0,1\}^{2N_s \times N_s}$ (more generally, $\textbf{Z}\in \{0,1\}^{N \times N_s}$). The coefficients for $\textbf{Z}$ are encapsulated in the matrix $\textbf{U}\in \mathbb {R}^{N_s\times (K-1)}$, where each row of $\textbf{U}$ corresponds to the patient-specific multivariate intercepts and where the values are, too, ALR-transformed with respect to the baseline signature. These intercepts are drawn from a multivariate normal distribution of mean zero (as the overall abundance of signatures in the first group is already captured by $\varvec{\beta }_0$), and a covariance matrix $\mathbf {\Sigma }$ which can be unconstrained or not. These multivariate random intercepts differentiate our implementation from the one of [27] and allow us not only to model the potential within-patient dependence with more flexibility but also to have positive correlations between signatures.

**Logit link** The linear combination of fixed and random effects is linked to the log-ratios of abundances as follows, in the commonly used logit link:

5 $$\begin{aligned} \bar{\varvec{\alpha }}^{{{\,\textrm{ALR}\,}}} = \textbf{X}\varvec{\beta }+ \textbf{Z}\textbf{U} \end{aligned}$$

where $\bar{\varvec{\alpha }}^{{{\,\textrm{ALR}\,}}}\in \mathbb {R}^{N\times (K-1)}$ are row-wise ALR-transformed quantities. Therefore, each row can be transformed into a vector of probabilities $\bar{\varvec{\alpha }}_j$ using the inverse-ALR transformation ${{\,\textrm{ALR}\,}}^{-1}$, or generalised softmax transformation:

6 $$\bar{\alpha }_{{jk}} = \begin{array}{*{20}l} {\frac{{e^{{\bar{\alpha }_{{jk}}^{{{\mkern 1mu} {\mkern 1mu} {\text{ALR}}{\mkern 1mu} {\mkern 1mu} }} }} }}{{1 + \sum\limits_{{k = 1}}^{{K - 1}} {e^{{\bar{\alpha }_{{jk}}^{{{\mkern 1mu} {\mkern 1mu} {\text{ALR}}{\mkern 1mu} {\mkern 1mu} }} }} } }}} \hfill & {(k = K),} \hfill \\ {\frac{1}{{1 + \sum\limits_{{k = 1}}^{{K - 1}} {e^{{\bar{\alpha }_{{jk}}^{{{\mkern 1mu} {\mkern 1mu} {\text{ALR}}{\mkern 1mu} {\mkern 1mu} }} }} } }}} \hfill & {(k = K),} \hfill \\ \end{array}$$

where $\bar{\varvec{\alpha }}_{jk}$ represents the probability of counts of the *j*th observation to have been generated from the *k*th signature.

**Dirichlet-multinomial** The vector $\bar{\varvec{\alpha }}_{j}$ corresponds to the mean of the Dirichlet-multinomial. If a multinomial distribution were to be used instead, this would be the its sole parameter. However, in using the Dirichlet-multinomial, we introduce the precision parameter vector $\varvec{\lambda }$, such that, for the *j*th observation, the Dirichlet-multinomial parameter $\varvec{\alpha }_j$ is defined as product of the probability described above and the scalar $\lambda _j = \textbf{d}^{\top }_j\varvec{\lambda }$, where $\textbf{d}_j$ denotes the *j*th row of the ($N \times Q$) $\textbf{D}$ precision predictor matrix, so that

7 $$\begin{aligned} \varvec{\alpha }_j = \lambda _j \bar{\varvec{\alpha }}_j \end{aligned}$$

Without loss of generality, we model the precision as a function of the groups only, so that $\textbf{D}=\textbf{X}$ and the two values of the $\varvec{\lambda }$ vector correspond to the precision level in the reference group and to the shift in precision between both groups, respectively. Higher values of $\varvec{\lambda }$ lead to more concentrated results, or lower overdispersion. In practice, we estimate $\log (\varvec{\lambda })$, to ensure positivity. Lastly, the counts of the *j*th observation are drawn from a Dirichlet-multinomial (DM) distribution as follows

8 $$\begin{aligned} \textbf{y}_j \sim \text {DM}\left( \varvec{\alpha }_j, T_j\right) \end{aligned}$$

where $T_j=\sum _{k'=1}^{K} {y}_{jk'}$ corresponds to the total number of mutations observed for observation *j*.

**Model summary** Therefore, our mixed-effect Dirichlet-multinomial model can be summarised by the following equations:

9 $$\begin{aligned} \begin{aligned} \textbf{u}_i\sim {{\,\textrm{MVN}\,}}(\textbf{0}_{K-1}, \mathbf {\Sigma }),\\ \bar{\varvec{\alpha }}_j = {{\,\textrm{ALR}\,}}^{-1}\left( \textbf{x}_j\varvec{\beta }+ \textbf{z}_j\textbf{U}\right) ,\\ \textbf{y}_j\sim \text {DM}\left( \lambda _j \bar{\varvec{\alpha }}_j, T_j\right) , \end{aligned} \end{aligned}$$

**Fig. 2:**
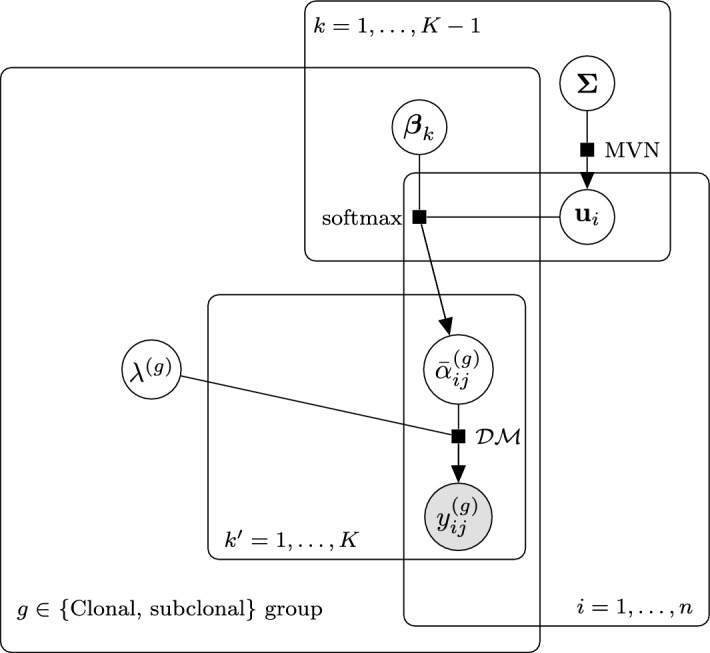
Plate diagram for the Dirichlet-multinomial model. The random intercepts $\textbf{u}_i$ are independent of clonality-based grouping, and in log-ratio space, as are the coefficients $\varvec{\beta }$. The precision parameters $\varvec{\lambda }$ are a function of the clonality-based grouping exclusively in this manuscript, but adjustable in their implementation. Similarly, $\textbf{X}$ and $\textbf{D}$ (the covariate matrices for the fixed effects and precision) are shared here and reflect the clonality group *g*, but that need not be the case and they are specified independently in the model implementation

The dependencies between parameters and elements of the data are displayed in the plate diagram proposed in Fig. 2.

### Availability of models in the R package *CompSign*

**Implementation in Template Model Builder** All models considered in this paper are implemented using Template Model Builder (TMB) [29], a framework for maximum likelihood estimation in random effects models, in which the random effects are integrated out of the likelihood using the Laplace approximation. TMB has an R interface, and the model is written in C++. The mixed effects models presented in this paper have been assembled into the R package *CompSign* (https://github.com/lm687/CompSign), which includes vignettes with example data and instructions on how to run the functions and interpret the results.

**Variants of the model** In the main part of this paper, where the data comprise two observations per patient and two groups, we compare several of these variations of the mixed-effects Dirichlet-multinomial model:

- diagREDM is a DM model that considers signature-dependent random-effects (independently for each signature log-ratio) and group-dependent precision parameters $\varvec{\lambda }$,
- fullREDM is a DM model that considers signature-dependent (and possibly correlated) random-effects and group-dependent precision parameters $\varvec{\lambda }$,
- singleREDM is a DM model that considers signature-independent random-effects (a univariate random intercept drawn from one single $\sigma$) and group-dependent precision parameters $\varvec{\lambda }$.

Additionally, the following models are used with two additional use cases where the data no longer comprise two observations per patient and two groups but, (a) several groups of unmatched data, or (b) a regression setting:

- fullREM is a multinomial model that considers signature-dependent (and possibly correlated) random-effects and group-dependent precision parameters $\varvec{\lambda }$,
- FEDMsinglelambda is a DM model that considers no random effects (only fixed-effects) and a shared precision parameter $\lambda$,
- diagREDMpatientlambda is a DM model that considers signature-dependent random effects (independently for each signature log-ratio) and patient-dependent precision parameters $\varvec{\lambda }$.

Table S2 contains the comprehensive list of models implemented in *CompSign* with indications of when their usage might be appropriate.

**Model assumptions** We assume in all cases that the mutational processes active in the clonal group are also active in the subclonal group, possibly in different relative activity. As with most compositional models, scenarios where all exposures are zero in a group and non-zero in the other are problematic (Sect. S2.1), but this is not a scenario seen in any of PCAWG datasets to which the models have been applied, even if zeros are common (Table S3). The exposures in the subclonal group might be representative of only a subset of the clones: as $\varvec{\beta }_1$ represents a cohort-wide average, a large increase in the mutation rate of a process active in a single clone can result in a moderate $\varvec{\beta }_1$ provided the clone represents a small fraction of cells in the tumour. diagREDM assumes that mutational processes are not correlated across patients, although the multivariate random effects paired with the group-dependent precision parameter $\varvec{\lambda }$ allows for correlations to some extent. fullREDM, in that it does allow for such correlations or co-occurrences by estimating the covariance matrix of signature abundances, may suffer from convergence problems if some signature is present in very low abundance and the dataset includes few samples.

## Results

### Simulation-based assessment of model performance

**Bias and coverage of estimator** The adequacy of the model can be assessed by means of simulations. Simulated datasets A1-3 represent two-group and patient-matched signature exposures in a cancer type cohort. The set of active signatures is shared in all the observations of each simulated dataset. The bias and coverage of 95% confidence intervals for elements of $\widehat{\varvec{\beta }}_1$, the parameter vector of interest, as well as for elements of $\hat{\varvec{\beta }_0}$, are assessed by means of Monte Carlo simulations considering 1000 simulated datasets of n=200 samples – a sample size in line with the number of patients often considered in such studies. Although the recovery of parameters $\varvec{\beta }_0$ and $\varvec{\beta }_1$ can suffer with increasingly lower values of $\lambda$ (higher dispersion) (Figs. S2, S3), the recovery of $\varvec{\beta }$ is mostly satisfactory. When datasets have been created with independence between the random effects, diagREDM and fullREDM lead to equivalent results (Figs. S5, S6). When correlated random effects are used, this can lead to biases in the estimation of elements of $\varvec{\beta }_0$ for diagREDM (but not for fullREDM), but this does not translate to biases or lower coverages in the elements of $\varvec{\beta }_1$, our parameter of interest, provided multivariate random effects are used (Figs. S4, S6). Further simulations (B1-4, Sect. S4.1) are generated to match biologically-relevant parameters. Both bias and coverage results are satisfactory in all biologically-inspired datasets (Figs. S5S6, S6, S7, S9). In these simulations the number of mutations is either representative of the cancer type from which representative parameters were chosen, or lower than typical number of observed mutations (Fig. S8). Indeed, we find that the results of the models are robust to lowering the number of mutations up to 10-fold (Fig. S9). Due to the strong reduction in the number of parameters to estimate and their agreement in $\widehat{\varvec{\beta }}_1$, diagREDM thus appears as very attractive compared to fullREDM. Finally, to support the validity of findings from the PCAWG analyses shown in the results section, bias and coverage have been assessed for datasets generated with the parameters estimated by diagREDM, with satisfactory results in all cases (Fig. S10).

**Comparison with previous models of differential abundance** The output of HiLDA, TCSM and diagREDM is compared in a simulation setting. To avoid simulating from the model, and because both TCSM and HiLDA take as input substitution categories (and not pre-computed exposures), we use a new approach to generate the datasets in simulations C1-C3 (Fig. S11). In a simulation inspired by [30], biologically-informed exposures are generated by mixing, in some mixing proportion $\pi$, mutations generated by two sets of COSMIC exposures. The scenario of no differential abundance corresponds to setting $\pi$ to zero, where exposures in both groups come from the same distributions. With increasingly high values of $\pi$, differential abundance becomes more apparent. From these simulated exposures, mutations categorised into 96 trinucleotide substitutions are generated based on COSMIC signature definitions, and are used as input for HiLDA and TCSM. For diagREDM, exposures are re-computed from the trinucleotide substitutions using quadratic programming, and they become the input for the model. The two sets of exposures used for mixing differ between the three simulation strategies C1-3 (Sect. S4.5.1).

**Fig. 3:**
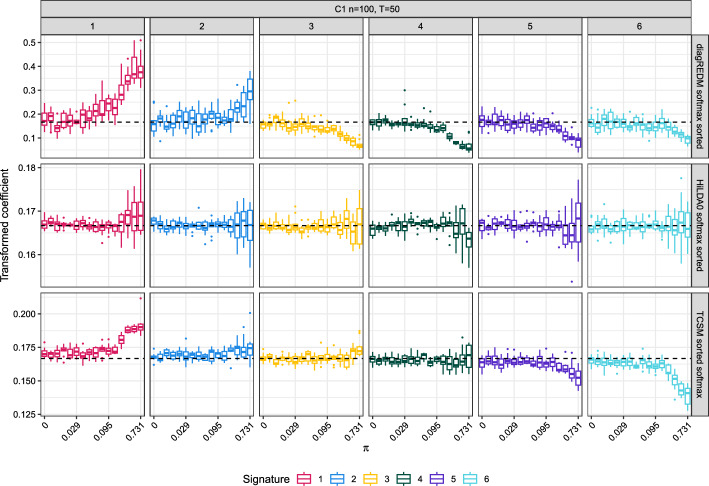
Comparison of coefficients that represent differential abundance for the models under consideration: diagREDM, HiLDA, and TCSM, for simulated dataset C1. Although diagREDM shows a marked increase in dispersion of softmax-transformed $\widehat{\varvec{\beta }}_1$ across signatures (indicating differential abundance) and the coefficients of TCSM show an increased deviation from zero (also indicating differential abundance), the results from HiLDA do not show such trends unless the mixing proportion $\pi$ is very high. In the case of diagREDM, the signatures represent the ground truth signatures used for simulation (SBS1, SBS5, SBS9, SBS40, SBS3, SBS13)

The results on differential abundance from HiLDA, TCSM and diagREDM are markedly different, across simulation strategies and as parameters vary. Whereas HiLDA never finds any differential abundance (indicating a high false negative rate), TCSM almost always does (indicating a high false negative rate) (Table S4). diagREDM is the only model with progressively higher true positive rates, as expected, as $\pi$ increases (Fig. S12). We additionaly compare the recovery of ground-truth values by analysing the estimated values of (1) $\varvec{\beta }_1$ (or equivalent coefficients for differential abundance), (2) $\varvec{\beta }_0$, (3) signature definitions, and (4) signature exposures. Regarding (1) the coefficients that indicate differential abundance, as they differ in each model and have to be transformed (Sect. S4.5.2). Fig. 3 shows the value of these coefficients as $\pi$ increases, for C1 (and Fig. S13, S14 for C2, C3). Values that differ from the dashed line indicate differential abundance. For C1, the changes are apparent in diagREDM and TCSM even when datasets are mixed in a 92-to-8% proportion, whereas in HiLDA a 50-50% mixture is necessary. In comparing estimated $\varvec{\beta }_1$ to the ground-truth $\varvec{\beta }_1$, only diagREDM performs well (Fig. S15). In C1, the data have been generated by mixing exposures from two cancer types with an only partial overlap of active signatures. Therefore, regarding (2) $\varvec{\beta }_0$, at high values of $\pi$, we expect to find some very low values of $\widehat{\varvec{\beta }}_0$ that correspond to practically inactive signatures. This expected relative sparsity of signature exposures in datasets of high $\pi$ is clearest in the results from diagREDM (Fig. S16, Sect. S4.5.3). Regarding (3) signature definitions, only in the case of TCSM can the estimated signature definitions be compared to the ground truth—in diagREDM the ground truth signatures are used to re-extract exposures, and HiLDA does not use the 96 trinucleotide substitution categorisation. TCSM often re-extracts signatures similar to those used in the simulation (Fig. S17). Finally, regarding (4), exposures can be compared in all three cases. Keeping in mind that they are only estimated *de novo* in TCSM and HiLDA, the re-extracted exposures from quadratic programming are more representative of the ground truth exposures than TCSM and HiLDA exposures (Fig. S18).

The parameters used for simulation influence the success in parameter recovery. In diagREDM an increase in the number of mutations or the number of samples leads to higher statistical power (Fig. S12), in TCSM it leads to a better recovery of signature definitions (Fig. S17). When a low number of mutations is included, exposures are better recovered in HiLDA than in TCSM, and they are perfectly recovered by quadratic programing (for diagREDM) when the number of mutations is high (Fig. S18). In terms of runtime, diagREDM is consistently the fastest method out of the three, often by several orders of magnitude, and partially owing to the fact that signature extraction has been performed beforehand (Sect. S4.5.6).

**Effect of active signature selection on differential abundance** Unlike in HiLDA and TCSM, where a joint *de novo* signature extraction and differential abundance test is performed and the number of active signatures is a given parameter, the models suggested here require signature exposures to have been derived from a known set of active signatures prior to parameter estimation. The selection of active signatures is a topic of debate and careful thought is put in choosing the set of active signatures in each cancer type, or even sample. By generating simulated signature exposures using different strategies of active signature selection, we assess the robustness of our differential abundance model diagREDM. We find that, unless the number of active signatures is severely overestimated, leading to a high false positive rate, the differential abundance results are comparable across active signature selection strategies (Fig. S19, Sect. S4.5.7). More results on the robustness of results to changes in the set of active signatures, specific to the PCAWG cohort, will be referred to in the following section.

### Application of the model to PCAWG data

The model diagREDM, and fullREDM where applicable, are applied first to mutation data grouped in simple nucleotide substitutions ({C$\texttt {>}$A, C$\texttt {>}$G, C$\texttt {>}$T, T$\texttt {>}$A, T$\texttt {>}$C, T$\texttt {>}$G}) and secondly to mutational signature exposures. The latter provides a better platform to identify the mutational processes behind a change in mutation type abundance. In the context of mutational signatures, the estimates of coefficients representing differential abundance ($\varvec{\beta }_1$), differential precision ($\varvec{\lambda }$) and patient heterogenity ($\textbf{U}$) are analysed. Finally, $\varvec{\beta }_1$ are analysed more in depth to point to specific mutational processes the activity of which changes over time during tumour development. The results of this section are robust to numerous perturbations to the data: to the addition or removal of signatures (Fig. S20), even if those are signatures with *flat* definitions (Fig. S21), to the inclusion of signatures which are a linear combination of each other (Figs. S22, S23), to a reduction in the number of mutations (Figs. S24, S25) or samples (Figs. S26, S27), and to some misclassification of mutations in the clonal and subclonal groups (Fig. S28, S29). Notably, more than 60% of samples or mutations can be removed with practically undistinguishable $\varvec{\beta }_1$ values in most cancer types.

**Differential abundance of nucleotide changes**

**Fig. 4:**
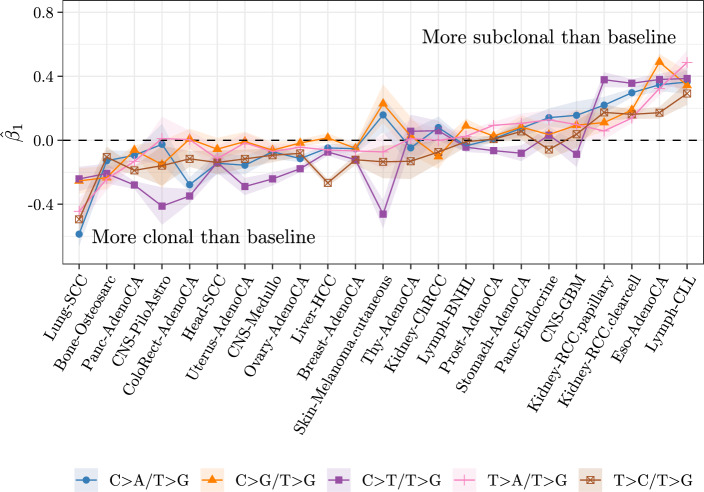
$\widehat{\varvec{\beta }}_1$ estimated using fullREDM on single-nucleotide mutations in 23 cancer types of the PCAWG cohort, using the mutation category T$\texttt {>}$G as baseline. In four cases where fullREDM did not converge (CNS-Medullo, CNS-PiloAstro, Lymph-CLL, Thy-AdenoCA), diagREDM is used instead. The ribbons indicate the estimate ± its standard error

The Dirichlet-multinomial fullREDM model has been fit on a per-cancer type basis on data categorised into six different types of mutation ({C$\texttt {>}$A, C$\texttt {>}$G, C$\texttt {>}$T, T$\texttt {>}$A, T$\texttt {>}$C, T$\texttt {>}$G}) to compare clonal and subclonal mutations. In four cases where the model has not converged due to a comparatively lower number of samples, diagREDM is used instead. T$\texttt {>}$G is used as baseline. Importantly, the choice of baseline does not impact the results of these models beyond the interpretability of coefficients (Fig. S30). The results of the model indicate that the type of mutations created at clonal and subclonal differ, and that the way in which they differ is particular to each cancer type. C$\texttt {>}$T constitutes the most abundant mutation type in most cancer types (19/23), whilst T$\texttt {>}$G is the least abundant in 15/23. Coherently, $\widehat{\varvec{\beta }}_0$ corresponding to C$\texttt {>}$T with respect to T$\texttt {>}$G are the highest coefficients, and they are positive, in 20/23 cancer types, indicating that they constitute the most abundant mutation type in clonal stages. Fig. 4 shows $\widehat{\varvec{\beta }}_1$, the estimate and confidence interval of $\varvec{\beta }_1$, for each cancer type, where each element of $\widehat{\varvec{\beta }}_1$ corresponds to the log-ratio of a mutation type $\widehat{\varvec{\beta }}_1$ corresponding to the same log-ratio are colour-coded and joined by a line, to enable the identification of similar trends of differential abundance across cancer types. The estimates vary greatly across cancer types. Generally, either all $\varvec{\beta }_1$ are negative, indicating that the baseline mutation type, T$\texttt {>}$G, is more subclonal than the rest of mutations, or they are all positive, indicating that it is more clonal. C$\texttt {>}$T often has the lowest coefficient and it is negative. Overall, this makes C$\texttt {>}$T and T$\texttt {>}$G the most clonal mutation types globally. Pan-cancer, the $\widehat{\varvec{\beta }}_1$ coefficients for C$\texttt {>}$T (with respect to baseline category T$\texttt {>}$G) are generally not correlated with other coefficients across cancer types (highest Pearson correlation coefficient of 0.7, in correlation with T$\texttt {>}$A), whereas those of T$\texttt {>}$C and T$\texttt {>}$A are highly correlated (i.e. their lines follow each other, often sharing the same $\widehat{\varvec{\beta }}_1$; $cor=0.9$ *p*-value = $5\cdot 10^{-9}$, Pearson’s product-moment correlation) and their confidence intervals overlap in 21/23 cases. Additionally, the confidence intervals of C$\texttt {>}$A/T$\texttt {>}$G, C$\texttt {>}$G/T$\texttt {>}$G, T$\texttt {>}$A/T$\texttt {>}$G and T$\texttt {>}$C/T$\texttt {>}$G often overlap, in that at least 20 cancer types show an overlap in any pair of these coefficients, while C$\texttt {>}$T/T$\texttt {>}$G behaves more independently, in that the confidence intervals of their $\widehat{\varvec{\beta }}_1$ do not overlap with the others as commonly. On the other hand, the value of $\varvec{\beta }_1=0$, corresponding to the baseline category T$\texttt {>}$G, is mostly not included in these confidence intervals either (only in 47% of $\widehat{\varvec{\beta }}_1$, across cancer types). Therefore, we find that T$\texttt {>}$A, T$\texttt {>}$C, C$\texttt {>}$A and C$\texttt {>}$G have comparatively similar patterns of differential abundance across cancer types, while C$\texttt {>}$T and T$\texttt {>}$G vary widely across cancer types. Part of the explanation for this behaviour is that C$\texttt {>}$T mutations reflect a variety of mutational processes, exogenous (e.g. UV light [31]) or not (e.g. spontaneous deamination of 5-methylcytosine [32]), and so do mutations in T$\texttt {>}$G (exogenous when created by UVA light [33] but not linked to clinical or exposure covariates in other cancer types such as oesophageal adenocarcinoma [34]). The commonalities might be explained by shared underlying mutational processes in only some cases: T$\texttt {>}$A might represent mutations due to activation-induced cytidine deamination [14], and so might C$\texttt {>}$G [35], but the high for $\widehat{\varvec{\beta }}_1$ for C$\texttt {>}$G in melanoma is explained by hightened polymerase iota activity [36]. Overall, these results suggest patterns of differential abundance between clonal and subclonal mutations even at the lowest level of granularity, which prompts us to extend this approach to mutational signatures in order to link them to changes in mutational processes.

**Widespread differential abundance of mutational signatures** In this analysis of PCAWG data, diagREDM has been fit on exposures extracted on the subset of signatures considered to be active in each cancer type, according to [14] (Table S6). All cancer types are differentially abundant when including all signatures, and most (17/23) are when including only non-exogenous signatures (following Benjamini & Hochberg adjustment). The six cancer types that cease to be differentially abundant are CNS-Medullo, CNS-GBM, CNS-PiloAstro, Kidney-ChRCC, Lung-SCC and Stomach-AdenoCA. These six cancer types do not differ in the number of samples (Welch Two Sample t-test; *p*-value = 0.08733), nor in the number of active non-exogenous signatures (Welch Two Sample t-test; *p*-value = 0.1858, with an average of 8.5 signatures in the group of differentially-abundant cancer types and of 5.5 in the group of non-differentially abundant cancer types) nor in the average number of mutations constituting the observed exposures (Welch Two Sample t-test on $\log _2$-transformed mutation toll; *p*-value = 0.425). Rather, it is the number of patient samples which can more strongly limit our ability to determine differential abundance, as discussed and assessed by simulations (Figs. S12, S26), and this value varies notably across cancer types (Table S7). To have a better indication of the general abundance of signatures, or of which signatures are behind these changes, $\widehat{\varvec{\beta }}_0$ and $\widehat{\varvec{\beta }}_1$ should be analysed respectively. These values are plotted in Figs. S31 and S32, but before they are analysed in depth, we discuss the estimates $\widehat{\varvec{\lambda }}$ and $\widehat{\textbf{U}}$.

**Fig. 5:**
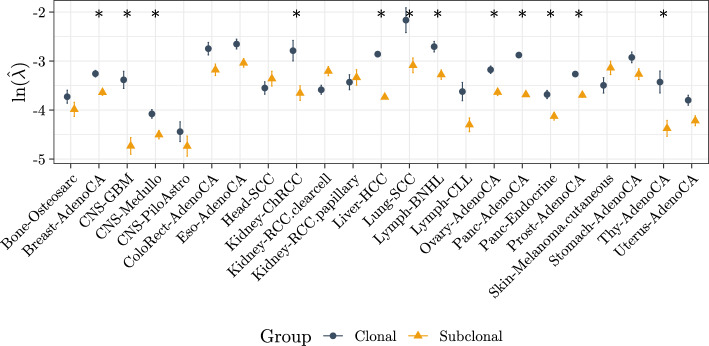
Estimated precision parameters $\varvec{\lambda }$, for each of the groups, in each cancer type. Higher values indicate higher precision, or lower overdispersion. In most cases (19/23) the subclonal mutations show higher overdispersion rates, which are to be expected if subclones have different relative signature exposures. The bars indicate the ± standard error. Asterisks indicate cancer types for which the precision parameters are statistically not the same (t-test, FDR correction)

**Differential precision of mutational signatures** The models include group-specific precision parameters $\varvec{\lambda }$, to account for possible overdispersion in one group. Fig. 5 shows the log-transformed estimated precision parameters $\widehat{\varvec{\lambda }}$, for the clonal and subclonal groups of mutations in each cancer type. Higher values indicate higher precision, or lower overdispersion. In most cases (19/23) the subclonal mutations show higher overdispersion, which is to be expected if subclones have different relative signature exposures, or if patients diverge in the types of active mutational processes following cancer onset. There is a statistically-significant difference in precision in 12/23 cancer types (Breast-AdenoCA, CNS-GBM, CNS-Medullo, Kidney-ChRCC, Liver-HCC, Lung-SCC, Lymph-BNHL, Ovary-AdenoCA, Panc-AdenoCA, Panc-Endocrine, Prost-AdenoCA, Thy-AdenoCA; t-test with FDR adjustment). In all statistically-significant cases the precision is higher in the early mutation group. Head-SCC, Kidney-RCC (clear cell), Kidney-RCC (papillary) and melanoma are the cancer types where there is higher precision in the late mutation group, albeit not significantly. There are multiple additional reasons why dispersion might be higher in subclonal than clonal mutations: the presence of subclones with distinct active mutational processes, the emergence of subclonal signatures that reflect interactions between mutational processes in more advanced stages of cancer, the presence of noise particular to mutations of lower coverages, or a less accurate signature extraction for subclonal mutations due to e.g. the lower number of subclonal than clonal mutations. Through simulations, we have found that differential precision is not merely a reflection of the generally higher number of mutations in the clonal group (Fig. S24).

**Identification of patient strata from their random intercepts**

A third output of the model are the estimated patient random intercepts $\textbf{U}$, which give an indication of patient heterogeneity in clonal exposures. TMB outputs their point estimates at the maximum (Fig. S33), as well as values for the estimated correlation matrix if fullREDM is used (Fig. S34). The correlation between two signatures in contributing to the random effects differs among cancer types, i.e. there is rarely a clear pattern of co-occurrence of signatures (Fig. S35 for selected pairs of signatures of interest). The extent to which patients can be categorised into strata according to their mutational signatures, based on the patient intercepts, also varies between cancer types: in Lymph-CLL at least two clear strata can be defined based on SBS9 (Fig S33), and so is the case in Prost-AdenoCA due to a range of signatures (but especially due to SBS3), whereas the Lung-SCC cohort appears much more uniform. The abundance of HRD signature SBS3 varies greatly among patients of Breast-AdenoCA, Eso-AdenoCA, Ovary-AdenoCA, Panc-AdenoCA and Prost-AdenoCA, driving their patient stratification. In cases where fullREDM converged, we are able to compare the estimated covariance matrices to the empirical covariance matrix computed from the random intercepts. The correlation between these values ranges from a Pearson correlation of 0.47 (in the case of Uterus-AdenoCA) to 0.998 (in Lymph-CLL), with a median correlation of 0.769. Having considered the cohort-wide results from the point of view of differential abundance, differential precision, and stratification based on the estimated random intercepts, we now focus on signature-specific results.

**Fig. 6:**
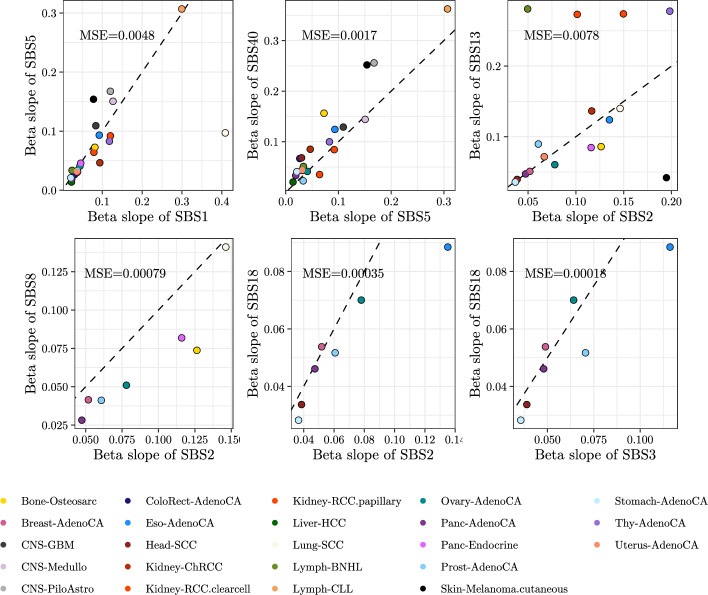
Selected pairs of signatures of interest and with constant ratios of (softmax-transformed) $\widehat{\varvec{\beta }}_1$. Each point represents a cancer type, and a tighter match along the identity line (the dashed line $x=y$) indicates a higher agreement in the differential abundance characteristics, i.e. a constant fold-change in their abundance. Only signatures which are shared between each pair of cancer types are shown in the plot. The mean squared error is shown for each pair of signatures. Note the very good agreement between SBS1 and SBS5 (signatures of proposed constant mutation rate) and the consistently lower values for the softmax-transformed $\widehat{\varvec{\beta }}_1$ of SBS2 compared to SBS8

**Shared patterns of differential abundance at the signature level** The coefficients $\widehat{\varvec{\beta }}_1$ can give us an indication of the differential abundance patterns of mutational processes, which might be shared or not across signatures and cancer types. This is of particular interest in the signatures of unknown aetiology (u.a. henceforth), as it offers a possibility of associating them with signatures of known aetiology. For this analysis, the vectors $\widehat{\varvec{\beta }}_1$ have been softmax-transformed to be able to draw conclusions on the baseline signatures. These transformed $\widehat{\varvec{\beta }}_1$ can still be used to compare two signatures within cohorts, as the log-ratios of abundance are preserved in the transformation. Importantly, correlations between $\widehat{\varvec{\beta }}_0$ or $\widehat{\varvec{\beta }}_1$ of signatures can reflect spurious and imposed correlations (e.g. a high correlation in signature abundance in softmax-transformed $\widehat{\varvec{\beta }}_0$ between two signatures across cancer types can be a reflection of the large variance in the abundance of a third signature, and the same is the case for $\widehat{\varvec{\beta }}_1$). Fig. 6 displays scatterplots of softmax-transformed $\widehat{\varvec{\beta }}_1$ for pairs of signatures of interest (six pairs which include clock-signatures SBS1, SBS5 and SBS40, APOBEC signatures SBS2 and, SBS13, and two signatures of high abundance and active in multiple cancer types but with unclear aetiology, SBS8 and SBS18). In each plot, a point represents a cancer type. Tighter alignments along the identity line indicate a higher agreement in differential abundance, i.e. in the log-fold change in mutational signatures. Signatures that share the same $\widehat{\varvec{\beta }}_1$ across cancer types, therefore, are hypothesised to share a biological mechanism. Outliers in an otherwise good agreement might indicate particularities of the relevant mutational processes in the specific cancer type.

**Concordance of clock signatures SBS1, SBS5 and SBS40**

Reassuringly, there is generally good agreement in $\widehat{\varvec{\beta }}_1$ of known age-related signatures SBS1 and SBS5 [14, 37]. SBS40, sometimes described as an age signature, and a pervasive signature of high abundance, has $\widehat{\varvec{\beta }}_1$ that match with those of SBS5. We call $\widehat{\beta }_1^{\text {SBS1}}$ the $\varvec{\beta }_1$ coefficient corresponding to the log-ratio which has SBS1 in its numerator, and $\widehat{\beta }_1^{\text {SBS5}}$ the equivalent for SBS5. In the case of SBS1 and SBS5, an outlier (Lung-SCC) can be seen in the first facet of Fig. 6. Particularly, $\widehat{\beta }^{\text {SBS1}}_1$ is much higher than $\widehat{\beta }^{\text {SBS5}}_1$, and SBS1 is found in very low abundance (Fig. S31)—these two pieces of information, together with the fact that in no other cancer type is SBS1 the signature of least abundance, warrant the question of whether SBS1 should be considered inactive in the PCAWG Lung-SCC cohort.

**Fig. 7:**
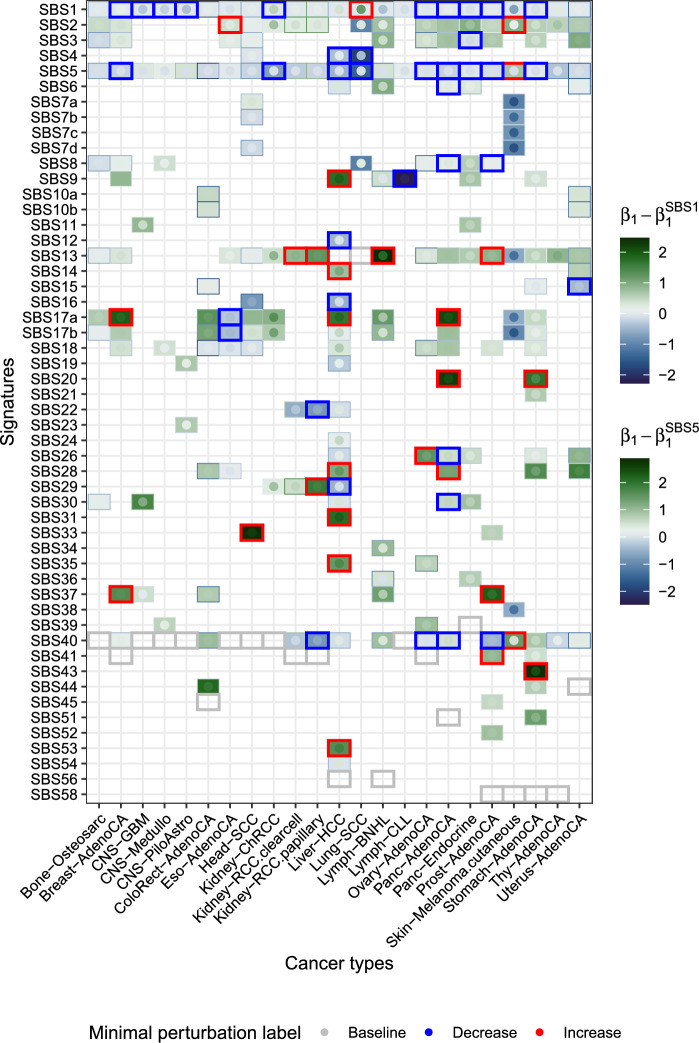
$\widehat{\varvec{\beta }}_1$ for each signature in each cancer type, subtracted from the $\widehat{\varvec{\beta }}_1$ which corresponds to clock signatures SBS1 (rectangles) and SBS5 (points inside rectangles). Green colours represent $\widehat{\varvec{\beta }}_1$ higher than those of clock signatures, and blue lower. They are signatures more subclonal and more clonal than clock signatures, respectively. The borders in certain cells indicate that the signature has been used as baseline (grey) or that according to the minimal perturbation analysis it is a signature that has increased (in red) or decreased (in blue) in absolute abundance

A less acute disagreement among clock signatures is found in the three CNS cancer types, where $\widehat{\varvec{\beta }}_1$ for SBS5 are too different from those of SBS1. Among the three CNS cancer types, only SBS1, SBS5 and SBS40 are signatures in common, and their pattern of differential abundance shows the same relative increase: $\widehat{\beta }_1^{\text {SBS1}}$ is always slightly lower than $\widehat{\beta }_1^{\text {SBS5}}$, and all remaining signatures have a higher $\widehat{\varvec{\beta }}_1$. Whereas in CNS-Medullo SBS1 and SBS5 correlate in the patient intercepts (Figs. S33, S34, S35), they do not in the other two cancer types. In CNS-GBM, SBS1 has a constant value of zero in the intercepts (i.e. no between-patient variability in the abundance of SBS1 with respect to SBS40). Contrarily, in all CNS cases the ratio of SBS5 and SBS40 varies greatly between patients. This finding applies across cancer types: we find that SBS1, SBS40 and SBS41 often do not contribute to the between-patient variability (see Kidney-RCC.papillary for the clearest example) whereas SBS5 does. These results are inconsistent with hypothesis that the random intercepts of SBS1 and SBS5 roughly track the age of the patients in all cancer types. Keeping in mind the discordant behaviour of SBS1 and SBS5 in the aforementioned cancer types, the comparison of any $\widehat{\varvec{\beta }}_1$ to $\widehat{\varvec{\beta }}_1^{\text {SBS1}}$ and $\widehat{\varvec{\beta }}_1^{\text {SBS5}}$ in the same cancer type is useful: where the belief that SBS1 and SBS5 represent signatures of constant mutation rate is justified, we can compare $\widehat{\beta }_1$ of signatures of interest with $\widehat{\beta }_1^{\text {SBS1}}$, $\widehat{\beta }_1^{\text {SBS5}}$ to determine the direction of change (i.e. an increase or decrease in the averaged mutation rate). In Fig. 7, the values plotted are those of $\widehat{\beta }_1$ for each signature in each cancer type, subtracted from $\widehat{\beta }_1^{\text {SBS1}}$ or $\widehat{\beta }_1^{\text {SBS5}}$. Green colours represent $\widehat{\varvec{\beta }}_1$ higher than those of clock signatures, and blue lower.

**General increase in signature activity** As an alternative to the comparison with signatures of a constant mutation rate, and assuming that the abundance of most signatures does not change, $\widehat{\varvec{\beta }}_1$ values can be compared to the median $\widehat{\varvec{\beta }}_1$ value across all signatures in a cancer type (Fig. S36, Sect. S5), classifying each active signature as a signature that increases, decreases, or does not change in absolute abundance. We call this the minimal perturbation framework, and the labels resulting from it are projected onto Fig. 7 and are largely concordant with the comparison to clock signatures: $\widehat{\beta }_1$ for signatures where the abundance decreases are mostly lower than $\widehat{\beta }_1^{\text {SBS1}}$ and $\widehat{\beta }_1^{\text {SBS5}}$, whereas for signatures where the abundance increases they are higher. Note that this approach works well only when the assumption that the abundance of most mutational processes does not change is met: we find several cases of SBS1 or SBS5 being labelled as signatures that decrease in abundance, which might simply reflect that numerous other signatures increase in abundance, making the amount of red boxes possibly conservative. APOBEC signatures SBS2 and SBS13 are labelled as signatures that increase in 2 and 4 cancer types respectively. SBS17a (u.a.) often increases, and is always more subclonal than SBS17b (u.a.). On occasion, they rank lower than SBS1/5 (Fig. S37). The tendency for clonality of SBS40 varies extraordinarily, implying that, as a very flat signature, it could be capturing signal from other signatures. Signatures hypothesised to increase in activity include SBS20 (POLD1 mutations) in both cancer types in which it is active, and SBS31 (platinum chemotherapy). Although generally low, $\widehat{\varvec{\beta }}_1^{\text {SBS1}}$ and $\widehat{\varvec{\beta }}_1^{\text {SBS5}}$ are not necessarily the lowest coefficients: signatures of undoubtedly exogenous origin (SBS7b and SBS7d, representing UV light) have consistently lower coefficients, as do SBS12 (u.a.), SBS15 (DMMR), SBS16 (u.a.), and SBS19 (u.a.), suggesting, whenever their aetiology is unknown, that they are, too, signatures of exogenous processes. SBS16 appears as clonal signature—suggesting that the mutational process it represents was active historically, before tumour onset—in the two cancer types where it is present, Head-SCC and Liver-HCC. It has been linked to alcohol consumption [38] but this is not included in COSMIC [9]. This is the signature that solely drives the random intercepts in Head-SCC (Fig. S34) indicating patient variability in its abundance, stratifying the samples into three groups according to these random intercepts (Fig. S33).

**Behaviour of APOBEC and associated signatures** Overall, there is good agreement in $\widehat{\varvec{\beta }}_1$ between the two APOBEC signatures SBS2 and SBS13, with discordant $\widehat{\varvec{\beta }}_1$ only in the two renal cell carcinoma cohorts (Kidney-RCC clearcell and papillary), as well as in Lymph-BNHL and Skin-Melanoma. These samples are the only ones in which the patient random intercepts of SBS2 and SBS13 are poorly correlated, with correlations ranging from − 0.03 to 0.36 (Fig. S35), indicating that patients who present high values of SBS2 do not present high values of SBS13, or conversely. APOBEC signatures have been considered to be active [14] or not [39] in previous studies of kidney cancer. Their low abundance, discordant $\widehat{\varvec{\beta }}_1$, and discordant contribution to the random intercepts suggest that, in these cancer types, SBS13 might not be. Judging the equivalent results in melanoma, SBS13 is active but SBS2 is not.

Beyond the agreements in clock and APOBEC signatures, the most noteworthy trends are those of SBS8 and SBS18 and their interplay with APOBEC signatures and HRD. The aetiology of SBS8 is uncertain, although it has been linked to HR and NER deficiency [9]. Although SBS18 has been linked to reactive oxygen species, its aetiology is not yet clear, and is associated with MUTYH-related signature SBS36 [9]. SBS18 aligns well with the APOBEC signatures except in the case where $\widehat{\varvec{\beta }}_1^{\text {SBS2}}$ is highest (Eso-AdenoCA). Similarly, $\widehat{\varvec{\beta }}_1^{\text {SBS18}}$ closely matches $\widehat{\varvec{\beta }}_1^{\text {SBS3}}$. SBS3 and SBS8 display markedly different $\widehat{\varvec{\beta }}_1$ (Fig. S38), although they always have intermediate values of $\widehat{\varvec{\beta }}_1$ compared to other signatures. Interestingly, $\widehat{\varvec{\beta }}_1^{\text {SBS2}}$ is consistently higher than $\widehat{\varvec{\beta }}_1^{\text {SBS8}}$. Such behaviour could be explained by at least four scenarios: by APOBEC initiation preceding a heightened mutation rate of SBS8, by a more subtle increase in the mutation rate of SBS8 than of SBS2, by an increase in SBS8 in only a fraction of clones (but an increase of SBS2 in all cells), or by an increase of SBS8 in a fraction of the patients (and a more common increase of SBS2). With very few exceptions, these signatures have intermediate values of $\widehat{\varvec{\beta }}_1$. In fact, in several cancer types we find several shared values of $\widehat{\varvec{\beta }}_1$ (seen as a plateau in the sorted $\widehat{\varvec{\beta }}_1$ of Fig. S32). In Panc-AdenoCA one such plateau includes SBS2, SBS13, SBS3 and SBS18 - these signatures are also included in the same plateau in Breast-AdenoCA.

**Tissue-specific differential abundance** Within-tissues analysis reveals which aspects of differential abundance are shared between cancer types of the same tissue and which are not. Often, the set of active signatures itself varies drastically within a tissue or organ (in the case of the three CNS samples, the two pancreatic samples, between Kidney-ChRCC and the two Kidney-RCC samples). This is to be expected in cases where the cell of origin differs.

With the pre-selected set of active signatures, CNS samples differ in every aspect except in the correlation between SBS1 and SBS40 in CNS-GBM and CNS-Medullo (Fig. S34) – a correlation which is absent in CNS-Medullo – and the ordering of $\widehat{\varvec{\beta }}_1^{\text {SBS1}}$ and $\widehat{\varvec{\beta }}_1^{\text {SBS5}}$. Indeed, we find that upon re-extraction of signature exposures and considering all signatures present in the tissue (Fig. S39) the three CNS samples show only moderate commonalities in abundance ($\widehat{\varvec{\beta }}_0$) and none in differential abundance ($\widehat{\varvec{\beta }}_1$). The two kidney RCC samples are very consistent in their differential abundance, showing SBS22 – the aristolochic acid exposure [9] – as the signature of lowest $\widehat{\varvec{\beta }}_1$, together with SBS40, and followed closely by the clock signatures. Upon signature re-extraction with all signatures present in the kidney, the general abundance is moderately similar even between ChRCC and RCC, but the differential abundance results differ completely. In the two RCC cancer types the opposite is true: their abundances differ slightly (e.g. in SBS29), but their differential abundance results are in perfect alignment except for, again, SBS29. In the case of Panc-AdenoCA, the random intercepts are clear in showing that, unlike in Panc-Endocrine, SBS3 plays an important role in explaining the variability between patients. In the analysis of all signatures, we find a similar pattern to the ChRCC/RCC comparison in the pancreatic samples where, although the abundances are only markedly different in SBS3, SBS9 and SBS36 (showing shared and possibly tissue-related mutational processes that were active early in tumour development, or prior to it), the differential abundance patterns are not shared.

## Deployment of *CompSign* in cancer genomics

***CompSign*** **for the analysis of two-group matched data** In summary, applied to the PCAWG dataset, the mixed-effects Dirichlet-multinomial model implemented in *CompSign* finds ubiquitous differential abundance between clonal and subclonal mutations, capturing similar dynamics in signatures known to be related, as well as discovering new correlations. Besides differential abundance, we find differential precision, with higher dispersion levels in subclonal signature exposures. In this work we begin to explore the true correlations between mutational signatures, and not the correlations induced by the compositional nature of these data. Validation of these results either experimentally or computationally and with orthogonal data would be of interest – notably, *in vitro* experiments for the analysis of mutational processes can help establish when changes in their absolute abundance occur.

**Extension of the analysis to other use cases: chromosome analysis** Compositional models of differential abundance can help answer a variety of questions in cancer genomics, and we indicate how *CompSign* can be used in two settings different from the two-group design with matched observations. In the first extension, we explore differential abundance of signatures across chromosomes in the Prost-AdenoCA cohort. Matched information about patients is not used, making the analysis comparable to a simple comparison of each chromosome to a baseline chromosome (here, Chr1). The equivalent of $\varvec{\beta }_1$ are $\varvec{\beta }_{\text {Chr2}}$, $\varvec{\beta }_{\text {Chr3}}$, etc. We use the model FEDMsinglelambda, which is our implementation of Dirichlet-multinomial regression without random effects that includes a single precision parameter $\varvec{\lambda }$ shared across all patients (Sect. S6.1). Inspecting the fitted $\widehat{\varvec{\beta }}$ values (Fig. S40) suggests not only that signature abundance varies greatly across chromosomes, but that chromosomes can be clustered accordingly (Fig. S41), and even allows us to point to some signatures underpinning these chromosome-specific patterns of abundance, such as SBS3 (HRD), the relative abundance of which can sometimes be much lower (e.g. in Chr2 or Chr3) or much higher (e.g. Chr17, Chr19) than in Chr1.

**Extension of the analysis to other use cases: CCF regression** Still leveraging information across samples, in the second use case we seek to investigate if more granular information on the clonal structure of the sample can be used. This use case is inspired by TrackSig, which orders mutations by CCF and groups them in bins, looking for changepoints in mutational signatures along the series of bins. Using PCAWG data for Lung-SCC, we have sorted mutations in decreasing CCF and grouped them in bins of 100 mutations. Exposures are extracted for each bin. We fit a regression model with two covariates: a baseline shared among patients and bins, and the mean CCF for each of the bins. The model used is diagREDMpatientlambda from *CompSign*, in which each patient has a corresponding $\lambda$ parameter, and patient uncorrelated random effects are used. In this use case, $\varvec{\beta }_1$ indicates the relationship between signature log-ratios and CCF. Fig. S42 shows such coefficients, where the relative abundance of SBS4 (linked to tobacco smoke) is shown to be positively correlated with CCF, corroborating the previous knowledge that it is a clonal signature. Such mixed-effects regression models show good performance in simulated data and can be seen as an equivalent of TrackSig where information is shared across samples (Fig. S43, Sect. S6.1.1).

**Guidelines for using** ***CompSign*** To help in the deployment of these methods, we give some guidance for each step of the pipeline. The signature exposure matrix $\textbf{Y}$ is taken as input, but several choices need to be made in order to generate this matrix from a sequence of mutations, chiefly whether to extract signatures *de novo*—in which case the number of signatures will specified, possibly by computing the optimal number of signatures based on metrics (see e.g. [40])—or to fit the signatures from a known set of signature definitions, such as the COSMIC signatures. The latter approach is computationally simpler and has the advantage that signature definitions and aetiologies are curated constantly in the COSMIC database, aiding in the interpretation of the output of the model. We show that the differential abundance results of the model are robust to the strategy used for signature extraction, but ultimately the signature extraction method is deserving of a conversation in its own right and needs to take into consideration the cancer type, the origin of the mutation data, and a variety of technical and clinical covariates. It is important not to include signatures which are nearly always zero. Although the multinomial model and its extensions support zero (count) exposures, including a signature which is never active or only active in the clonal and subclonal group can lead to problems in estimating $\varvec{\beta }_0$ and $\varvec{\beta }_1$. Alternative methods in the field, such as HiLDA and TCSM, have opted for extracting signatures *de novo* and determining differential abundance in a joint model. Indeed, one of the limitations of the models shown here is that there is no error propagation between signature extraction and differential abundance testing. On the other hand, in our models it is the incorporation of random effects, dispersion parameters, freedom in the choice of input signature exposures, and a flexible fixed-effects structure that allow for an in-depth characterisation of the differential abundance parameters.

**Model choice** The next step is the choice of model from the models considered here (chiefly, diagREDM, fullREDM). We recommend the use of diagREDM for most purposes, especially if the interest is in the analysis of differential abundance (and not, for instance, in the patient random effects that contribute to signature abundance). Especially, in cases where the number of samples is low and the number of mutations is high, estimating the covariance matrix in fullREDM can be difficult and slow. We have introduced several *CompSign* models, with additional ones listed in Table S2. For instance, in regression settings, models with a single $\lambda$ parameter are appropriate. The choice of baseline signature can be important for interpretability of the results. If using COSMIC signatures, we recommend using SBS1, SBS5 or SBS40 as baseline signatures, as they are active in most cancer types (simplifying between-cancer type comparison of coefficients), and they are suggested to be signatures of constant mutation rate. If there is good reason, these exposures could even be amalgamated together, and used as a single baseline category.

Finally, although the models give differential abundance results as a single *p*-value, they also provide the $\widehat{\varvec{\beta }}_1$ coefficients, as well as all other coefficients in the model, which can be analysed in their own right. To account for the compositional nature of the model we suggest being careful in drawing any conclusions on the direction of change of signatures, and instead plotting the $\widehat{\varvec{\beta }}_1$ coefficients with their error and in increasing order, which gives an indication of which signatures experience a higher increase relative to each other, and makes clear which signatures lie at the extremes of the plot. In the same plot, it can be useful to see the position of the $\widehat{\varvec{\beta }}_1$ coefficients for any signatures of interest relative to the position of $\widehat{\varvec{\beta }}^{\text {SBS1}}$ and $\widehat{\varvec{\beta }}^{\text {SBS5}}$, if those are not used as the baseline.

**Number of mutations and samples required for satisfactory results** We have shown that the differential abundance analysis and signature extraction suffer from a reduction in the number of mutations or samples. In practice, for most PCAWG cancer types, more than 60% of mutations or samples can be removed yielding practically undistinguishable $\widehat{\varvec{\beta }}_1$ values. The number of mutations commonly found in whole exome sequencing data correspond to the number of mutations found at the lower end of the number of mutations in the PCAWG cohort, where results on bias, coverage and robustness to small perturbations to the data were satisfactory, and so were in simulated data where as few as 20 samples and 50 mutations were included. Lack of convergence may arise if the ratio of signatures to samples is high, if the number of mutations is low, or if the dispersion levels are very high. 

**Future applications** Besides the two-group design with matched samples from PCAWG, we have shown how a regression model can be used to model several timepoints of tumour development, and to interrogate chromosomes separately. The ease in applicability of *CompSign* suggests immediate future directions along this line of research, such as to deploy the proposed models to other cancer cohorts, and to extend the covariates considered - to test the relevance of clinical covariates, or to compare exposures of groups of samples with and without certain driver mutations, before and after relapse, or before and after treatment. The models presented are publicly available and readily applicable to other types of latently compositional count data to determine differential abundance between groups, or to perform other types of multivariate regression.

**Table 1:** Methods for determining changes in the mutational spectrum

**Methods for determining differential abundance in mutational signatures**			
**Name**	**Model**	**Caveats**	**Reference**
HiLDA	Signature extraction (LDA) followed by Dirichlet-multinomial	No mixed effects, local test not compositionally-minded	[23]
Tumor Covariate Signature Model (TCSM)	Signature extraction and test through simulation	No mixed effects, test through simulation without interpretation of signatures in log-ratios	[24]

**Methods for studying the dynamics of mutational signatures**			
TrackSig	Multinomial model to determine changepoints in bins of exposures	Compositional model but non-compositional interpretation of dynamics	[41]
Clonesig	Clonal inference using potential differences in the exposures of clones as aid	Not a method for detecting differential abundance as such	[42]
Log-odds of signature-specific exposures	Log-ratio of normalised exposures for each signature between early and late mutations, and between clonal and subclonal mutations	Non-compositional; changes in log-ratios of a signature can be due to changes in other signatures	[21]

**Table 2:** List of notation

***Matrix factorisation***		
Data	*N*	Number of samples or observations (input of NMF)
	*K*	Number of signatures
	*F*	Number of mutational categories or features
	$\textbf{V}$	($F \times N$) Count matrix of categorised mutations
Parameters	$\textbf{Y}^{*}$	($K \times N$) Matrix of exposures. For the sections below, $\textbf{Y}=\textbf{Y}^{*\top }$
	$\textbf{S}$	($F \times K$) matrix of signature definitions
Indices	*f*	Index for features (mutational types such as ACA$\rightarrow$ATA, for instance)

***Models of differential abundance***		
Data	*N*	Number of observations with $N\ge N_s$
	$N_s$	Number of patients, equivalent to the number of samples
	*K*	Number of categories/signatures
	*P*	Number of fixed effect covariates
	*Q*	Number of precision covariates
	$\textbf{X}$	($N \times P$) covariate matrix with P=2 in our case, without loss of generality
	$\textbf{Z}$	($N \times N_s$) boolean matrix for random intercepts
	$\textbf{D}$	($N \times Q$) precision covariate matrix with $\textbf{D}=\textbf{X}$ in our case
	$\textbf{Y}$	($N \times K$) response matrix of exposures
	$T_j$	Mutational toll of observation *j*. $T_j=\sum _{k'}Y_{jk'}$
	$M_i^{(1)}$, $M_i^{(2)}$	($T_i^{(1)} \times 1$) sequence of mutations of each observation of patient *i*
Parameters	$\varvec{\beta }$	($P \times (K-1)$) Matrix of coefficients for fixed effects
	$\mathbf {\Sigma _{\varvec{\beta }_p}}$	($(K-1) \times (K-1)$) covariance matrix of the *p*th row of $\varvec{\beta }$
	$\textbf{U}$	($N_s \times (K-1)$) matrix of coefficients for random effects
	$\mathbf {\Sigma }$	($(K-1) \times (K-1)$) random effect covariance matrix
	$\varvec{\lambda }$	($Q \times 1$) vector of precision parameters
Indices	*i*	Index for patients
	*j*	Index for observations (patient and group combinations)
	*p*	Index for covariates
	$k'$	Index for signatures/categories
	*k*	Index for signatures/categories (log-ratio, or the first $K-1$)
	*l*	Index for mutations in an observation
Random variables	$\varvec{\alpha }$	Parameter for the Dirichlet or Dirichlet-multinomial
	$\bar{\varvec{\alpha }}$	Parameter for the Dirichlet or Dirichlet-multinomial (compositional)

***Other notation***		
	$\mathbf {0_K}$	Vector of zeros of length *K*
	$\textbf{Y}, \mathbf {y_j}, {y_{jk}}$	Notation for matrices, vectors and scalars.
	*P*, *p*	Dimensions, indices
Simulations	$\pi$	For simulations C1-3, mixing proportion. Lower values indicate no mixing (no differential abundance).

## Supplementary Information

Supplementary file 1.

## Data Availability

The code for reproducing the results of this paper can be found in https://github.com/lm687/CompSign-results and all datasets and inference results in https://zenodo.org/records/10546525.
