# Supplementary material for: A Dirichlet-multinomial mixed model for determining differential abundance of mutational signatures
Source: BMC Bioinformatics. 2025 Feb 18;26:59. doi: 10.1186/s12859-025-06055-x (PMC11837616; doi:10.1186/s12859-025-06055-x)
Supplement: Supplementary file 1 [file 12859_2025_6055_MOESM1_ESM.pdf]

Supplementary figures

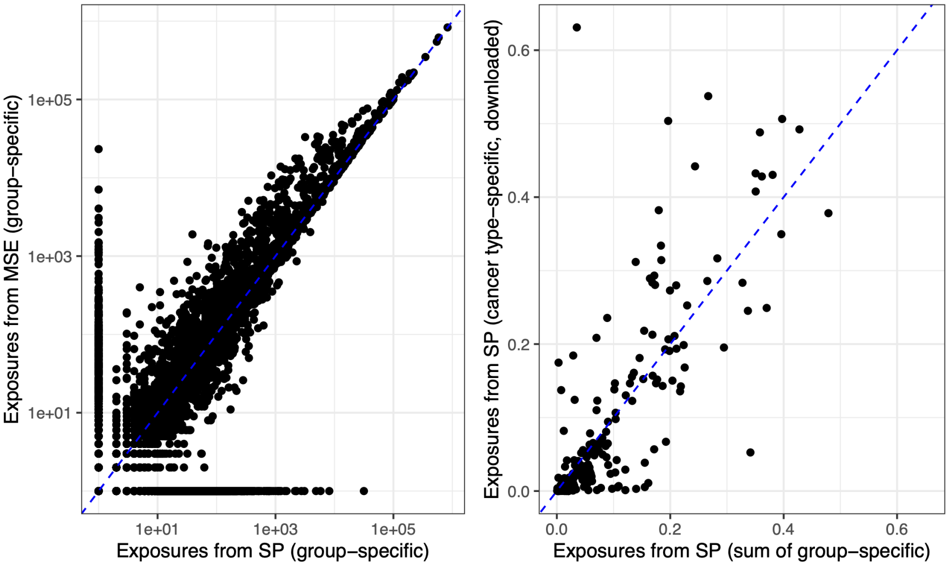

Figure S1: Left: comparison of exposures that have been extracted using quadratic programming (QP) and MSE. Right: comparison of exposures at the cancer-type level to those published in [14], in which each point represents a signature in a cancer type.

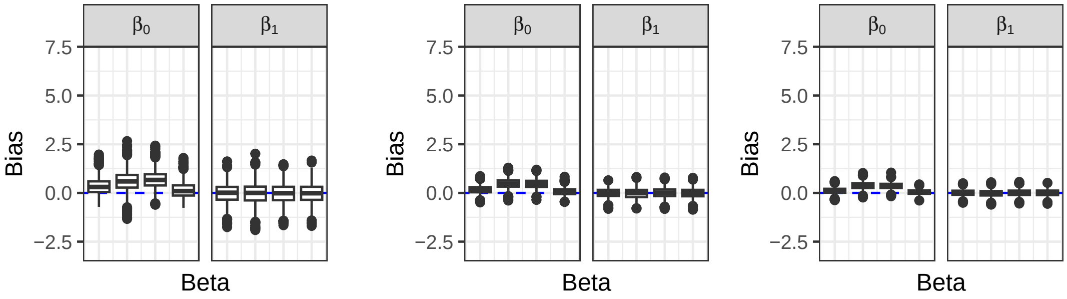

(a) Bias, $\lambda = 2$ (A1) (b) Bias, $\lambda = 20$ (A2) (c) Bias, $\lambda = 100$ (A3)

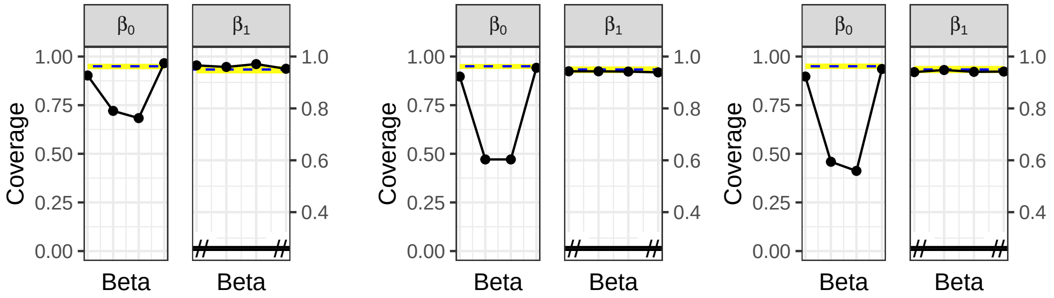

(d) Coverage, $\lambda = 2$ (A1) (e) Coverage, $\lambda = 20$ (A2) (f) Coverage, $\lambda = 100$ (A3)

Figure S2: Estimation and inference when (wrongly) assuming a restricted model with non-correlated mixed-effects DM (`diagREDM`). Data simulated using a correlated DM with random intercepts with parameters $N_s = 200$, $\sum_j y_{lj} = 180 \; \forall \; l$, $d = 5$ (A1-A3). $\boldsymbol{\beta}_1$ is well recovered, but $\boldsymbol{\beta}_0$ is not, for the inability to model the correlated abundances of signatures in the first group. In all cases of precision (all three columns) are $\boldsymbol{\beta}_0$ poorly estimated, whilst the estimates of $\boldsymbol{\beta}_1$ have no bias and good coverage. The highlighted area is the $0.025 - 0.975$ quantile of 1000 binomial draws of the same size as the data with $p = 0.95$.

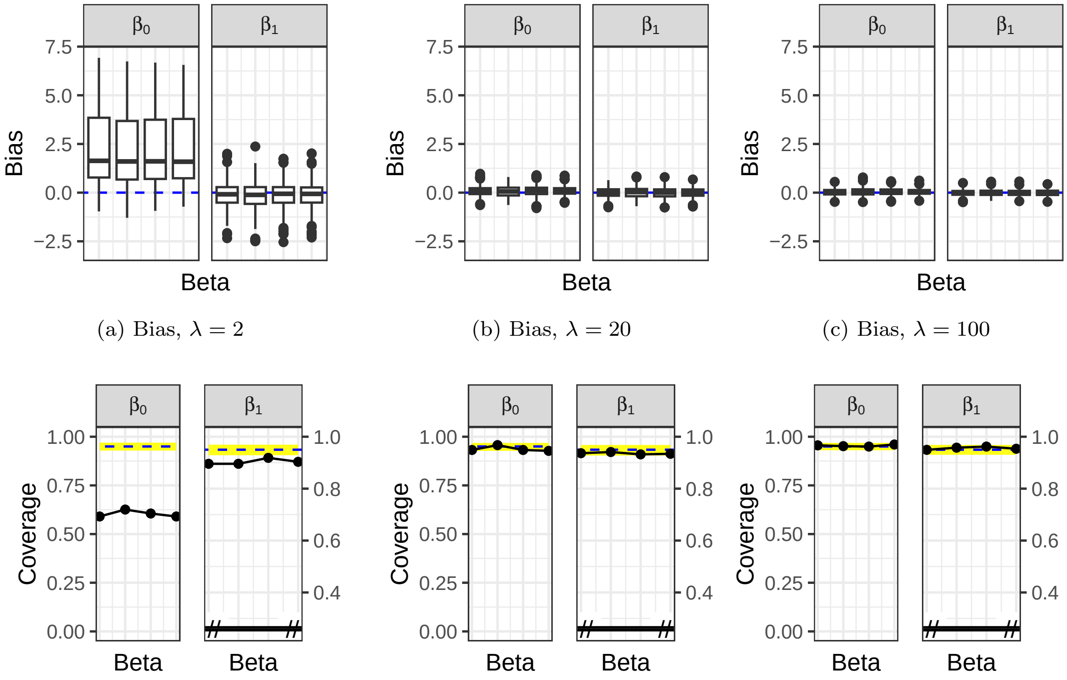

(a) Bias, $\lambda = 2$ (b) Bias, $\lambda = 20$ (c) Bias, $\lambda = 100$

(d) Coverage, $\lambda = 2$ (e) Coverage, $\lambda = 20$ (f) Coverage, $\lambda = 100$

Figure S3: Estimation and inference when (correctly) assuming a model with correlated mixed-effects DM (`fullREDM`). Data simulated using a correlated DM with random intercepts with parameters $N_s = 200$, $\sum_j y_{lj} = 180 \ \forall \ l$, $d = 5$ (A1-A3). Both $\boldsymbol{\beta}_0$ and $\boldsymbol{\beta}_1$ are well recovered, except in the scenario of low precision (first column; $\lambda = 2$). The highlighted area is the $0.025 - 0.975$ quantile of 1000 binomial draws of the same size as the data with $p = 0.95$.

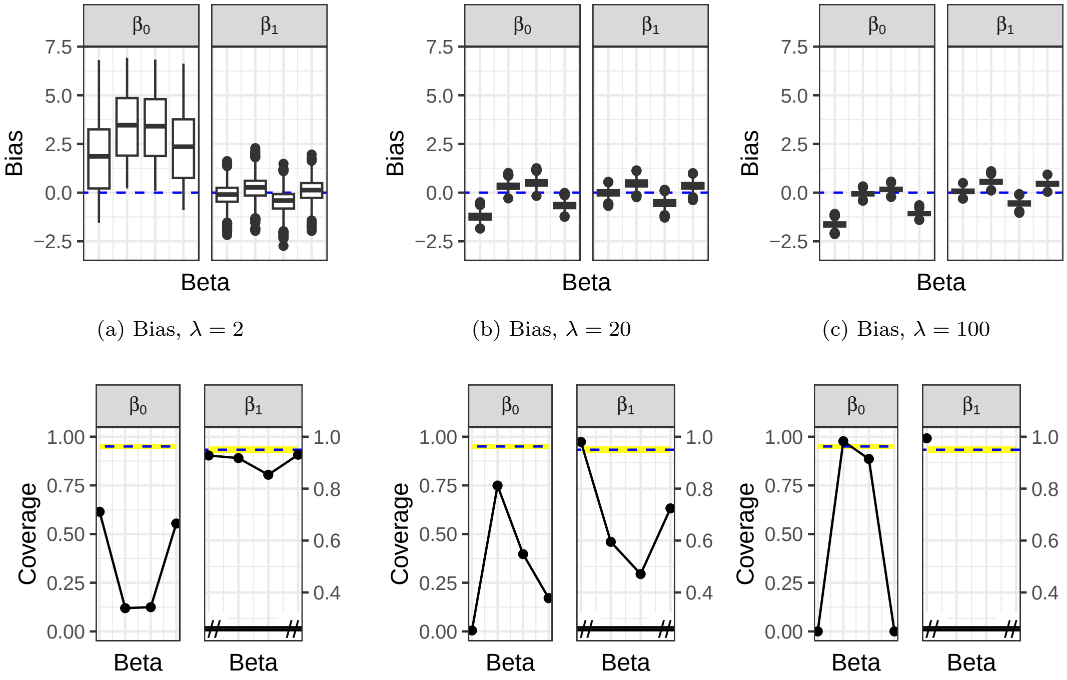

(a) Bias, $\lambda = 2$ (b) Bias, $\lambda = 20$ (c) Bias, $\lambda = 100$

(d) Coverage, $\lambda = 2$ (e) Coverage, $\lambda = 20$ (f) Coverage, $\lambda = 100$

Figure S4: Estimation and inference when (wrongly) assuming a restricted model with a single intercept per patient (`singleREDM`). Data simulated using a correlated DM with random intercepts with parameters $N_s = 200$, $\sum_j y_{lj} = 180 \; \forall \; l$, $d = 5$ (A1-A3). Neither $\boldsymbol{\beta}_0$ nor $\boldsymbol{\beta}_1$ are well recovered, in all three levels of precision (all three columns). The highlighted area is the $0.025 - 0.975$ quantile of 1000 binomial draws of the same size as the data with $p = 0.95$.

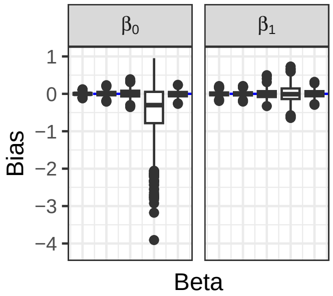

(a) Bias, `diagREDM`

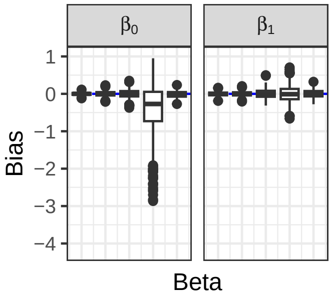

(b) Bias, `fullREDM`

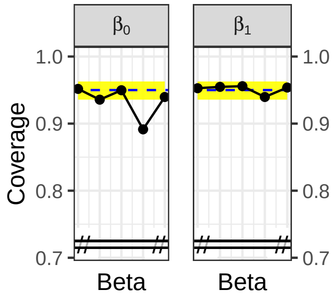

(c) Coverage, `diagREDM`

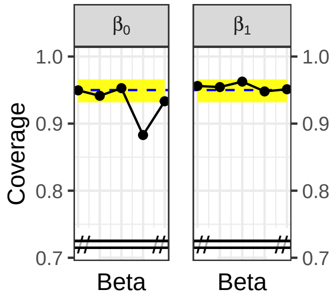

(d) Coverage, `fullREDM`

Figure S5: Estimation and inference using un-correlated mixed-effects DM (`diagREDM`) and correlated mixed-effects DM (`fullREDM`) for simulation (B1). Data simulated using previously-estimated parameters from the CNS-GBM cohort, with $N_s = 200$, no correlations, and a shared $\boldsymbol{\lambda}$ (average between the two estimated λ). All remaining parameters are taken as estimated. Given that uncorrelated data are simulated, both models give the same results for bias and coverage. Both $\boldsymbol{\beta}_0$ and $\boldsymbol{\beta}_1$ are well recovered, although the penultimate element of $\boldsymbol{\beta}_0$ has some bias. The highlighted area is the $0.025-0.975$ quantile of 1000 binomial draws of the same size as the data with $p = 0.95$.

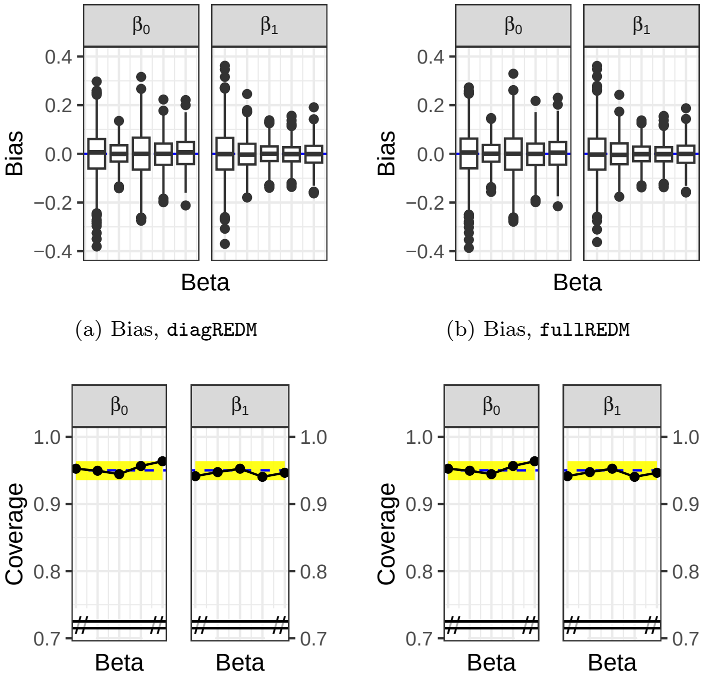

(a) Bias, `diagREDM`

(b) Bias, `fullREDM`

(c) Coverage, `diagREDM`

(d) Coverage, `fullREDM`

Figure S6: Estimation and inference using un-correlated mixed-effects DM (`diagREDM`) and correlated mixed-effects DM (`fullREDM`) for simulation (B2). Data simulated using previously-estimated parameters from the Lung-SCC cohort, with $N_s = 200$, no correlations, and a shared $\boldsymbol{\lambda}$. All remaining parameters are taken as estimated. Given that uncorrelated data are simulated, both models give the same results for bias and coverage. Bias and coverage results are satisfactory for both $\boldsymbol{\beta}_0$ and $\boldsymbol{\beta}_1$. The highlighted area is the $0.025 - 0.975$ quantile of 1000 binomial draws of the same size as the data with $p = 0.95$.

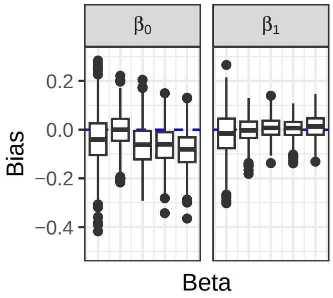

(a) Bias, `diagREDM`

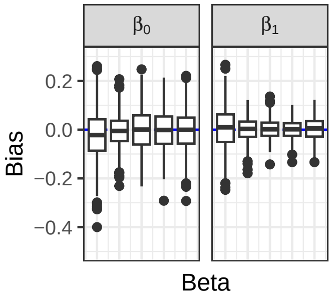

(b) Bias, `fullREDM`

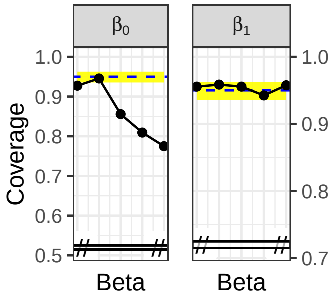

(c) Coverage, `diagREDM`

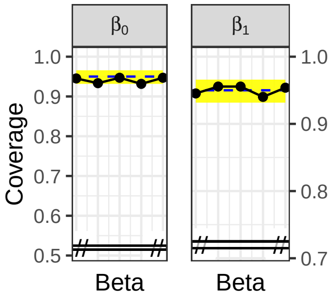

(d) Coverage, `fullREDM`

Figure S7: Estimation and inference using un-correlated mixed-effects DM (`diagREDM`) and correlated mixed-effects DM (`fullREDM`) for simulation (B3). Data simulated using previously-estimated parameters from the Lung-SCC cohort, with $N_s = 200$ and a shared $\boldsymbol{\lambda}$ (average between the two estimated λ). All remaining parameters are taken as estimated, including correlations. The results for bias and coverage differ between the models owing to the presence of correlations. Bias and coverage results are satisfactory in both cases for $\boldsymbol{\beta}_1$, but there is bias and low coverage for $\boldsymbol{\beta}_0$ in the non-correlated `diagREDM` model. The highlighted area is the $0.025 - 0.975$ quantile of 1000 binomial draws of the same size as the data with $p = 0.95$.

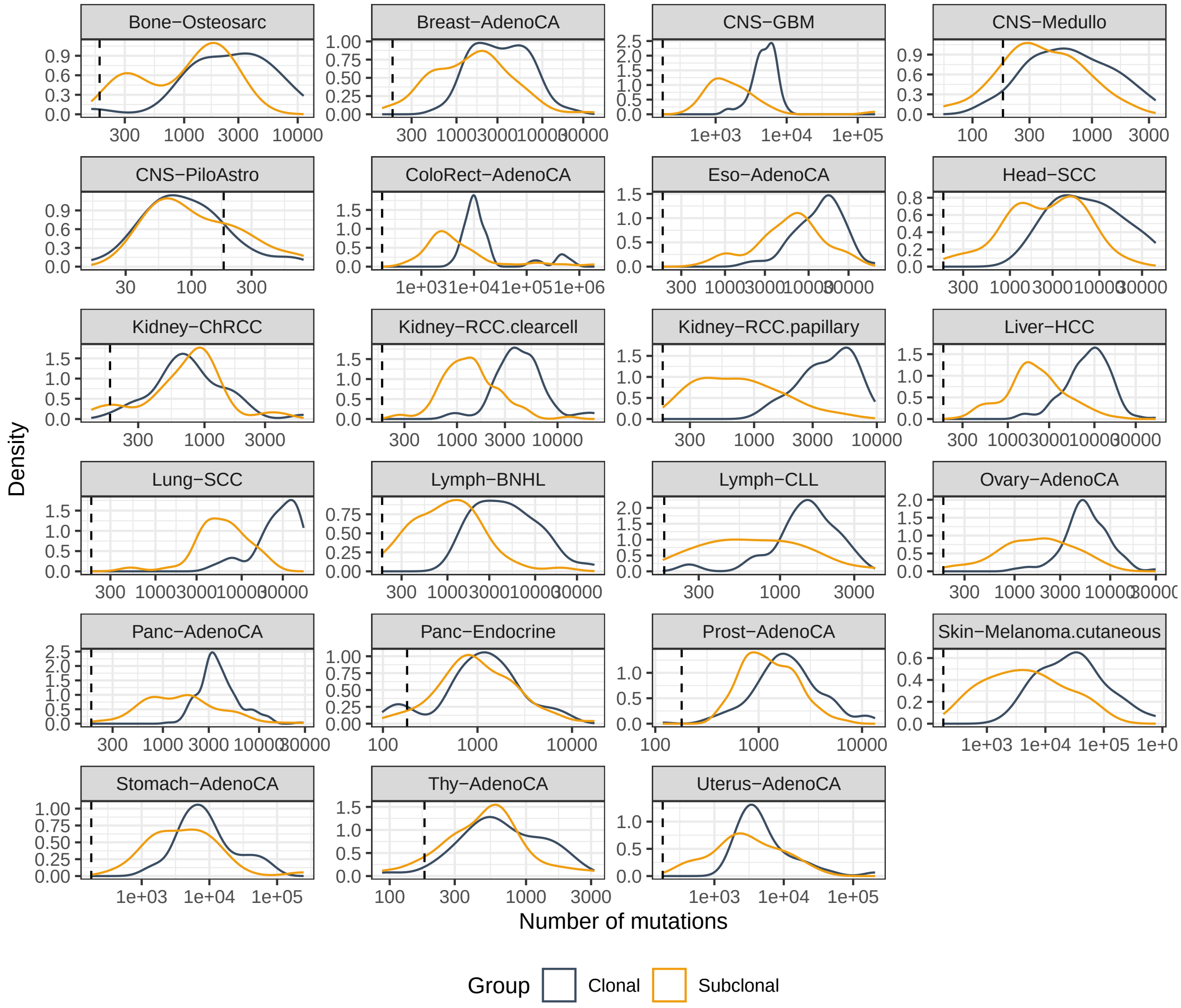

Figure S8: Density plots of the number of mutations in each group and patient, for each cancer type cohort in the PCAWG dataset.

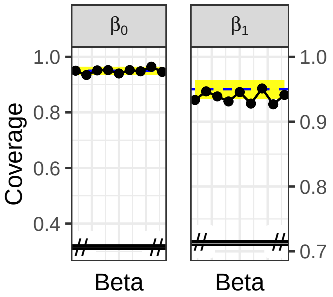

(a) Coverage, observed T, `fullREDM`

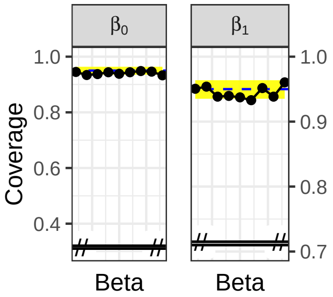

(b) Coverage, lower T, `fullREDM`

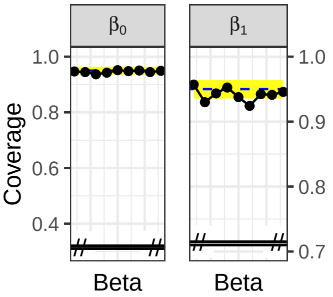

(c) Coverage, lowest T, `fullREDM`

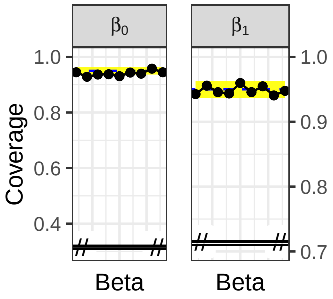

(d) Coverage, observed T, `diagREDM`

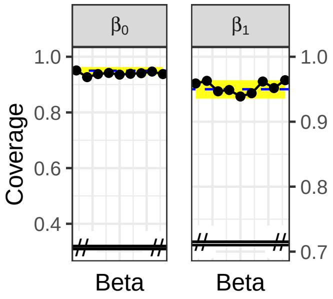

(e) Coverage, lower T, `diagREDM`

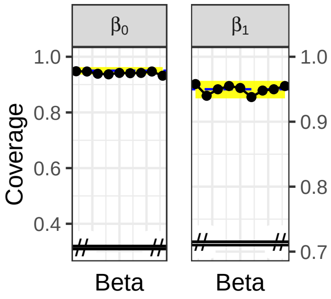

(f) Coverage, lowest T, `diagREDM`

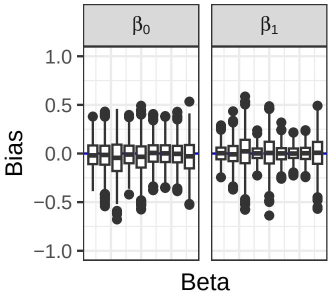

(g) Bias, observed T, `fullREDM`

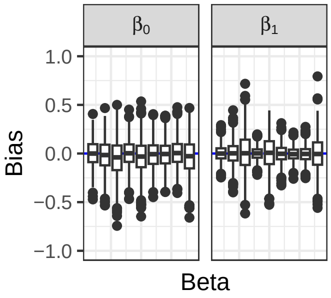

(h) Bias, lower T, `fullREDM`

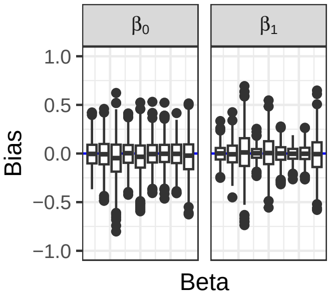

(i) Bias, lowest$T$, `fullREDM`

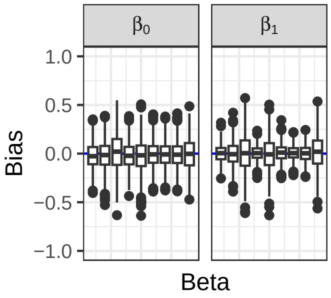

(j) Bias, T, `diagREDM`

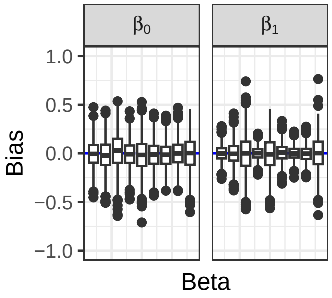

(k) Bias, lower T, `diagREDM`

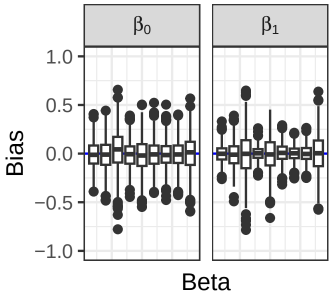

(l) Bias, lowest T, `diagREDM`

Figure S9: Bias and coverage as the number of mutations T in the simulations decreases (B4). Data simulated using previously-estimated parameters from the Prost-AdenoCA cohort, selecting the four most abundant signatures.

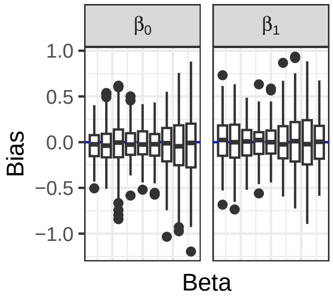

(a) Bias, Bone-Osteosarc

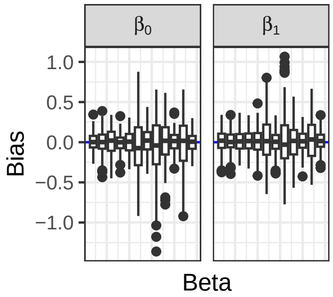

(b) Bias, Breast-AdenoCA

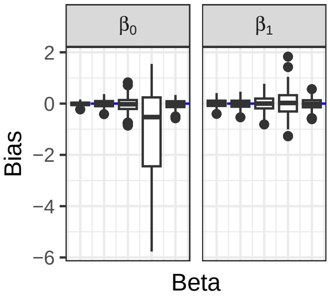

(c) Bias, CNS-GBM

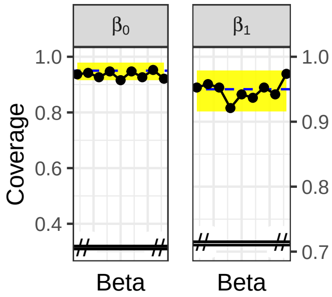

(d) Coverage, Bone-Osteosarc

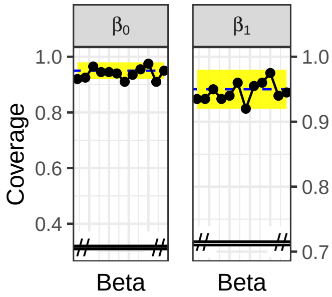

(e) Coverage, Breast-AdenoCA

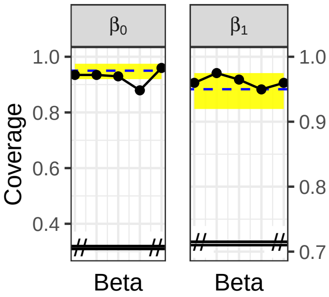

(f) Coverage, CNS-GBM

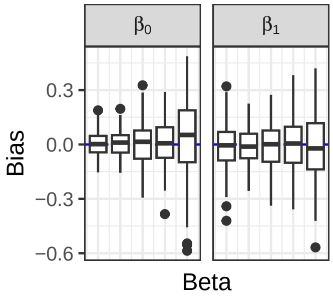

(g) Bias, CNS-Medullo

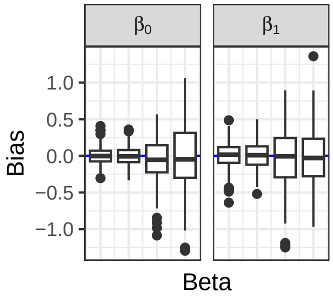

(h) Bias, CNS-PiloAstro

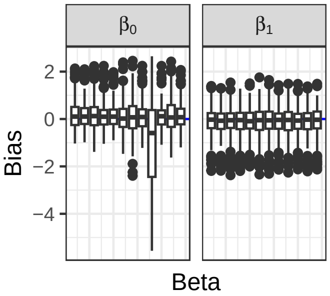

(i) Bias, ColoRect-AdenoCA

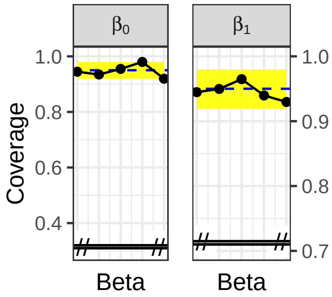

(j) Coverage, CNS-Medullo

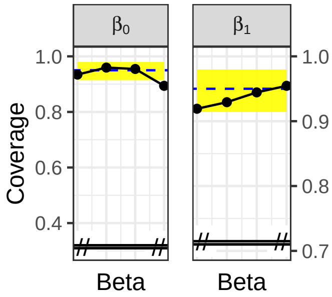

(k) Coverage, CNS-PiloAstro

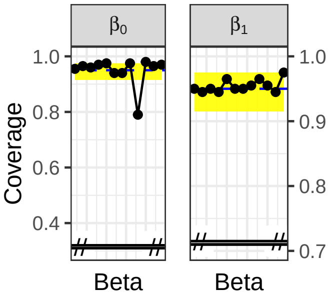

(l) Coverage, ColoRect-AdenoCA

Figure S10: Bias and coverage for data from PCAWG. Figure continues on next page.

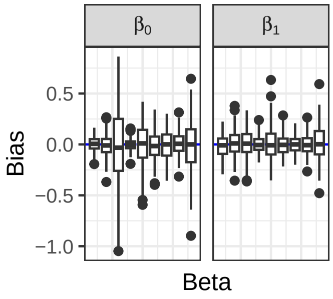

(m) Bias, Eso-AdenoCA

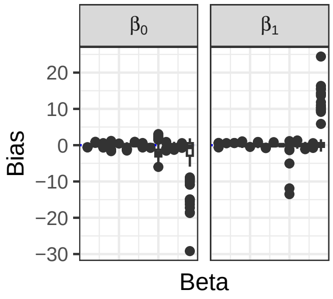

(n) Bias, Head-SCC

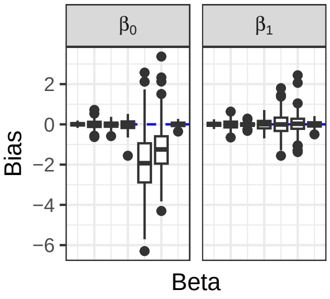

(o) Bias, Kidney-ChRCC

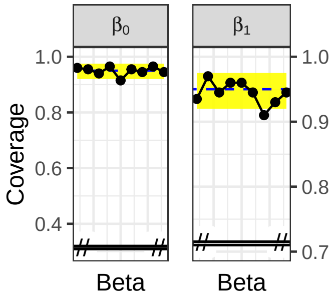

(p) Coverage, Eso-AdenoCA

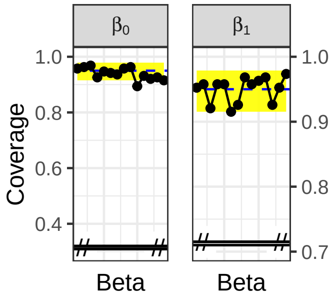

(q) Coverage, Head-SCC

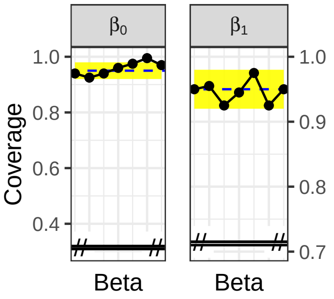

(r) Coverage, Kidney-ChRCC

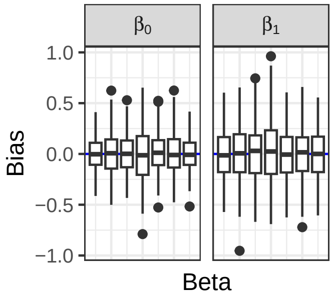

(s) Bias, Kidney-RCC clearcell

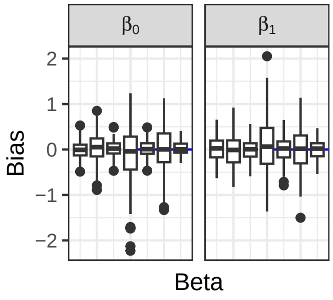

(t) Bias, Kidney-RCC papillary

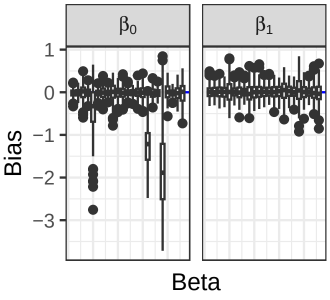

(u) Bias, Liver-HCC

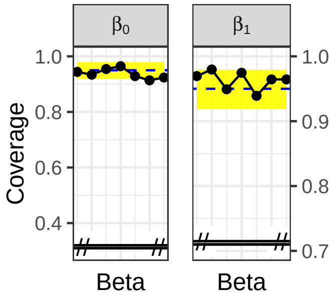

(v) Coverage, Kidney-RCC clearcell

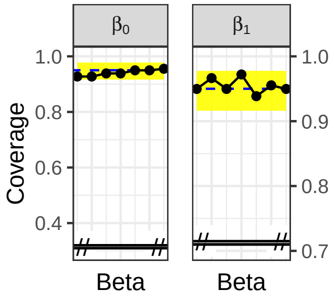

(w) Coverage, Kidney-RCC papillary

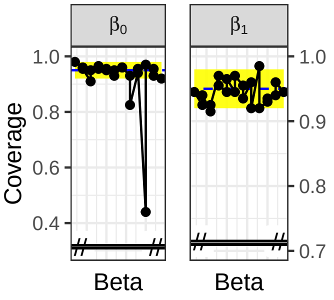

(x) Coverage, Liver-HCC

Figure S10: Bias and coverage for data from PCAWG. Figure continues on next page.

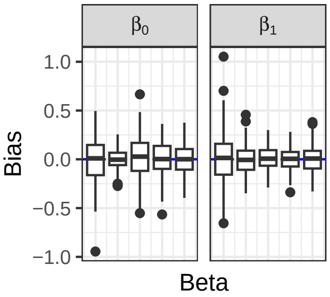

(y) Bias, Lung-SCC

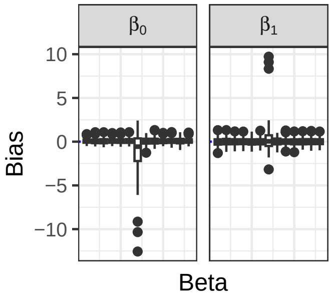

(z) Bias, Lymph-BNHL

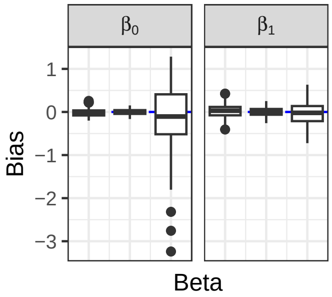

(aa) Bias, Lymph-CLL

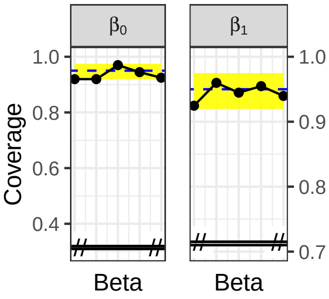

(ab) Coverage, Lung-SCC

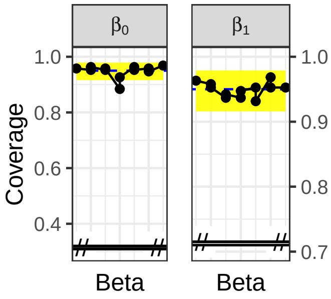

(ac) Coverage, Lymph-BNHL

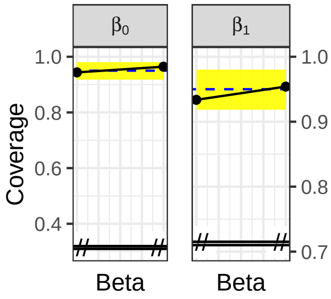

(ad) Coverage, Lymph-CLL

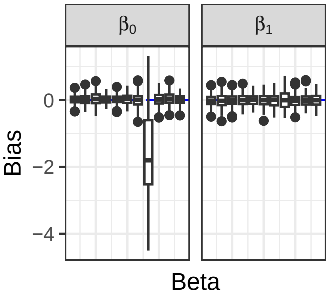

(ae) Bias, Ovary-AdenoCA

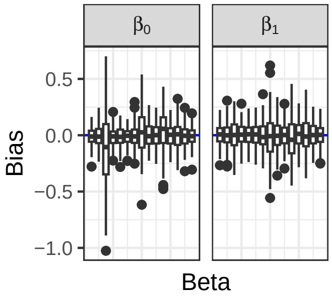

(af) Bias, Panc-AdenoCA

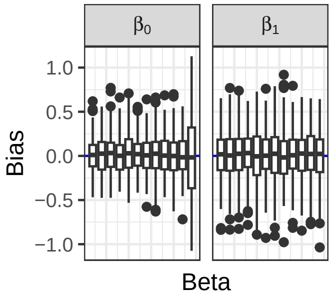

(ag) Bias, Panc-Endocrine

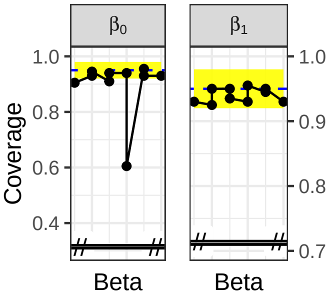

(ah) Coverage, Ovary-AdenoCA

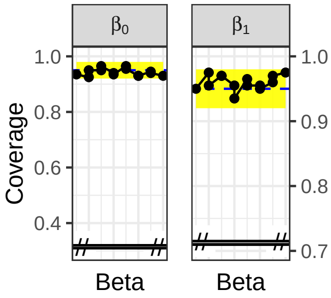

(ai) Coverage, Panc-AdenoCA

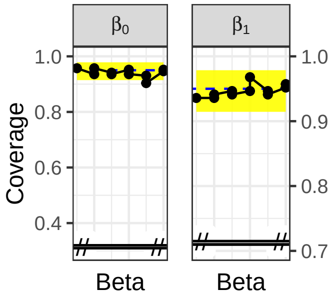

(aj) Coverage, Panc-Endocrine

Figure S10: Bias and coverage for data from PCAWG. Figure continues on next page.

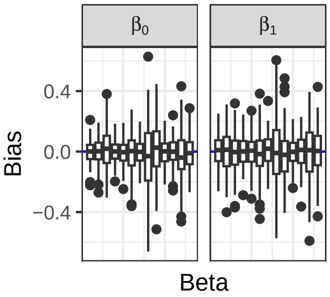

(ak) Bias, Prost-AdenoCA

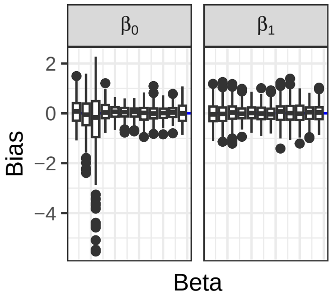

(al) Bias, Skin-Melanoma cutaneous

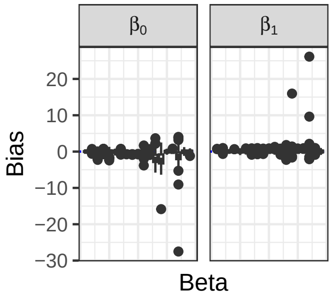

(am) Bias, Stomach-AdenoCA

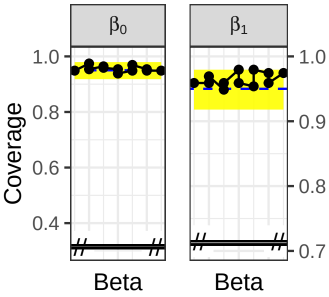

(an) Coverage, Prost-AdenoCA

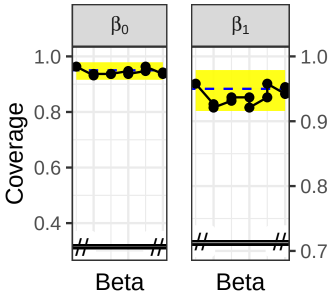

(ao) Coverage, Skin-Melanoma cutaneous

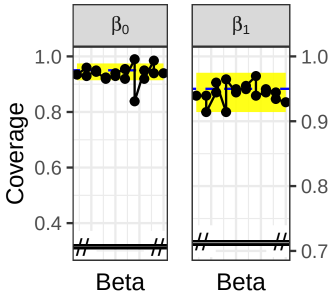

(ap) Coverage, Stomach-AdenoCA

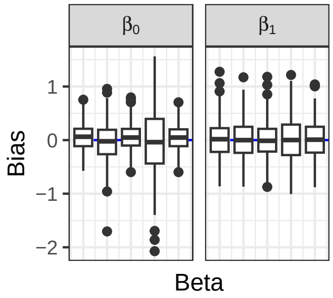

(aq) Bias, Thy-AdenoCA

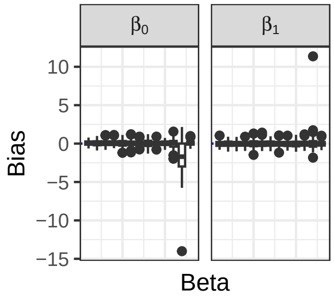

(ar) Bias, Uterus-AdenoCA

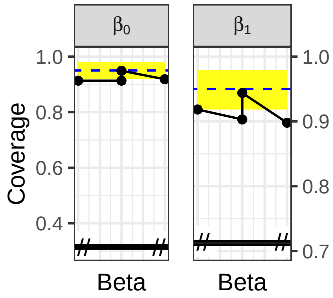

(as) Coverage, Thy-AdenoCA

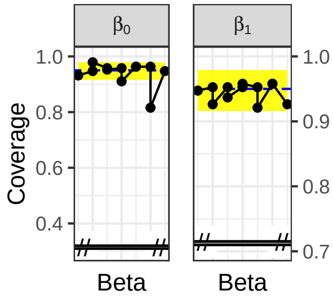

(at) Coverage, Uterus-AdenoCA

Figure S10: Bias and coverage for all cohorts in PCAWG, when data are simulated using fitted $\boldsymbol{\beta}$, $\boldsymbol{\lambda}$, and uncorrelated covariance matrices from `diagREDM`, and mutations are drawn from the median number of mutations in the clonal and subclonal groups of the observed datasets. These are the bias and coverage for the model `diagREDM`.

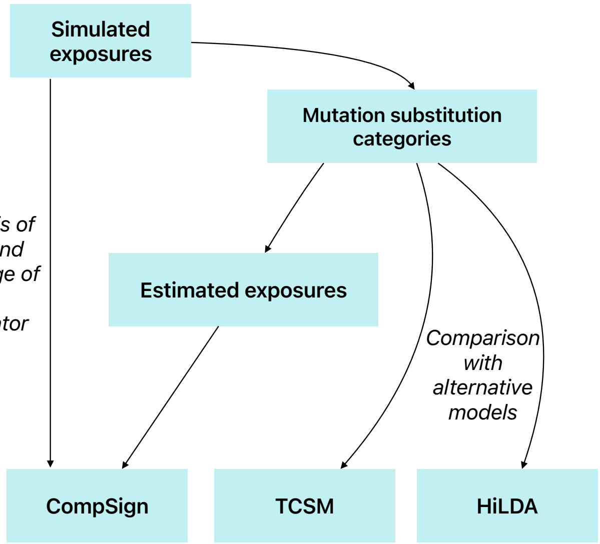

Figure S11: Framework for simulating data which can be used as input for TCSM and HiLDA, as well as `diagREDM` or other *CompSign* models. Exposures are simulated and from which trinucleotide mutations are drawn. Those are either used as input for TCSM or HiLDA, or signatures are re-extracted from them to be used as input for *CompSign* models. In the analysis of bias, data are simulated from the model.

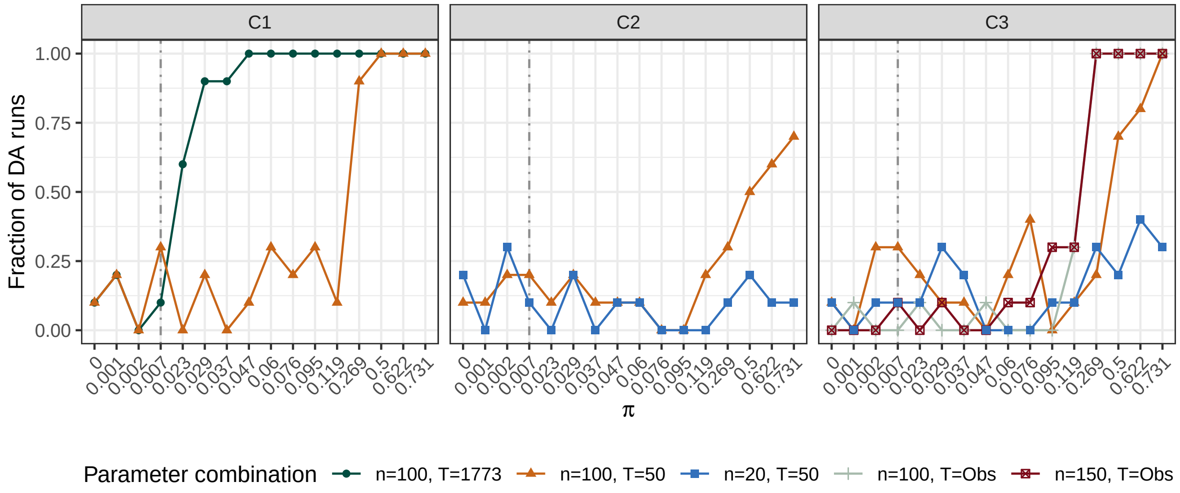

Figure S12: Results of `diagREDM` in simulations C1-C3, with varying parameters T and N_s. On the y axis, the fraction of differentially abundant runs at a significance level of 0.05, as the mixing proportion π increases (x axis). Both an increase in the number of mutations (first facet) and in the number of patient samples (second facet) leads to higher statistical power.

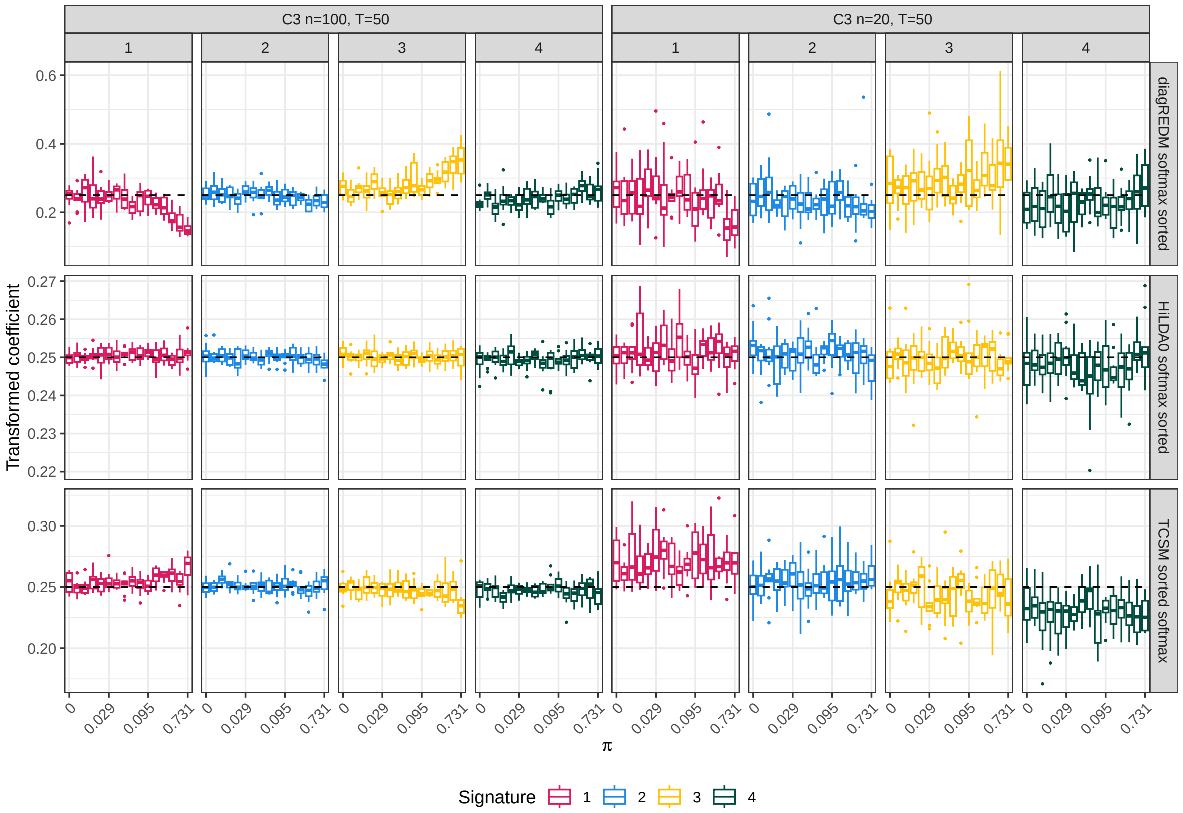

Figure S13: Comparison of coefficients that represent differential abundance for the models under consideration: `diagREDM`, HiLDA, and TCSM, in simulation C3. In the case of `diagREDM`, the signatures represent the ground truth signatures used for simulation (SBS1, SBS5, SBS9, SBS40).

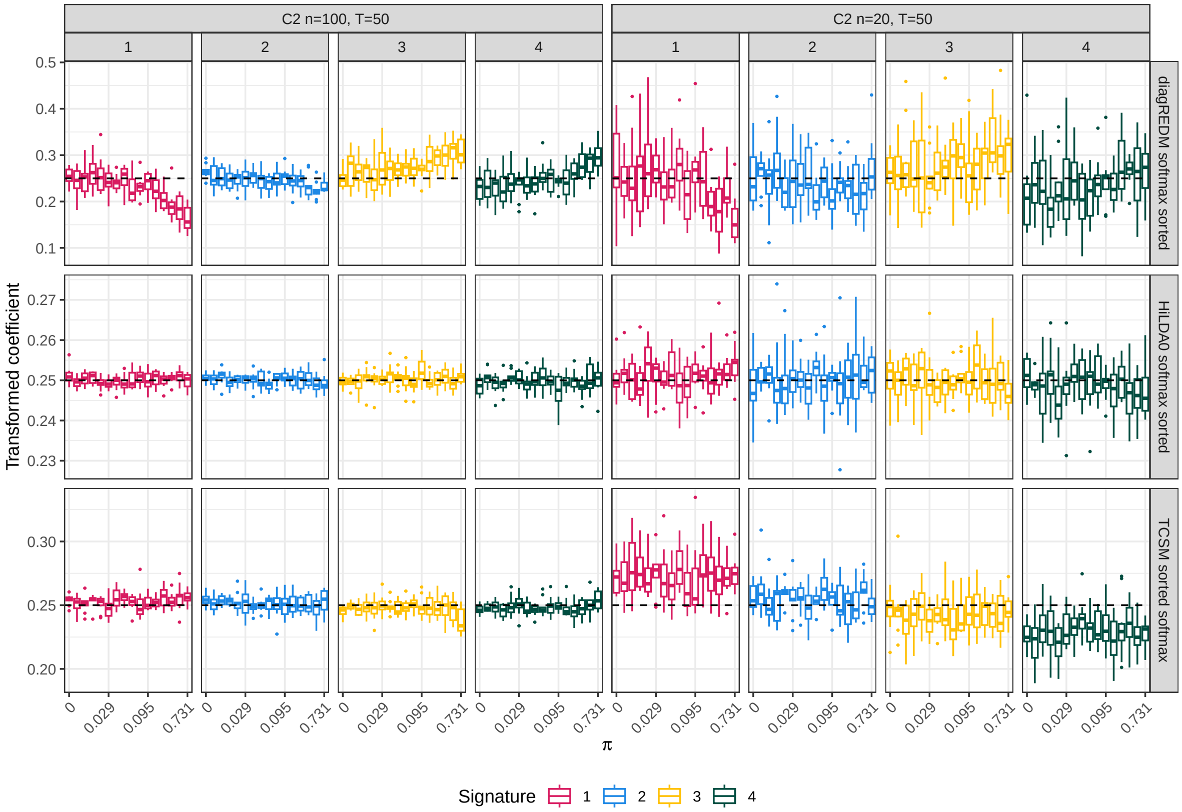

Figure S14: Comparison of coefficients that represent differential abundance for the models under consideration: `diagREDM`, HiLDA, and TCSM, in simulation C2. Note, for `diagREDM`, a poorer signal in C2 than in C3, as data in C3 are simulated with patient-specific intercepts which `diagREDM` models, increasing its statistical power. In the case of `diagREDM`, the signatures represent the ground truth signatures used for simulation (SBS1, SBS5, SBS9, SBS40).

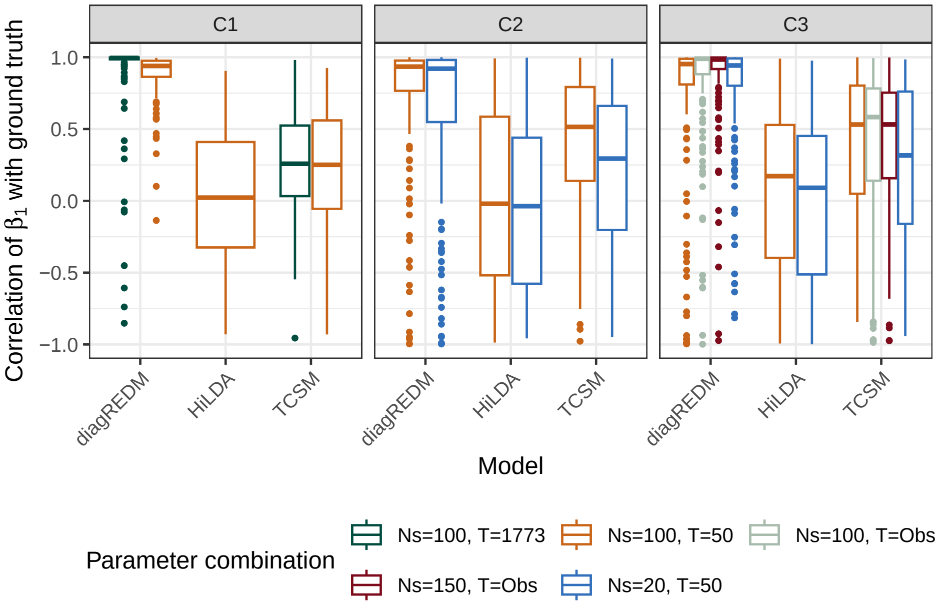

(a) Correlations between the estimated $\boldsymbol{\beta}_1$ and ground-truth $\boldsymbol{\beta}_1$.

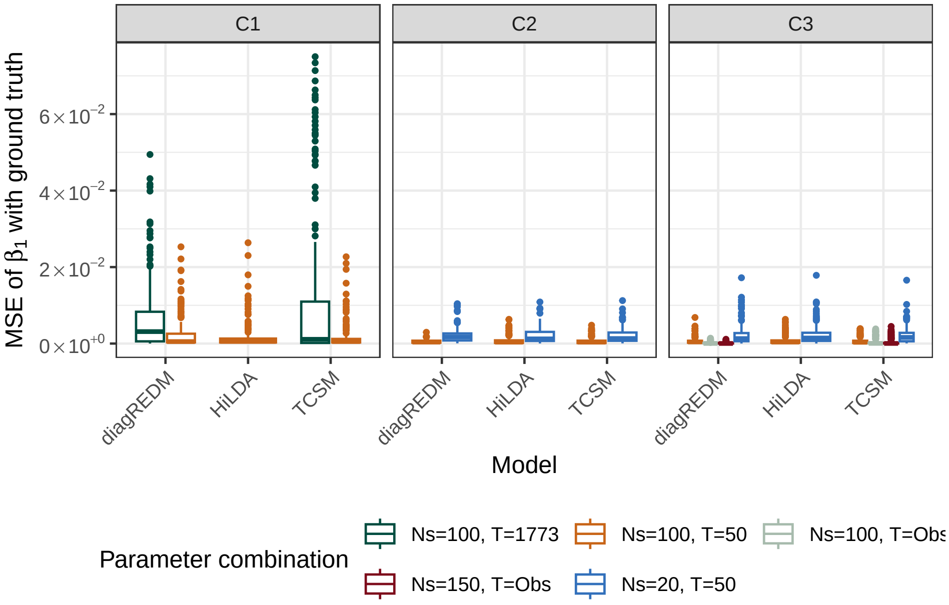

(b) MSE between the estimated $\boldsymbol{\beta}_1$ and ground-truth $\boldsymbol{\beta}_1$.

Figure S15: Correlations and mean-squared error (MSE) between the estimated $\boldsymbol{\beta}_1$ from `diagREDM`, HiLDA or TCSM, with the $\boldsymbol{\beta}_1$ extracted from the signature matrices used in the simulation of datasets C1-3. In all cases the coefficients have been softmax-transformed.

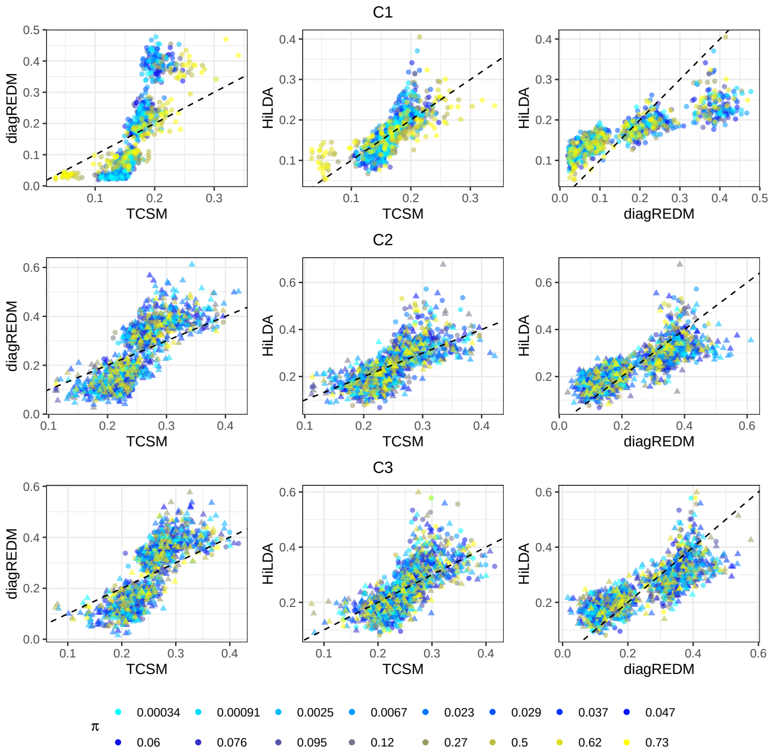

Figure S16: Comparison of abundances in the clonal group of simulated samples, for each of the three models and in simulated datasets C1-3. Values further away from the identity line indicate higher discrepancies in signature abundance. Note the case in the first and third facets, where sparse signatures are simulated in the highest value of π (yellow) but where TCSM and HiLDA overestimate these abundances.

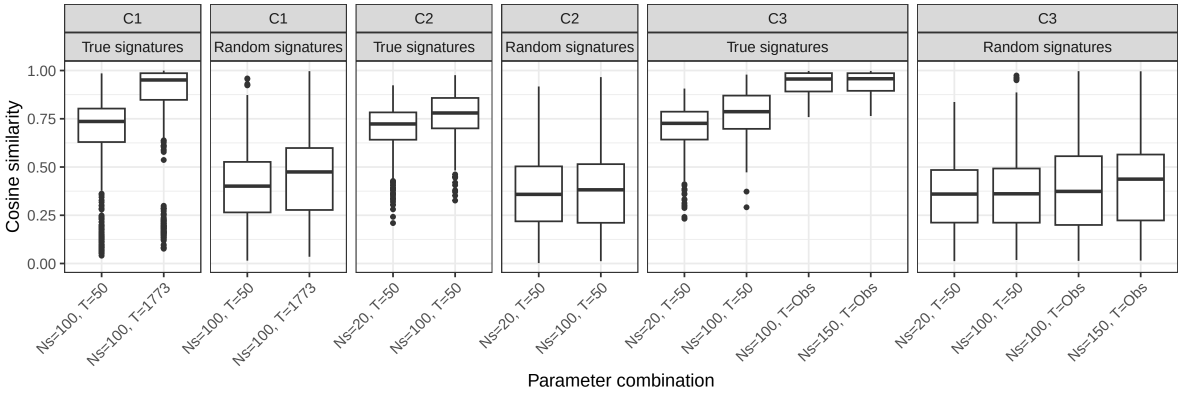

Figure S17: Results of signature extraction TCSM in simulations C1-C3. Higher cosine similarities indicate a better recovery of the ground-truth signatures used to simulate the input data for TCSM. The cosine similarities between the extracted signatures and the ground truth signatures are compared to the cosine similarities between extracted signatures and a set of COSMIC signatures chosen at random. With higher numbers of mutations T, signature reconstruction improves in all cases, and so does with a higher number of patient samples N_s. For each combination of number of samples N_s, number of mutations T, and datasets, the results for all values of π have been grouped together, as they did not influence the cosine similarity except for a slightly decrease in simulation C1 with $N_s = 100$ and $T = 1773$ as π increases (not shown).

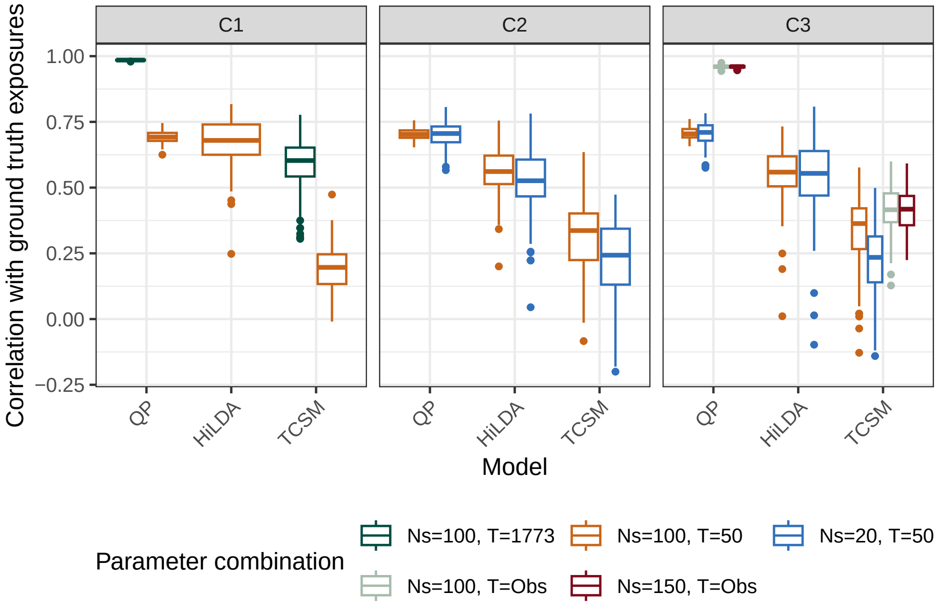

Figure S18: Pearson correlation between the ground truth exposures from the simulation and either the re-extracted exposures (from quadratic programming) used for `diagREDM`, the estimated exposures from HiLDA, or the estimated exposures from TCSM. The re-extracted exposures from quadratic programming are much more representative of the ground truth exposures than TCSM exposures, and (often) HiLDA exposures. For quadratic programming, the number of mutations used is also a determinant factor for correct signature extraction; provided the number is representative of the number of mutations T in the dataset (in this case, this corresponds to the highest values of T in C1 and C3), the exposures are perfectly recovered.

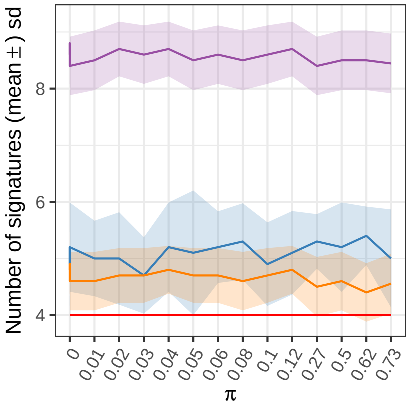

(a) Number of signatures determined by each active signature selection strategy.

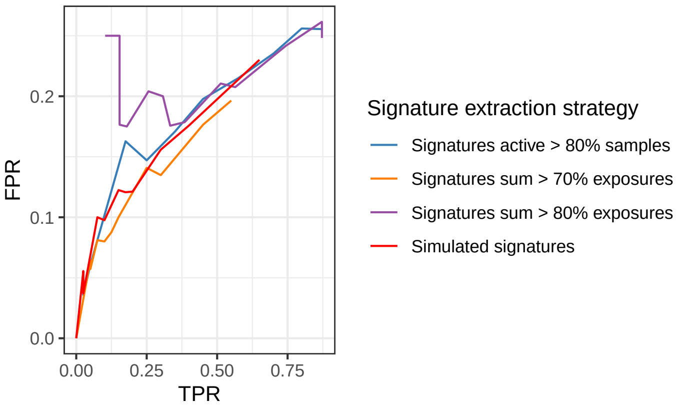

(b) ROC curve for each active-signature selection strategy.

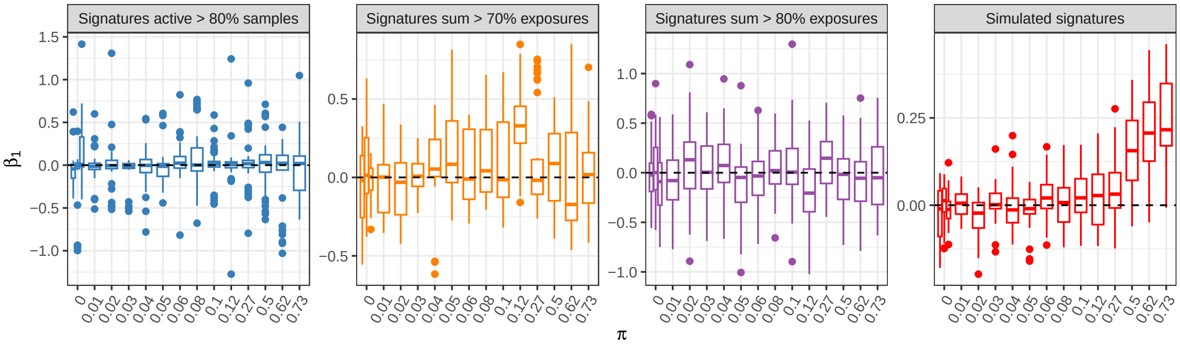

(c) $\widehat{\boldsymbol{\beta}}_1$ estimated using `diagREDM` on exposures extracted following each active-signature selection strategy.

Figure S19: Assessment of the `diagREDM` model when several strategies for selecting the set of active signatures are used. The results of differential abundance are robst to the set of signatures used (ROC curve), although as signatures are extracted *de novo* in the first three cases of (c) they are not shown to increase together with π as the original set of signatures does.

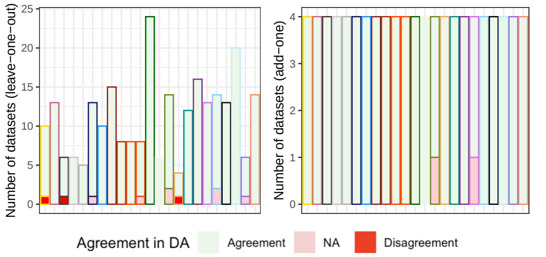

(a) Agreement in differential abundance for simulation D1a (signatures extracted with QP).

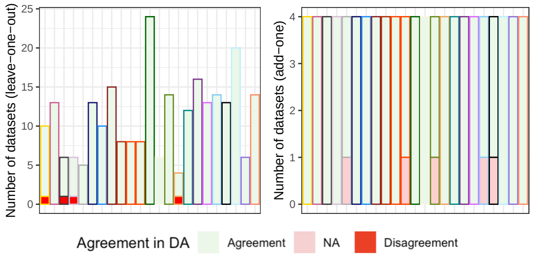

(b) Agreement in differential abundance for simulation D1a (signatures extracted with mutSigExtractor).

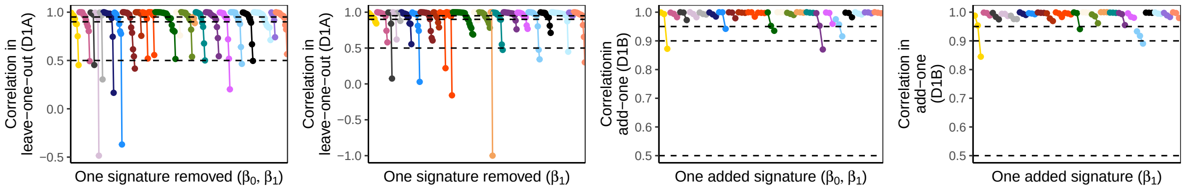

(c) Correlation of $\boldsymbol{\beta}$ for simulation D1a (signatures extracted with QP).

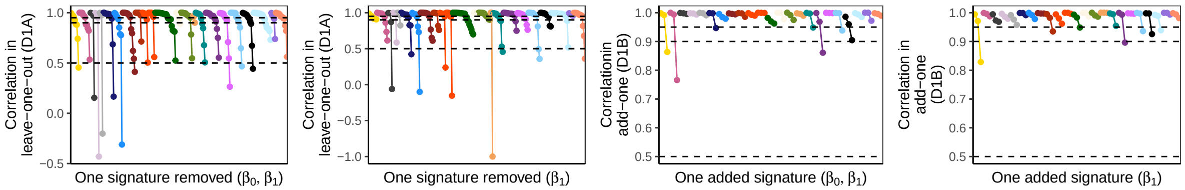

(d) Correlation of $\boldsymbol{\beta}$ for simulation D1a (signatures extracted with mutSigExtractor).

Bone-Osteosarc, Breast-AdenoCA, CNS-GBM, CNS-Medullo, CNS-PiloAstro, ColoRect-AdenoCA, Eso-AdenoCA, Head-SCC, Kidney-ChRCC, Kidney-RCC.clearcell, Kidney-RCC.papillary, Liver-HCC, Lung-SCC, Lymph-BNHL, Lymph-CLL, Ovary-AdenoCA, Panc-AdenoCA, Panc-Endocrine, Prost-AdenoCA, Skin-Melanoma.cutaneous, Stomach-AdenoCA, Thy-AdenoCA, Uterus-AdenoCA

Figure S20: Results from simulation D1A and D1B. Figures S20a and S20b: We count the number of simulations within each cancer type in which removing one signature at a time (left) or adding one additional signature (right) yields results in agreement with the unmodified dataset (in pale green), in disagreement (in red), or non-convergent results (in pale red). Figures S20c and S20d: correlations between the estimates of $\boldsymbol{\beta}$ in D1A and D1B to the estimates of $\boldsymbol{\beta}$ from the original set of active signatures. Within each cancer type, correlations have been sorted in decreasing order. We note that the correlations are very high in the vast majority of cases, especially when quadratic programming has been used to extract them.

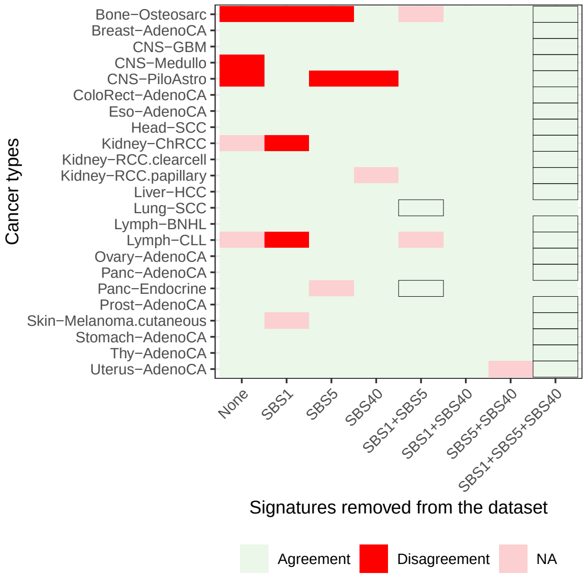

Figure S21: Results from simulation D1C. Agreement (green) or disagreement (red) between the original dataset and datasets where each combination of {SBS1,SBS5,SSB40} are included as active signatures.

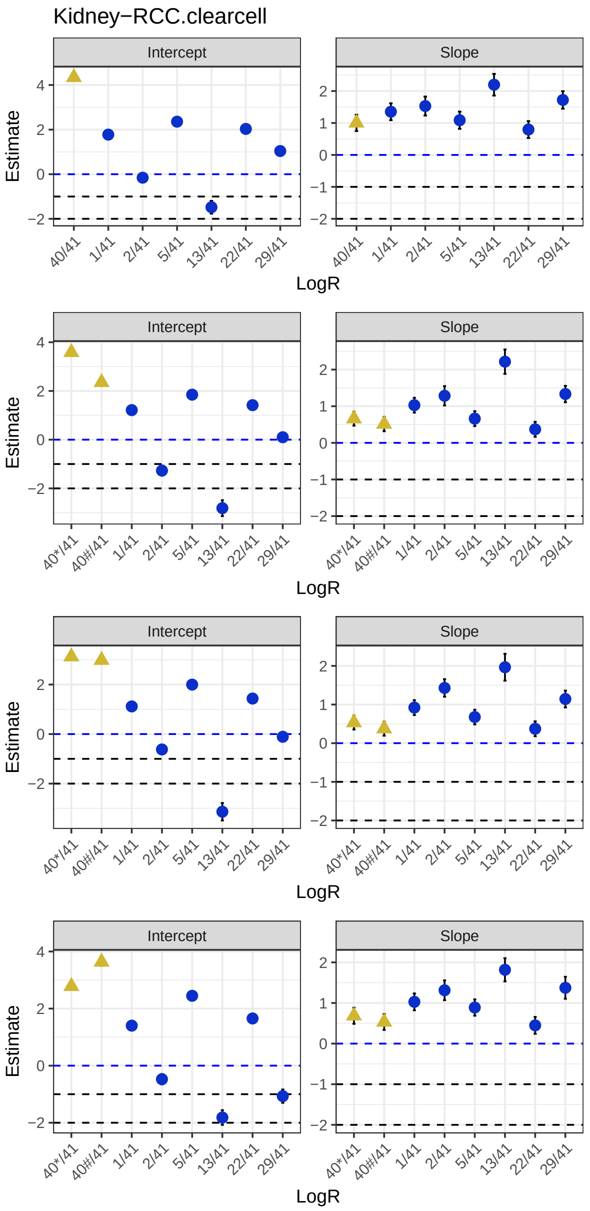

Figure S22: Results for simulation D1D, where signatures selected form the PCAWG datasets are split into two other signatures. Estimates of $\boldsymbol{\beta}_0$ and $\boldsymbol{\beta}_1$ for an example dataset in D1D.

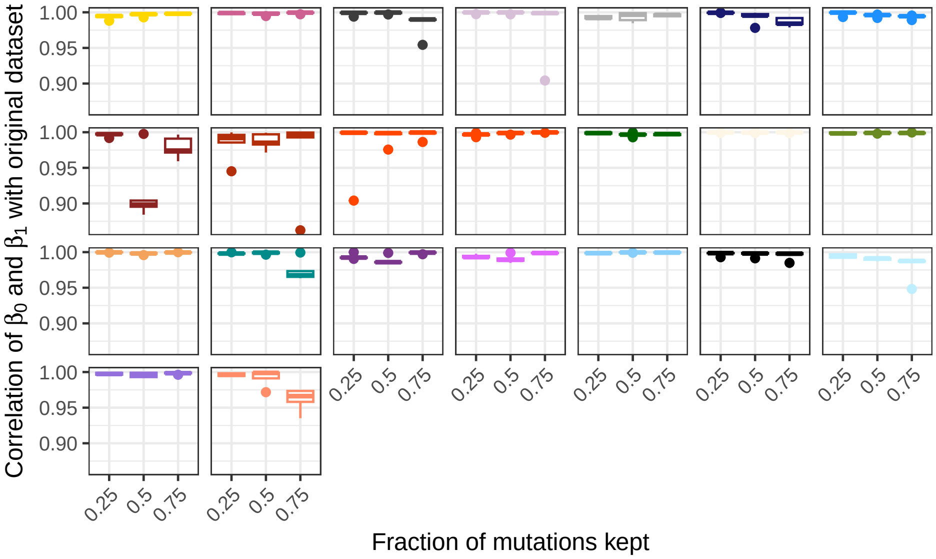

(a) Correlation of $\widehat{\boldsymbol{\beta}_0}$ and $\widehat{\boldsymbol{\beta}}_1$ in each of the three scenarios where split signatures are included in the dataset, compared to the case where the original signature is used.

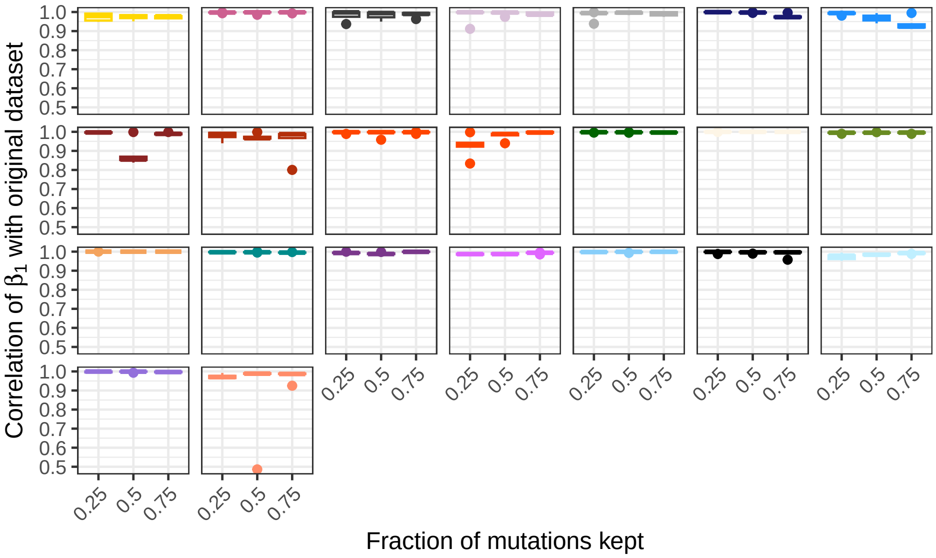

(b) Equivalent of Fig S23a, with correlations computed only among $\widehat{\boldsymbol{\beta}}_1$.

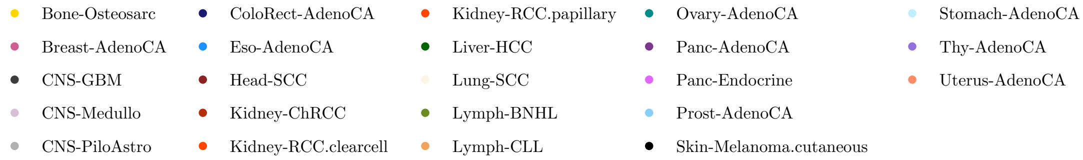

Figure S23: Results for simulation D1D, where signatures selected form the PCAWG datasets are split into two other signatures. Correlation between $\widehat{\boldsymbol{\beta}}_0$ and $\widehat{\boldsymbol{\beta}}_1$ from the original dataset and $\widehat{\boldsymbol{\beta}}_0$ and $\widehat{\boldsymbol{\beta}}_1$ of the modified dataset.

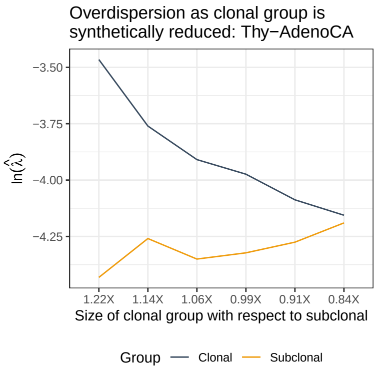

(a) Simulation D2A for Thy-AdenoCA

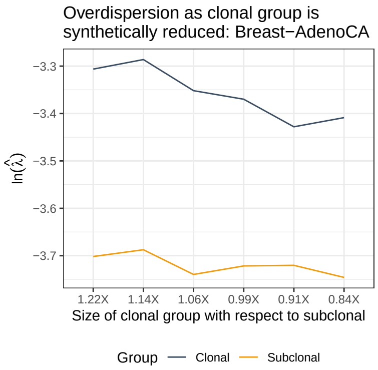

(b) Simulation D2A for Breast-AdenoCA

Figure S24: Results for simulation D2A to show that the lower precision in the subclonal group is not merely a reflection of the lower number of mutations in this group. On the y axis, $\boldsymbol{\lambda}$ values for datasets in which the number of clonal mutations in reduced, from left to right, and for two cancer types. The x axis corresponds to the fold-change of the number of clonal to subclonal mutations, with $1\times$ being the same number, $> 1\times$ indicating a higher fraction of clonal mutations (as in the original datasets) and $< 1\times$ indicating a higher fraction of subclonal mutations.

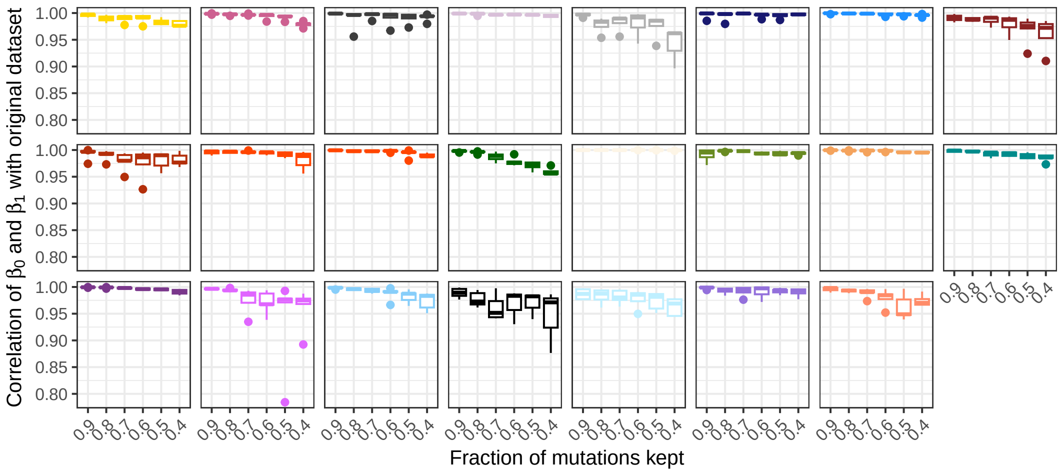

(a) Results for $\widehat{\boldsymbol{\beta}}_0$, $\widehat{\boldsymbol{\beta}}_1$: as the fraction of mutations kept in the analysis is reduced (along the x-axis), the Pearson correlation of estimated $\widehat{\boldsymbol{\beta}}_0$, $\widehat{\boldsymbol{\beta}}_1$ differs more from those estimated using the intact cohort. Note that all correlations are > 0.8.

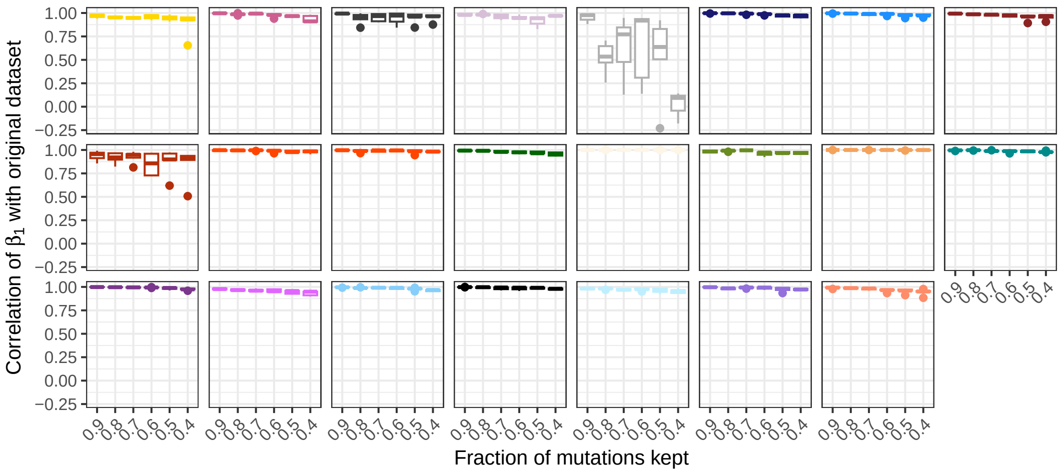

(b) Results for $\widehat{\boldsymbol{\beta}}_1$: as the fraction of mutations kept in the analysis is reduced (along the x-axis), the Pearson correlation of estimated $\widehat{\boldsymbol{\beta}}_1$ differs more from those estimated using the intact cohort. In most cancer types, up to 60% of mutations can be removed prior to signature extraction without impairing the ability to estimate $\widehat{\boldsymbol{\beta}}_1$.

Bone-Osteosarc, ColoRect-AdenoCA, Kidney-RCC.papillary, Ovary-AdenoCA, Stomach-AdenoCA
Breast-AdenoCA, Eso-AdenoCA, Liver-HCC, Panc-AdenoCA, Thy-AdenoCA
CNS-GBM, Head-SCC, Lung-SCC, Panc-Endocrine, Uterus-AdenoCA
CNS-Medullo, Kidney-ChRCC, Lymph-BNHL, Prost-AdenoCA
CNS-PiloAstro, Kidney-RCC.clearcell, Lymph-CLL, Skin-Melanoma.cutaneous

Figure S25: Results for simulation D2B, in which mutations are removed from the dataset, signatures are re-extracted, and `diagREDM` is run.

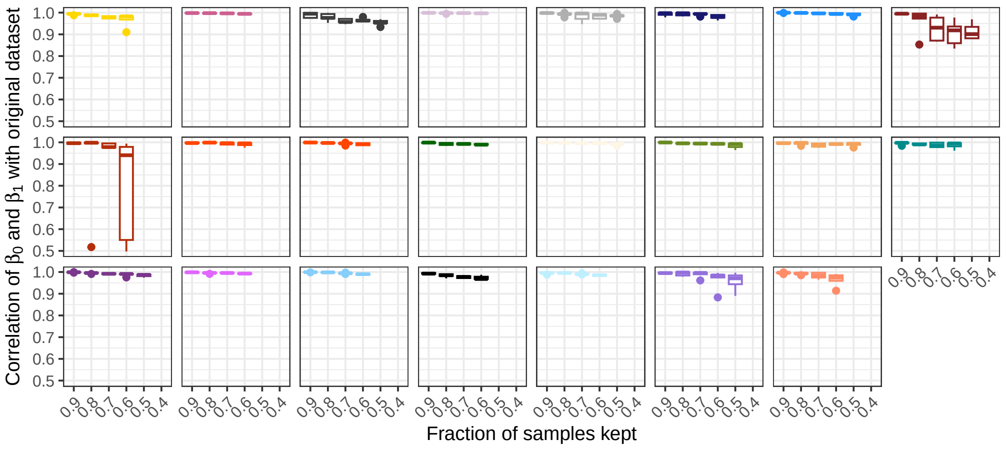

(a) Results for $\widehat{\boldsymbol{\beta}}_0$, $\widehat{\boldsymbol{\beta}}_1$: as the fraction of samples kept in the analysis is reduced (along the x-axis), the Pearson correlation of estimated $\widehat{\boldsymbol{\beta}}_0$, $\widehat{\boldsymbol{\beta}}_1$ differs more from those estimated using the intact cohort. Note that all correlations are > 0.5.

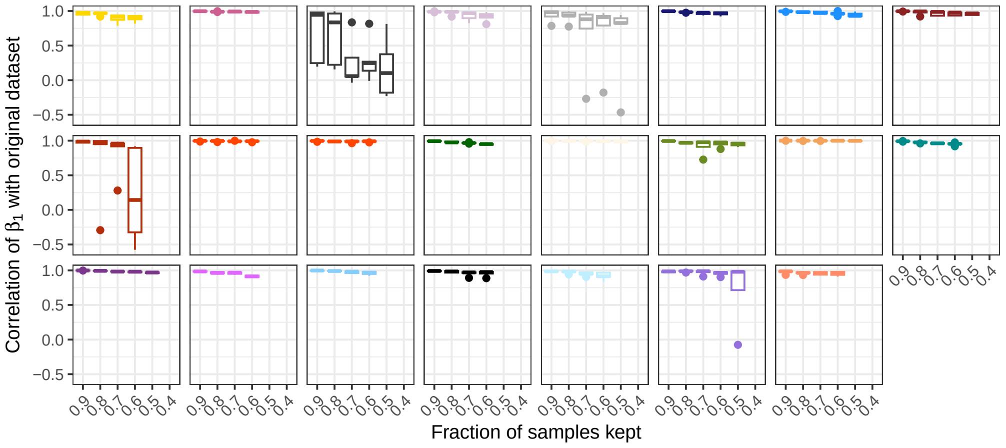

(b) Results for $\widehat{\boldsymbol{\beta}}_1$: as the fraction of samples kept in the analysis is reduced (along the x-axis), the Pearson correlation of estimated$\widehat{\boldsymbol{\beta}}_1$ differs more from those estimated using the intact cohort.

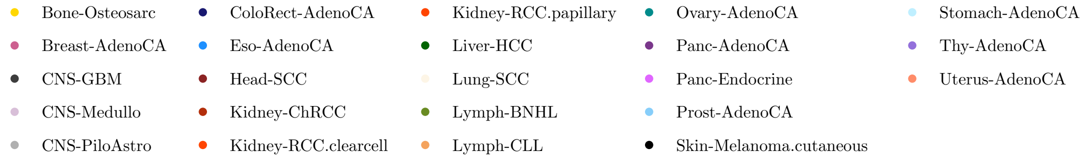

Figure S26: Results for simulation D2C: assessing the recovery of $\boldsymbol{\beta}$ as more samples are removed from the input datasets.

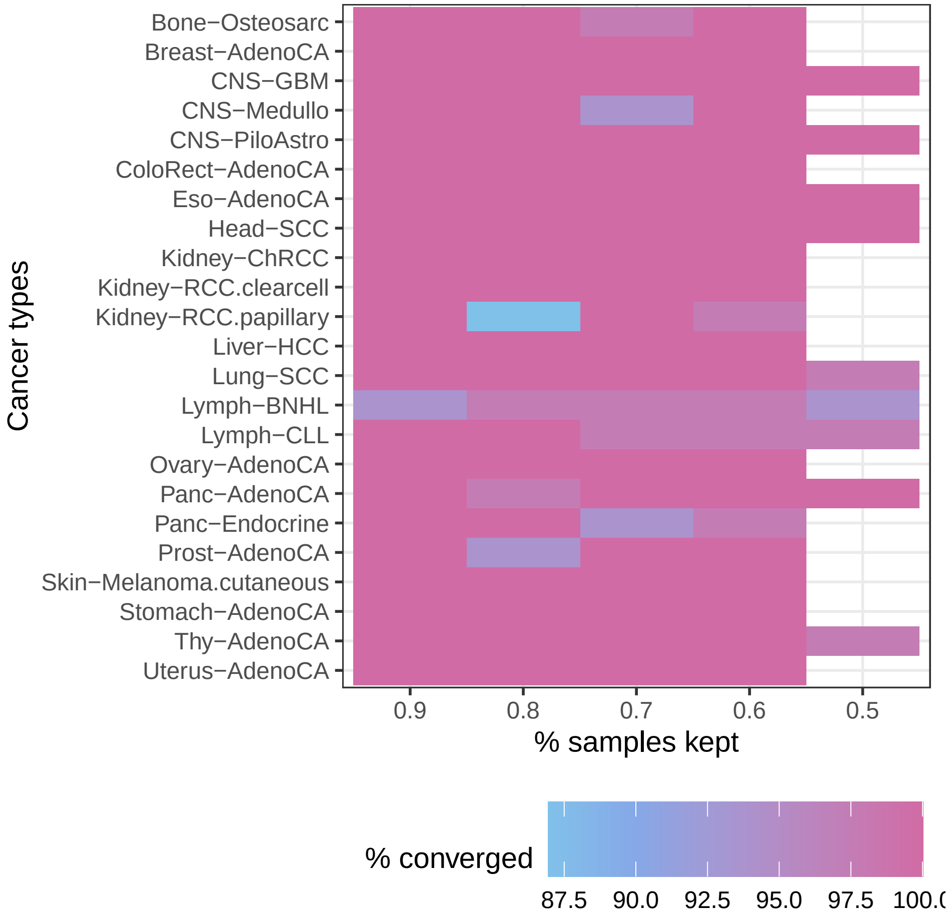

Figure S27: Results for simulation D2C: fraction of samples that have converged, across replicates, for each combination of cancer type and fraction of samples used. Transparent cells correspond to cases where no replicates converged, where large fractions of samples were removed. Pink colours indicate convergence of all replicates.

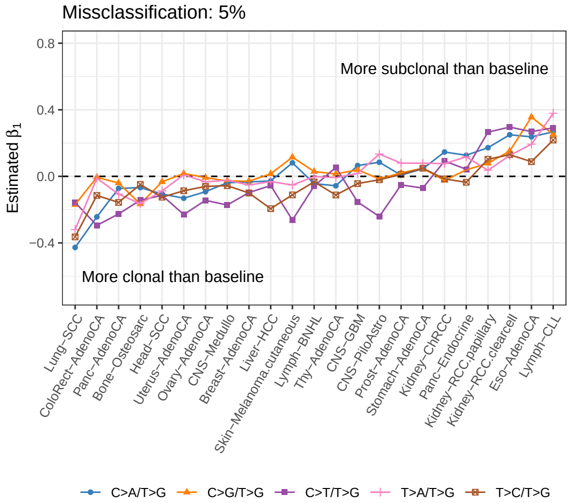

(a) Equivalent of Fig 4 when 5% of mutations are misclassified.

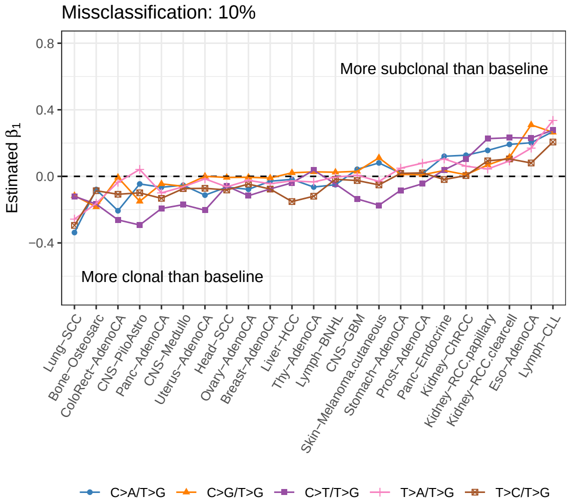

(b) Equivalent of Fig 4 when 10% of mutations are misclassified.

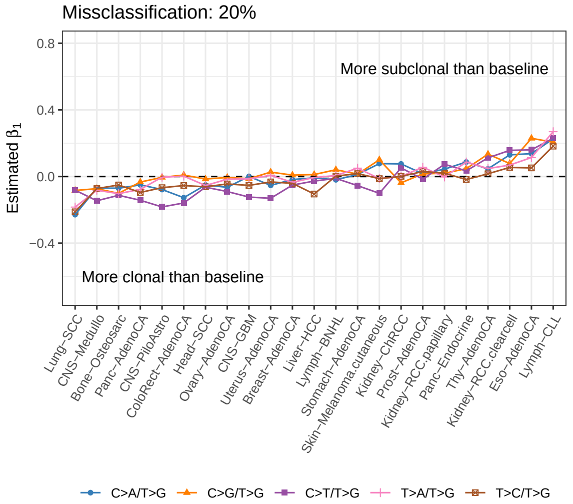

(c) Equivalent of Fig 4 when 20% of mutations are misclassified.

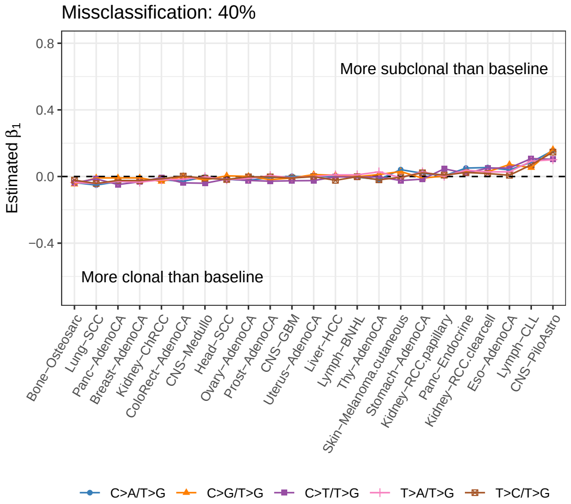

(d) Equivalent of Fig 4 when 40% of mutations are misclassified.

Figure S28: Results for simulation D3. Plots equivalent to Fig 4 when several percentages of mutations are misclassified.

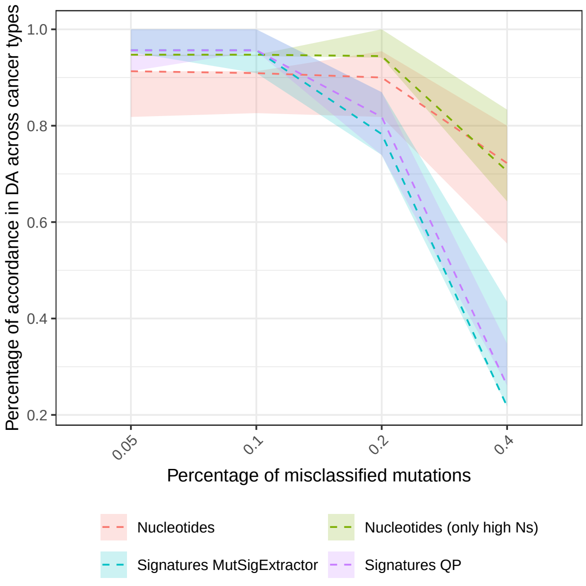

Figure S29: Percentages of runs (across cancer types and replicates) where there is a disagreement on differential abundance with the results shown in the main results. This comparison is done for mutational signatures (where two methods are used for signature re-extraction following mutation misclassification, quadratic programming and MutSigExtractor) and for nucleotides. The disagreement in differential abundance for nucleotides can be attributed to five cancer types (Bone-Osteosarc, Lymph-BNHL, Head-SCC, Kidney=ChRCC, Thy-AdenoCA), as it can be seen when they are removed (in green). The dashed line represents the median across cancer types and the ribbon the range from the minimum to the maximum percentage of accordance.

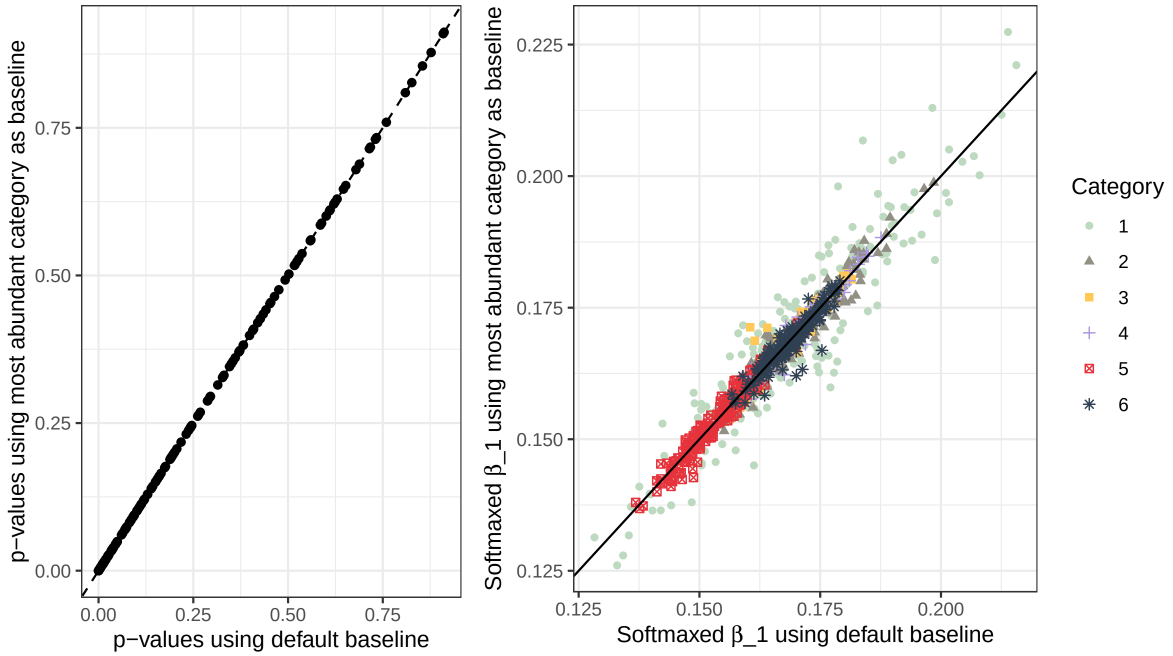

Figure S30: Left: comparison of p-values for datasets in which the baseline signature for ALR differs: along the x-axis, the last signature is used as baseline (as default) and this signature is made differentially abundant; along the y-axis, the signature of highest abundance is chosen. The two sets of p-values align perfectly. Right: comparison of softmaxed-$\widehat{\boldsymbol{\beta}}_1$ for the same datasets – note that without the softmax the results cannot be compared. There is a very good correlation between the two sets of $\widehat{\beta_1}$ across all signatures (each in a different colour).

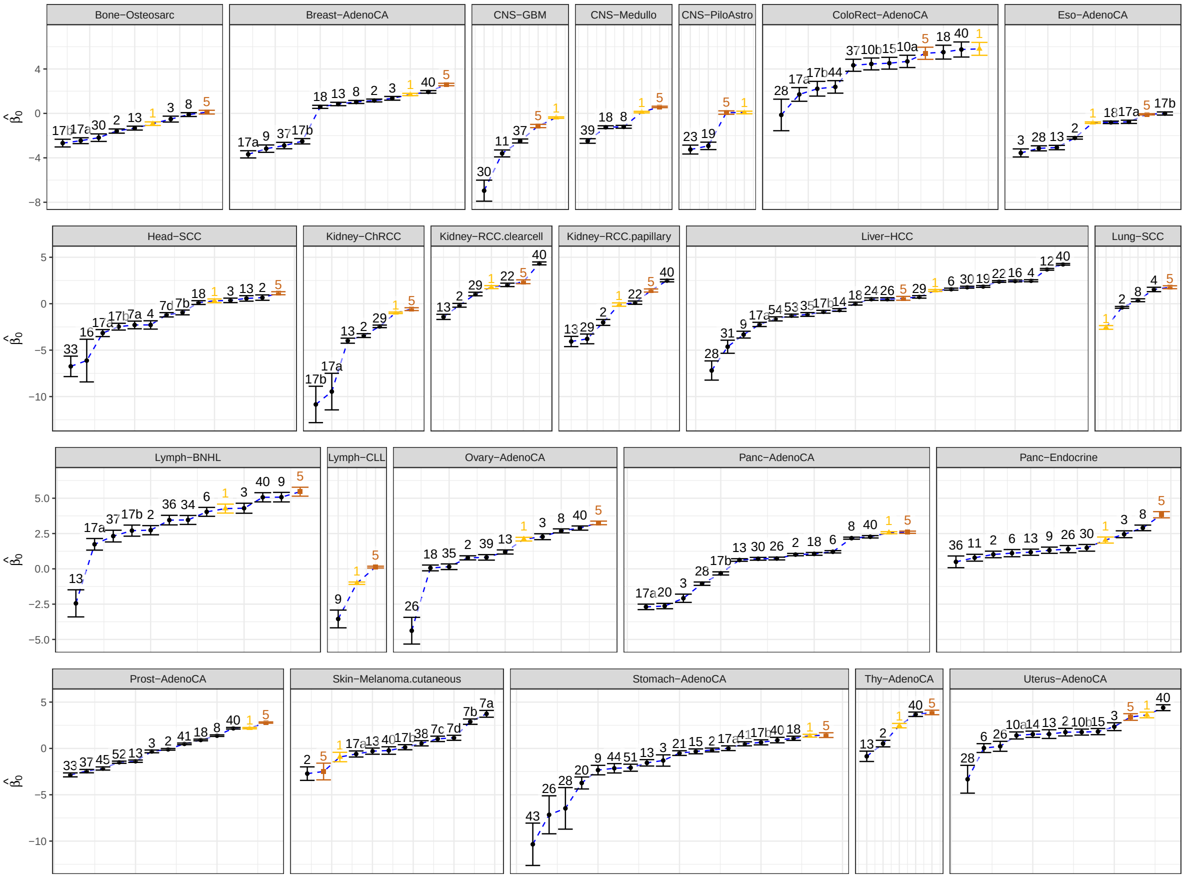

Figure S31: All $\widehat{\boldsymbol{\beta}_0}$ across cancer types. Each coefficient is labelled with the signature in the numerator of the log-ratio it represents. Lower coefficients indicate lower abundance in the clonal group, and higher coefficients indicate higher abundance in the clonal group. If SBS1 or SBS5 are the signature in the numerator, their $\widehat{\boldsymbol{\beta}_0}$ has been coloured in yellow and red respectively. Note that these two coefficients are nearly always adjacent, or at least of similar value, indicating they are present in similar abundances in the clonal group. SBS1 and SBS5 tend to be found at the upper end of the plot, indicating that many signatures have lower abundances than SBS1 or SBS5 in the clonal group.

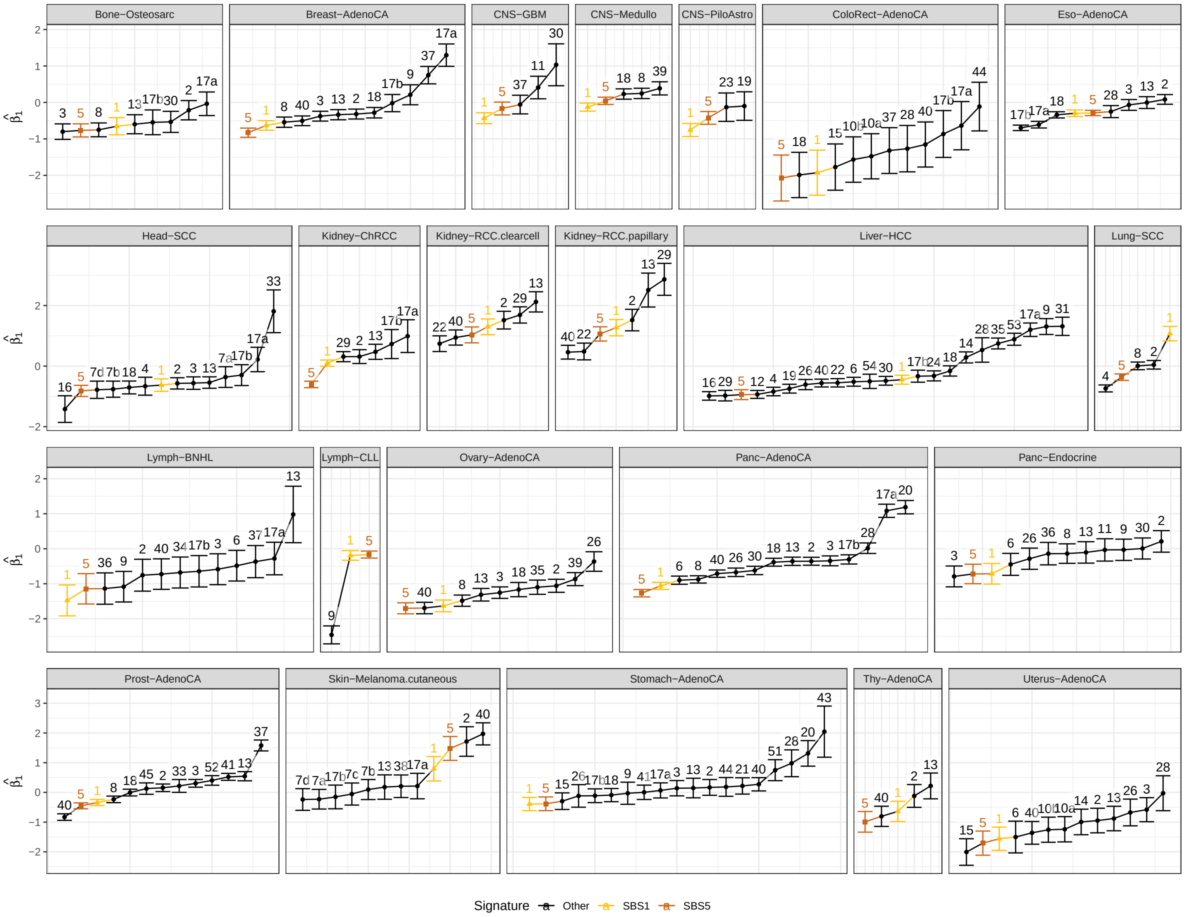

Figure S32: All $\widehat{\boldsymbol{\beta}}_1$ across cancer types. Each coefficient is labelled with the signature in the numerator of the log-ratio it represents. Lower coefficients indicate higher clonality, and higher coefficients indicate higher subclonality. If SBS1 or SBS5 are the signature in the numerator, their $\widehat{\boldsymbol{\beta}}_1$ has been coloured in yellow and red respectively. Note that these two coefficients are nearly always adjacent, or at least of similar value, indicating that their coefficients for differential abundance are very similar. They are generally found in the lower end of the plots, indicating that most signatures are more subclonal than SBS1 or SBS5.

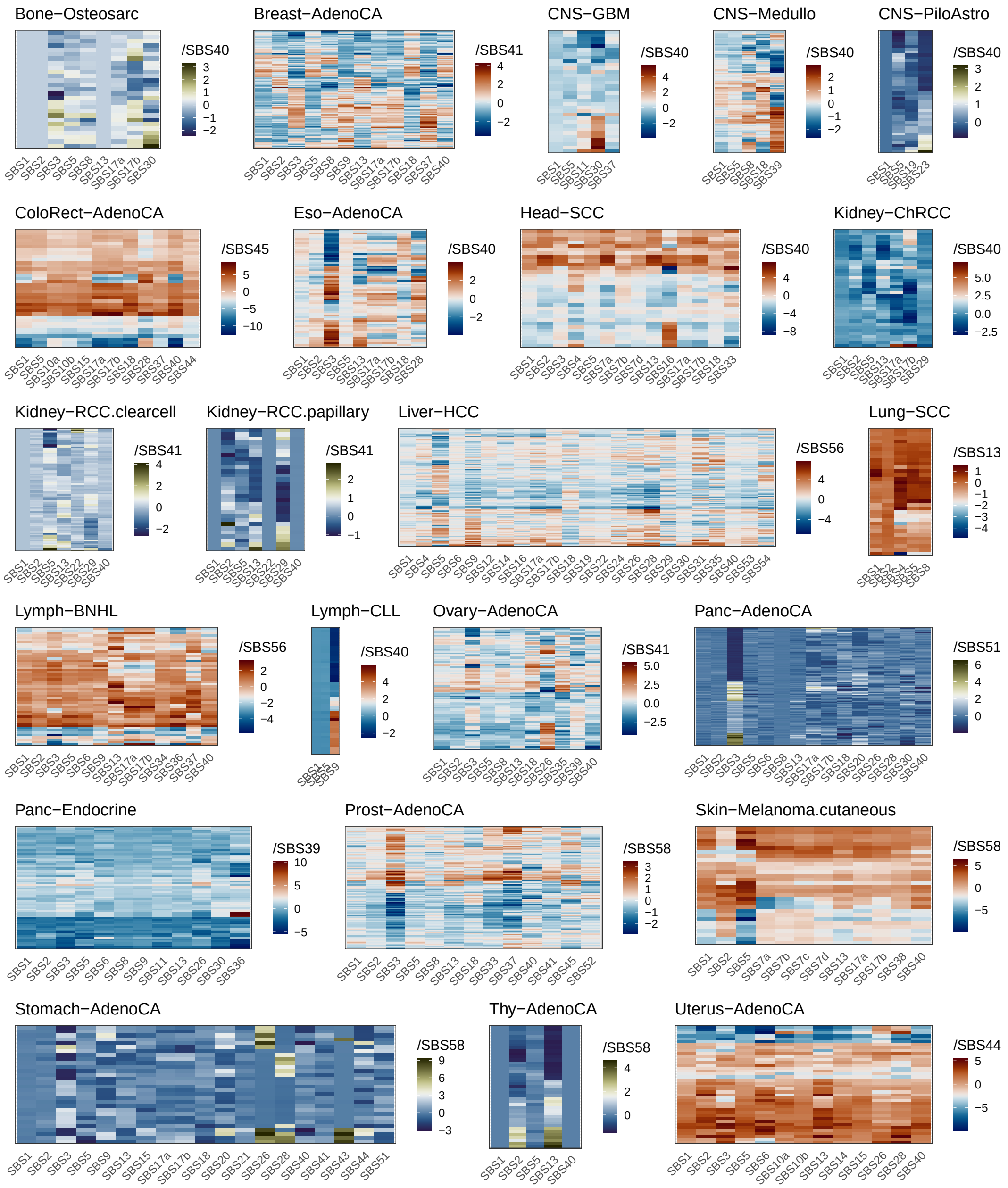

Figure S33: Matrices of the estimated values for the random intercepts of each patient, in which patients are in the rows and coefficients are in the columns. These values are taken prefereably from the results of `fullREDM`, if `fullREDM` has converged in the relevant cancer type, and are taken from the results of `diagREDM` otherwise. As random effects are modelled with a multivariate normal distribution with a mean of $\mathbf{0_K}$, the values are centered around 0. These patient-specific random intercepts indicate the signature abundance with respect to the average signature abundance across all patients in the cancer type cohort (described by $\boldsymbol{\beta}_0$). Values that are high (in red) indicate log-ratios of signatures which are higher in the patient than in the full cancer type cohort. A negative value (in blue) indicates that this log-ratio is lower in the patient than in the full cancer type cohort. The baseline signature is indicated in the legend.

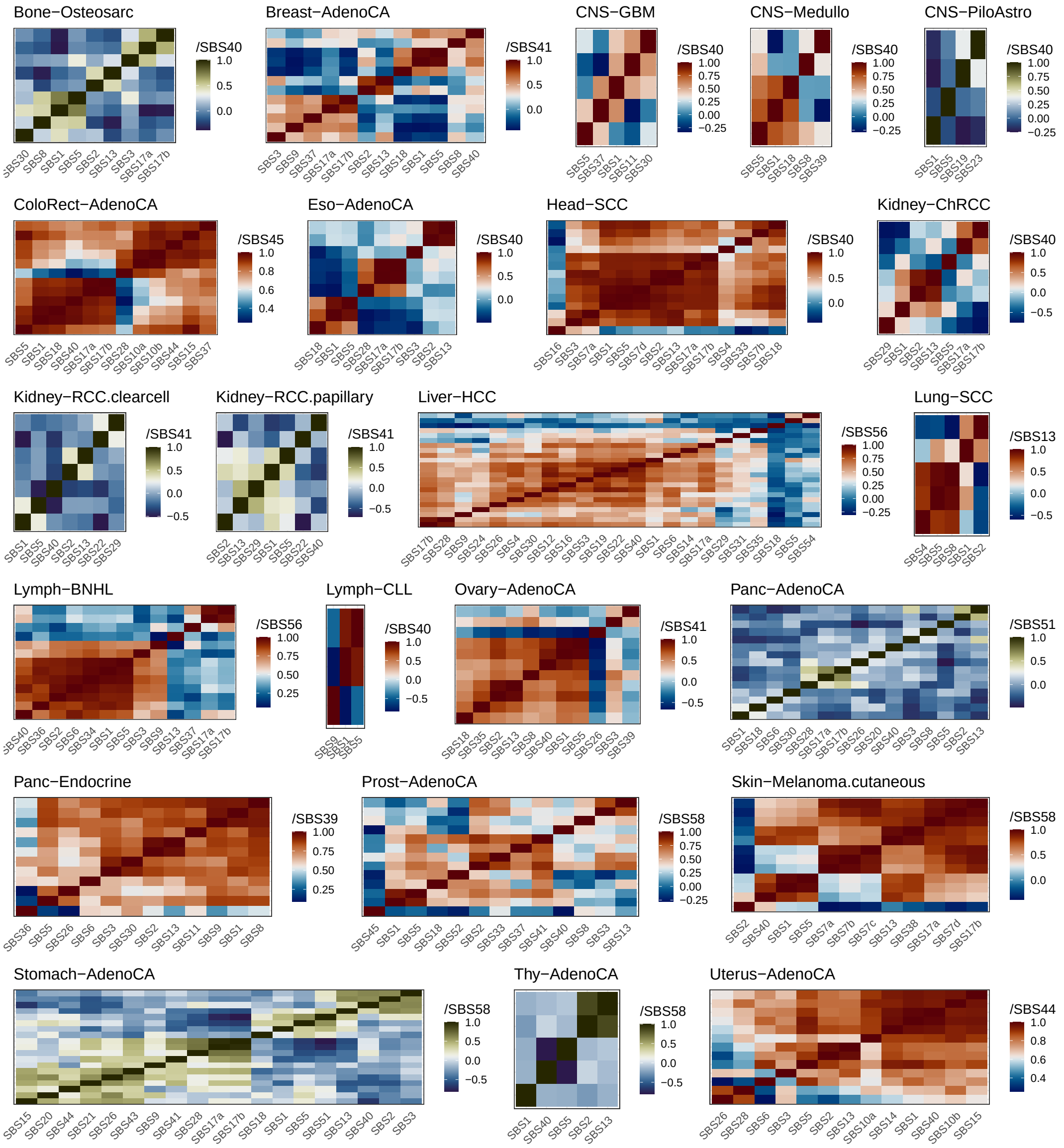

Figure S34: Estimated correlation matrix of patient random intercepts. These values are taken from the estimated parameters of `fullREDM`, if converged, and are the correlation values computed from the random intercepts of `diagREDM` otherwise. Red colours indicate higher correlations.

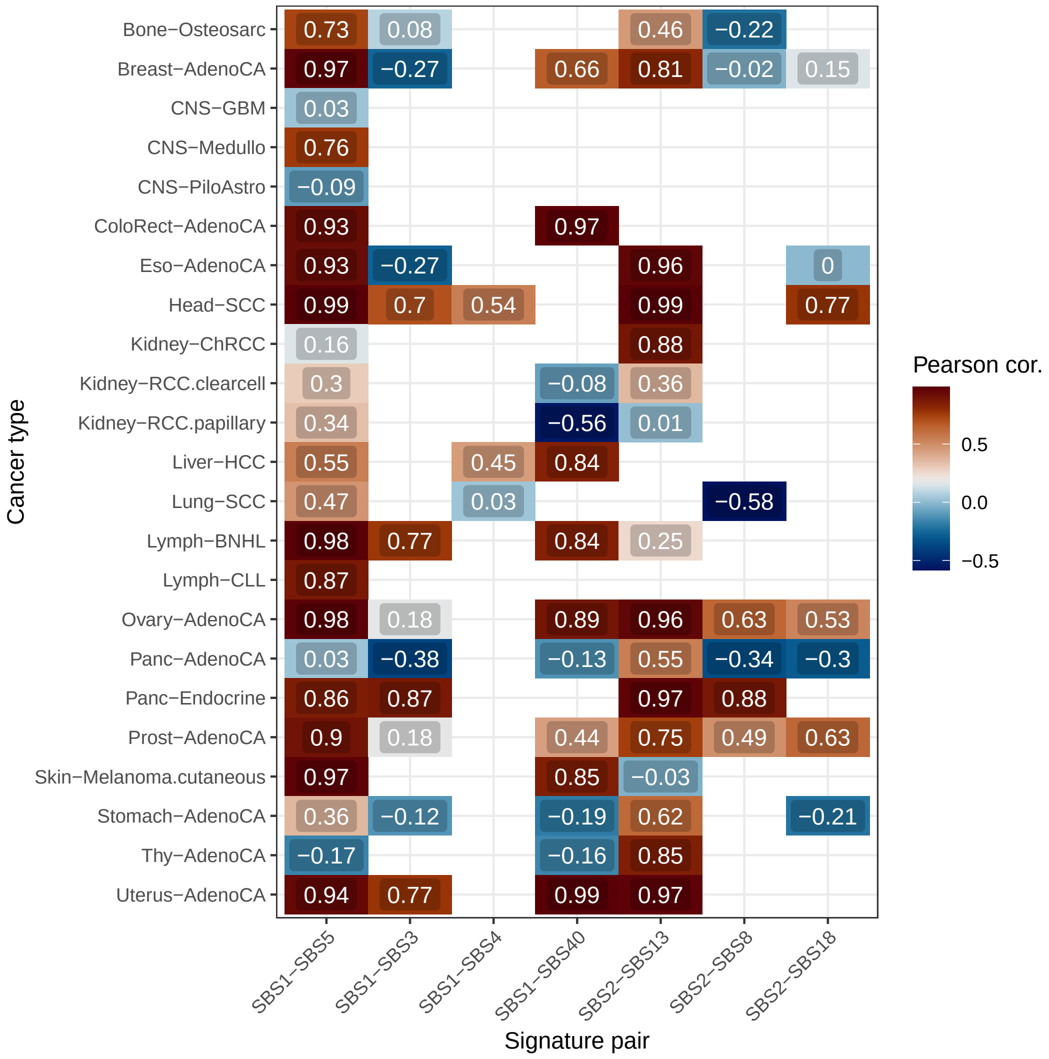

Figure S35: Estimated correlations of patient random intercepts, for selected pairs of signatures, i.e. a small subset of the data in Fig S34.

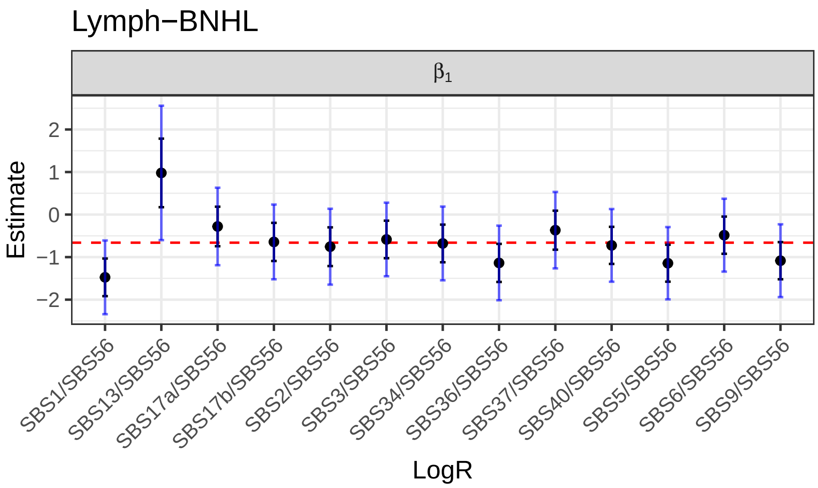

Figure S36: Plot to exemplify the minimal perturbation framework. On the y axis, values of $\boldsymbol{\beta}_1$ for the Lymph-BNHL cohort, with the line of "zero perturbation" in red. The confidence interval (in blue) for signature SBS13 is above this line, and therefore the minimal perturbation framework categorises it as a signature that increases from clonal to subclonal stages.

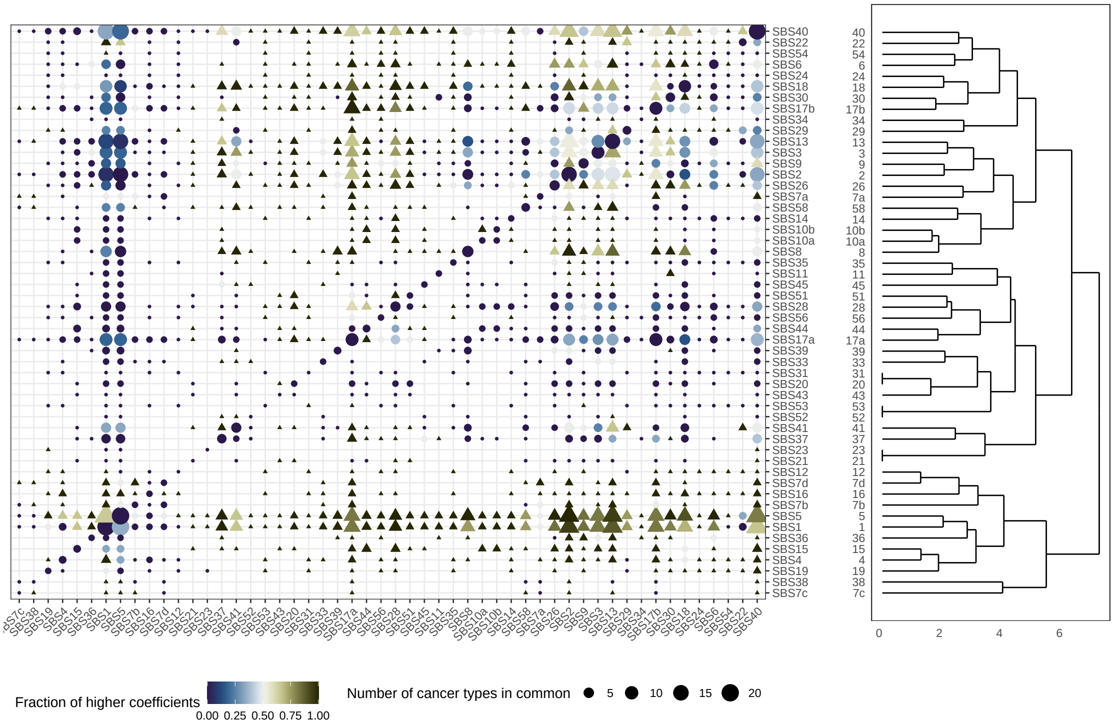

Figure S37: Fraction of cancer types in which the $\widehat{\boldsymbol{\beta}}_1$ of any signature (in the x axis) are higher than those of another signature, for every other signature along the y axis. The vertical clustering is done on Euclidean distance using complete linkage. The size of the dots indicates the number of cancer types in which both signatures are active. The elements in the diagonal have a shared value of 0, and their size indicates the number of cancer types in which the corresponding signature is active. Analysed row-wise, a signature with many blue entries corresponds to a signature which has, consistently, the highest softmaxed-$\widehat{\boldsymbol{\beta}}_1$ across cancer types, suggesting signatures that preferentially create subclonal mutations, or the mutation rate of which increases in subclonal stages of tumour development. On the other extreme, green rows indicate signatures with the lowest softmaxed-$\widehat{\boldsymbol{\beta}}_1$, most likely indicating signatures which decrease in abundance over the course of tumour development, or the mutation rate of which remains constant. Signatures clustered according to the dendrogram on the right of the plot are suggested to share temporal dynamics.

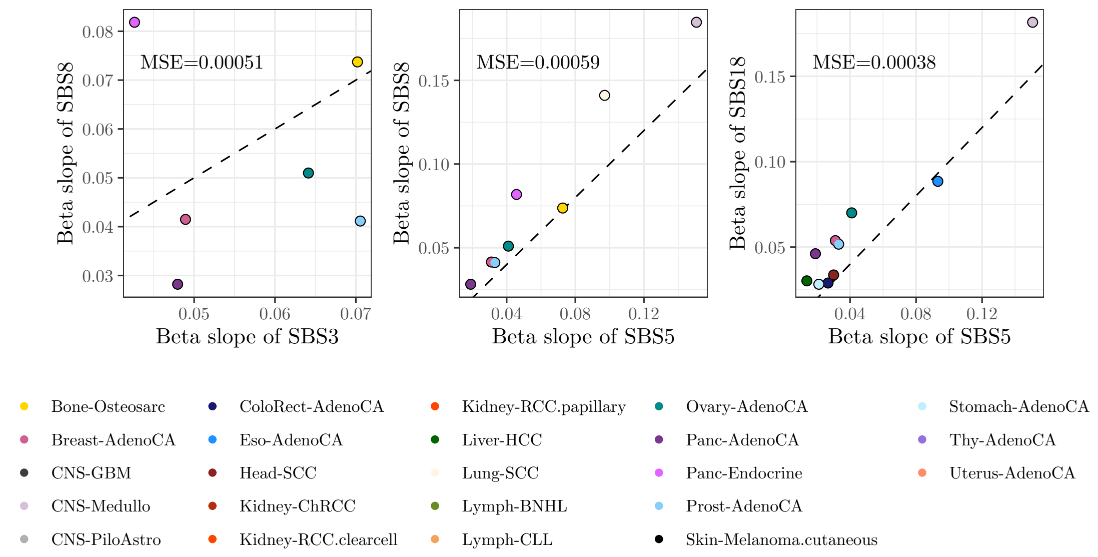

Figure S38: Plots additional to those of Fig 6, with selected pairs of signatures of interest, showing softmax-transformed $\widehat{\boldsymbol{\beta}}_1$ for each cancer type.

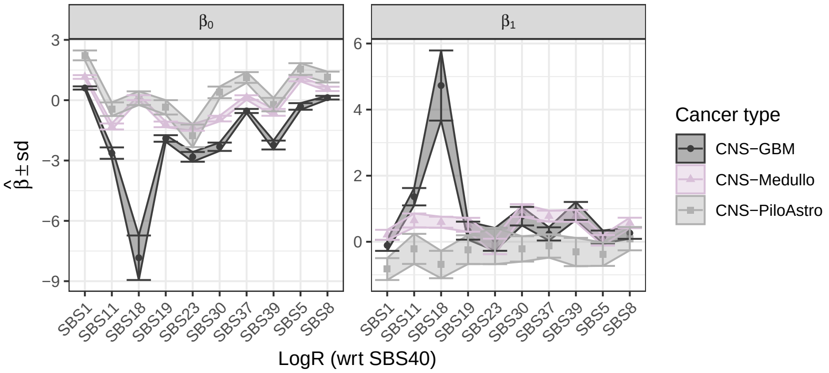

(a) CNS samples

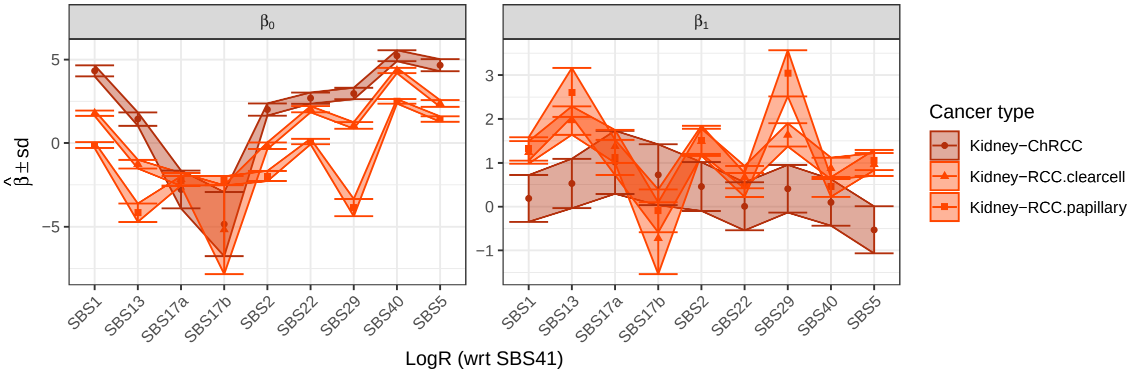

(b) Kidney samples

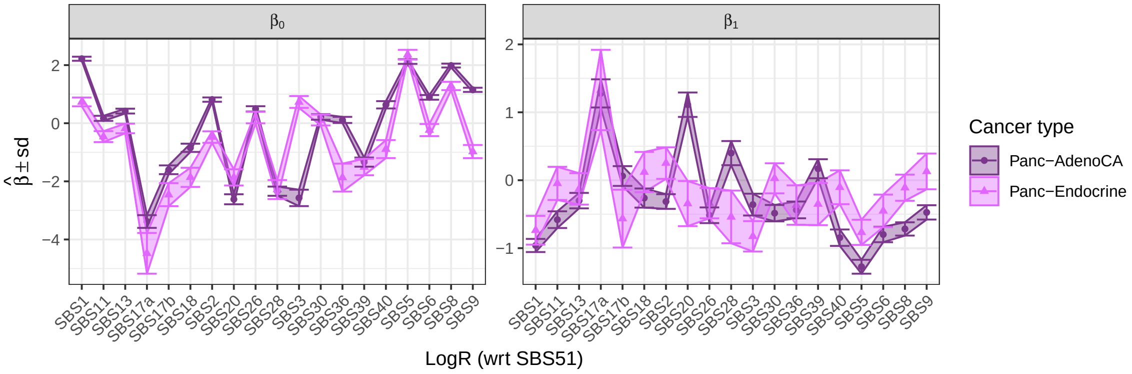

(c) Pancreatic samples

Figure S39: $\widehat{\boldsymbol{\beta}}_0$ and $\widehat{\boldsymbol{\beta}}_1$ for cancer types of selected tissues. These values are derived once signatures are re-extracted using all active signatures in each tissue.

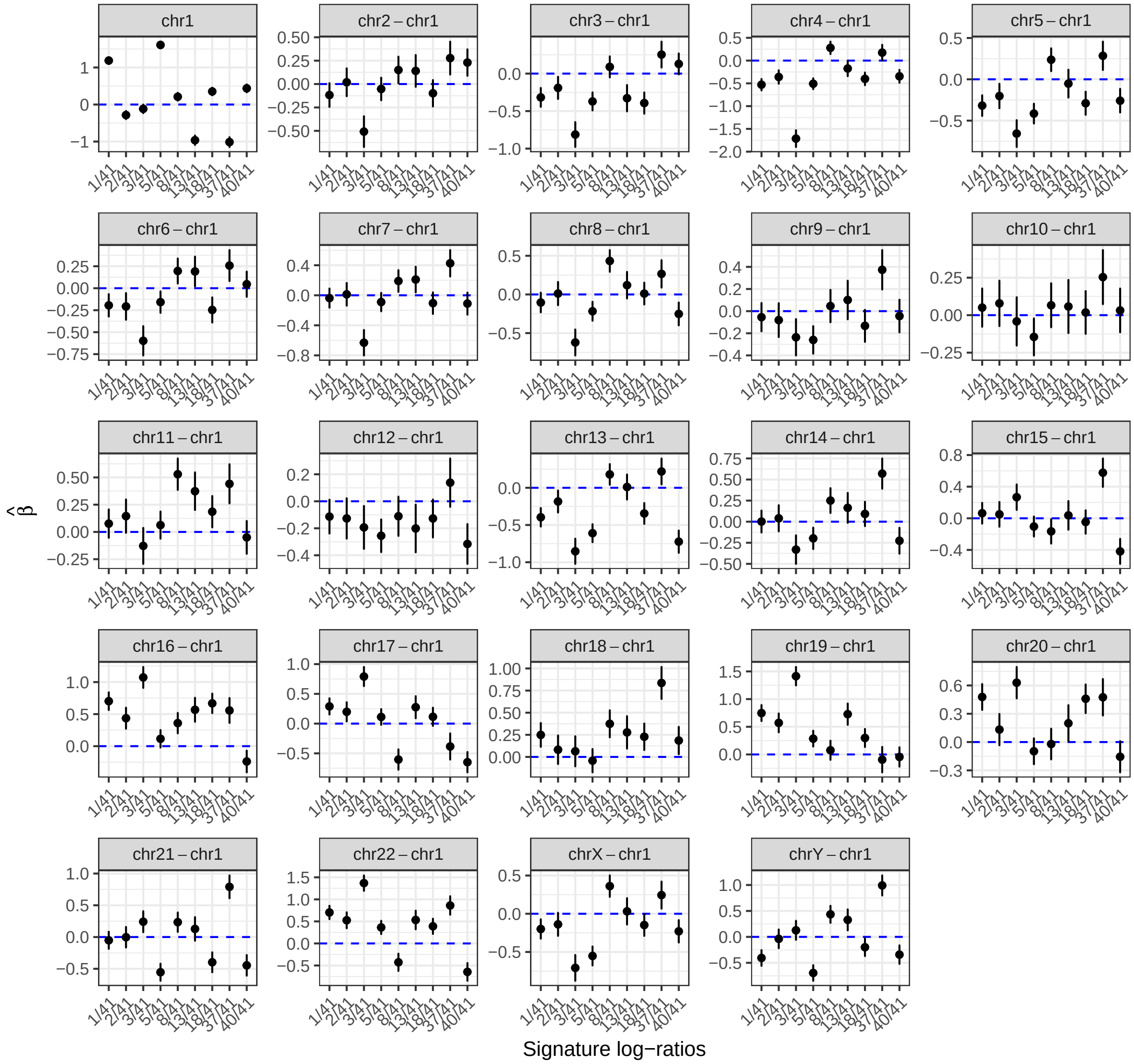

Figure S40: All $\widehat{\boldsymbol{\beta}}$ for each of the chromosomes. The coefficients for SBS3 show markedly different patterns depending on the chromosome. Some clustering of chromosomes can already be noticed, e.g. the similarity between Chr2 and Chr7.

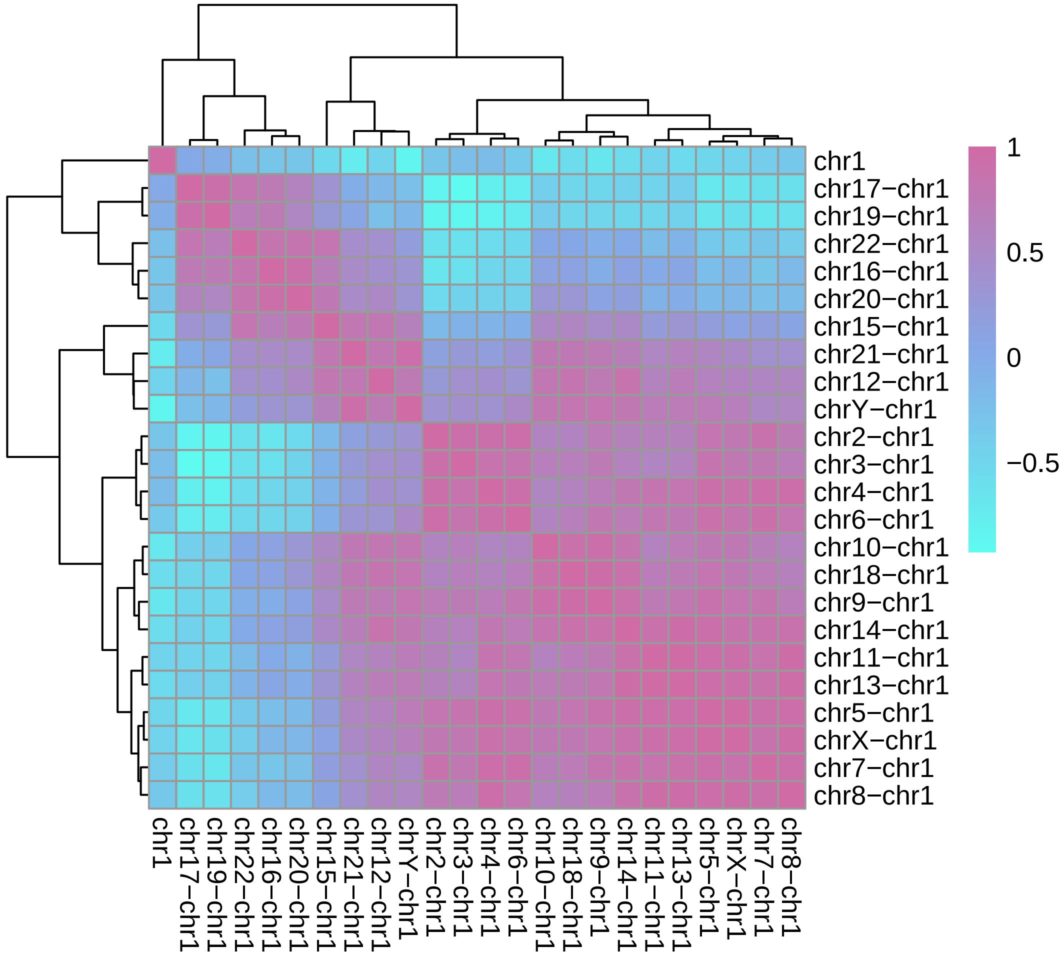

Figure S41: Correlation of $\widehat{\boldsymbol{\beta}}$ for each of the chromosomes, showing some clear clustering. Note that Chr1 should not be analysed in the same way as the rest, as its $\boldsymbol{\beta}_0$ is a reflection of its signature abundance, whereas for the rest of the chromosome it is a reflection of their difference in signature abundance compared to Chr1.

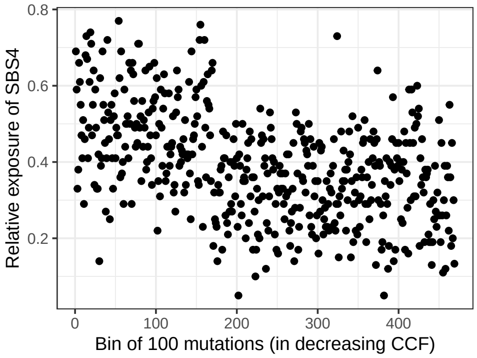

(a) For an example patient, decrease in the relative abundance of SBS4 along bins sorted by descending CCFs (from more clonal to more subclonal).

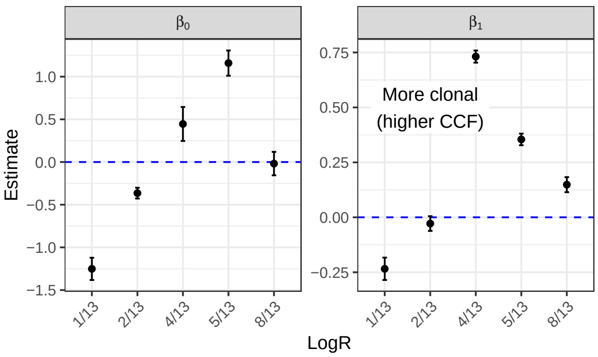

(b) $\widehat{\boldsymbol{\beta}}$ from `diagREDMpatientlambda` using CCF as a covariate. The high coefficient for SBS4 indicates its clonality.

Figure S42: Additional *CompSign* functionality: regression using `diagREDMpatientlambda`, with patient-specific λ parameters and intercepts, and using CCF as a covariate. In the case of LungSCC, several signatures are detected as having positive or negative correlations with CCF.

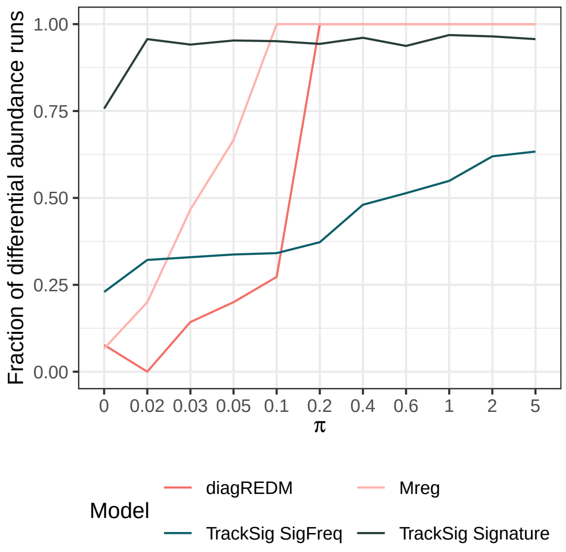

(a) Example for C4C, where TrackSig gives reasonable results for differential abundance but where the cohort-wide models `Mreg` and `diagREDM` perform better, with lower false positive rates and high sensitivity. In TrackSig, there is considered to be differential abundance if there is at least one changepoint.

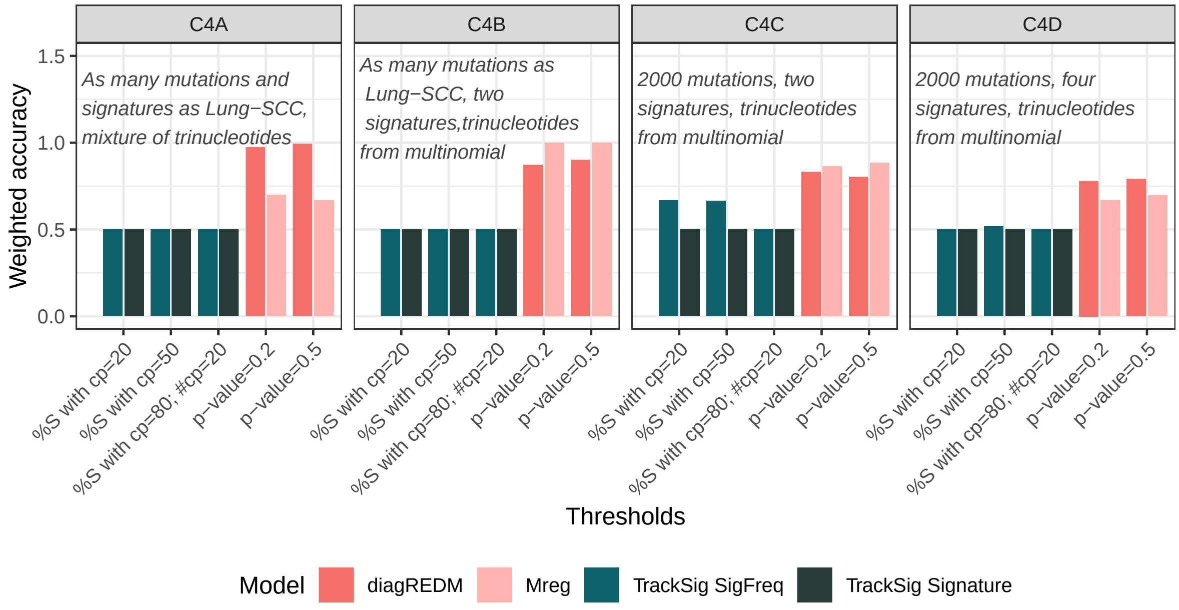

(b) Summary of results in all simulations used for the comparison with TrackSig. TrackSig has comparatively lower accuracy than the cohort-wide models `Mreg` and `diagREDM`.

Figure S43: Additional *CompSign* functionality: comparison between TrackSig (in two versions; SigFreq and Signature), `diagREDM` run on the ground truth groups, and multinomial mixed-effects regression (`Mreg`, `fullREM`) with CCF as a covariate, showing much better accuracies for the latter two models.

Supplementary tables

**Differential abundance in of compositional data**			
–	Mixed-effects Dirichlet-multinomial	Uncorrelated covariance matrix for random effects	[27]
scCODA	Hierarchical Dirichlet-multinomial model with spike-and-slab prior	No random effects in samples	[43]
MGLM	Dirichlet-multinomial with link on the DM parameter α	Simpler model, link on the parameter that determines both mean and variance	[44]
HMP	Two-group Dirichlet-multinomial testing	No random effects	[45]
Aitchison's perturbation	Perturbation test	Not for count data specifically, requires imputation for zeros, no overdispersion	[46]
DirichletReg	Dirichlet regression	Simpler model, no correlations between categories	[47]
–	Logistic-normal regression	Not for count data specifically, requires imputation for zeros, no overdispersion	[46]
–	T-test on raw values	Simpler model, in principle not suitable for compositional data	

Table S1: Methods for determining changes in the mutational spectrum, and general regression methods for compositional data.

Name of model in *CompSign*	Description of model and suggested usage
`FEDMsinglelambda`	Dirichlet-multinomial with no RE and a single $\boldsymbol{\lambda}$ which can be used in a two-group comparison (if we consider the dipersion to be the same in both groups), a multi-group comparison, or any regression setting.
`FE_DM`	Dirichlet-multinomial with no random effects and two $\boldsymbol{\lambda}$, used for the comparison between two groups in which we want to account for group-specific dispersion. Model slightly more complex than `FEDMsinglelambda`.
`diagRE_M`	Multinomial with non-correlated multivariate effects. Simpler model than `diagRE_DM`, as no dispersion is included.
`diagRE_DM_singlelambda`	Dirichlet-multinomial with non-correlated multivariate RE and one λ : equivalent of `diagRE_DM` but with a shared λ. It can be used in a two-group comparison (if we consider the dipersion to be the same in both groups), a multi-group comparison, or any regression setting.
`singleRE_DM`	Dirichlet-multinomial with a single RE intercept and two $\boldsymbol{\lambda}$: simple model in which random effects are not multivariate, but where group-specific dispersion is needed, in a two-group comparison.
`diagRE_DM`	Dirichlet-multinomial with independent RE and two $\boldsymbol{\lambda}$: used most commonly throughout the paper. The data are matched to warrant multivariate RE, but correlations between categories are not explicitly modelled. Faster than `fullRE_DM` with often comparable results for $\boldsymbol{\beta}_1$.
`fullRE_DM_singlelambda`	Dirichlet-multinomial with correlated RE and two $\boldsymbol{\lambda}$: equivalent of `diagRE_DM_singlelambda` that can be used if categories have strong correlations.
`fullRE_M`	Multinomial with correlated RE: assuming no overdispersion, it can be used in any regression setting.
`fullRE_DM`	Dirichlet-multinomial with correlated RE and two $\boldsymbol{\lambda}$: model with multivariate RE with correlations modelled explicitly. As a $(K-1) \times (K-1)$ covariance matrix is estimated, this model is not recommended where the ratio of signatures to samples is high.
`diagREDMpatientlambda`	Dirichlet-multinomial with non-correlated RE and one λ per patient: model more complex than `diagRE_DM` that can be used when there are several observations per patient and we wish to include a patient-specific dispersion parameter.
`fullRE_DM_patientlambda`	Dirichlet-multinomial with correlated RE and one λ per patient: equivalent of `diagREDMpatientlambda`, but modelling correlations between categories.

Table S2: Variations of the model implemented in the *CompSign* package, in ascending order of complexity. RE refers to random effects.

	Fraction of zeros	Max. fraction of zeros in signatures
Bone-Osteosarc	0.12	0.37
Breast-AdenoCA	0.29	0.77
CNS-GBM	0.23	0.79
CNS-Medullo	0.13	0.44
CNS-PiloAstro	0.28	0.65
ColoRect-AdenoCA	0.27	0.82
Eso-AdenoCA	0.10	0.42
Head-SCC	0.27	0.81
Kidney-ChRCC	0.32	0.89
Kidney-RCC.clearcell	0.21	0.58
Kidney-RCC.papillary	0.22	0.67
Liver-HCC	0.39	0.93
Lung-SCC	0.03	0.13
Lymph-BNHL	0.22	0.88
Lymph-CLL	0.15	0.58
Ovary-AdenoCA	0.18	0.76
Panc-AdenoCA	0.19	0.61
Panc-Endocrine	0.24	0.59
Prost-AdenoCA	0.26	0.76
Skin-Melanoma.cutaneous	0.26	0.62
Stomach-AdenoCA	0.30	0.88
Thy-AdenoCA	0.22	0.52
Uterus-AdenoCA	0.31	0.79

Table S3: Fraction of zero exposures, and fraction of zero exposures in the signature with most zeros, in each PCAWG dataset.

Model	Significance level	Accuracy	Balanced accuracy	FPR	TPR	N	T	Dataset
HiLDA	0.05	0.75	0.50		0.00	100	50	C1
TCSM	0.05	0.25	0.50	0.25	1.00	100	50	C1
diagREDM	0.05	0.47	0.36	0.11	0.15	100	50	C1
HiLDA	0.05	–				100	1773	C1
TCSM	0.05	0.25	0.50	0.25	1.00	100	1773	C1
diagREDM	0.05	0.06	0.08	0.03	0.10	100	1773	C1
HiLDA	0.05	0.75	0.50		0.00	20	50	C2
TCSM	0.05	0.25	0.50	0.25	1.00	20	50	C2
diagREDM	0.05	0.73	0.54	0.40	0.15	20	50	C2
HiLDA	0.05	0.75	0.50		0.00	100	50	C2
TCSM	0.05	0.25	0.49	0.25	0.97	100	50	C2
diagREDM	0.05	0.61	0.45	0.17	0.15	100	50	C2
HiLDA	0.05	0.75	0.50		0.00	20	50	C3
TCSM	0.05	0.26	0.50	0.25	1.00	20	50	C3
diagREDM	0.05	0.64	0.45	0.13	0.07	20	50	C3
HiLDA	0.05	0.75	0.50		0.00	100	50	C3
TCSM	0.05	0.26	0.50	0.25	1.00	100	50	C3
diagREDM	0.05	0.56	0.43	0.16	0.17	100	50	C3
HiLDA	0.05	–				100	Obs	C3
TCSM	0.05	0.27	0.50	0.25	0.95	100	Obs	C3
diagREDM	0.05	0.48	0.33	0.02	0.03	100	Obs	C3
HiLDA	0.05	–				150	Obs	C3
TCSM	0.05	0.28	0.51	0.25	0.97	150	Obs	C3
diagREDM	0.05	0.45	0.31	0.02	0.03	150	Obs	C3

Table S4: Results for simulations C1-3: performance of each model in each dataset, as parameters vary. In three simulations where the number of simulated mutations is relatively high we have not been able to get results from HiLDA.

Model	Dataset	N	T	Mean (s)	sd (s)	Min (s)	Max (s)
HiLDA	C1	100	50	431.39	15.49	412.30	554.90
TCSM	C1	100	50	4.31	0.51	3.67	8.60
diagREDM	C1	100	50	3.78	0.27	2.98	4.84
TCSM	C1	100	1773	5.08	0.54	4.25	6.54
diagREDM	C1	100	1773	3.61	0.20	3.16	4.48
HiLDA	C2	20	50	18.42	0.87	16.86	20.95
TCSM	C2	20	50	3.60	0.20	3.27	4.25
diagREDM	C2	20	50	0.33	0.03	0.27	0.58
HiLDA	C2	100	50	298.20	15.81	279.19	363.81
TCSM	C2	100	50	3.95	0.26	3.59	5.93
diagREDM	C2	100	50	1.50	0.12	1.21	1.89
HiLDA	C3	20	50	17.89	0.67	16.65	20.17
TCSM	C3	20	50	3.72	0.29	3.30	5.15
diagREDM	C3	20	50	0.34	0.03	0.29	0.58
HiLDA	C3	100	50	291.20	10.14	277.81	347.62
TCSM	C3	100	50	4.06	0.42	3.60	8.25
diagREDM	C3	100	50	1.54	0.12	1.33	1.97
TCSM	C3	100	Obs	4.45	0.19	4.12	5.77
diagREDM	C3	100	Obs	1.22	0.13	0.05	1.47
TCSM	C3	150	Obs	4.63	0.21	4.29	6.30
diagREDM	C3	150	Obs	1.95	0.16	1.64	2.78

Table S5: Results for simulations C1-3: runtime, in seconds, for each of the models, in which `diagREDM` is shown to be the fastest model.

Cancer type	Active signatures
Bone-Osteosarc	SBS1*, SBS2, SBS3, SBS5*, SBS8, SBS13, SBS17a, SBS17b, SBS30, SBS40
Breast-AdenoCA	SBS1*, SBS2, SBS3, SBS5*, SBS8, SBS9, SBS13, SBS17a, SBS17b, SBS18, SBS37, SBS40, SBS41
CNS-GBM	SBS1*, SBS5*, SBS11*, SBS30, SBS37, SBS40
CNS-Medullo	SBS1*, SBS5*, SBS8, SBS18, SBS39, SBS40
CNS-PiloAstro	SBS1*, SBS5*, SBS19, SBS23, SBS40
ColoRect-AdenoCA	SBS1*, SBS5*, SBS10a, SBS10b, SBS15, SBS17a, SBS17b, SBS18, SBS28, SBS37, SBS40, SBS44, SBS45*
Eso-AdenoCA	SBS1*, SBS2, SBS3, SBS5*, SBS13, SBS17a, SBS17b, SBS18, SBS28, SBS40
Head-SCC	SBS1*, SBS2, SBS3, SBS4*, SBS5*, SBS7a*, SBS7b*, SBS7d*, SBS13, SBS16, SBS17a, SBS17b, SBS18, SBS33, SBS40
Kidney-ChRCC	SBS1*, SBS2, SBS5*, SBS13, SBS17a, SBS17b, SBS29*, SBS40
Kidney-RCC.clearcell	SBS1*, SBS2, SBS5*, SBS13, SBS22, SBS29*, SBS40, SBS41
Kidney-RCC.papillary	SBS1*, SBS2, SBS5*, SBS13, SBS22, SBS29*, SBS40, SBS41
Liver-HCC	SBS1*, SBS4*, SBS5*, SBS6, SBS9, SBS12, SBS14, SBS16, SBS17a, SBS17b, SBS18, SBS19, SBS22, SBS24, SBS26, SBS28, SBS29*, SBS30, SBS31*, SBS35*, SBS40, SBS53, SBS54*, SBS56*
Lung-SCC	SBS1*, SBS2, SBS4*, SBS5*, SBS8, SBS13
Lymph-BNHL	SBS1*, SBS2, SBS3, SBS5*, SBS6, SBS9, SBS13, SBS17a, SBS17b, SBS34, SBS36, SBS37, SBS40, SBS56*
Lymph-CLL	SBS1*, SBS5*, SBS9, SBS40
Ovary-AdenoCA	SBS1*, SBS2, SBS3, SBS5*, SBS8, SBS13, SBS18, SBS26, SBS35*, SBS39, SBS40, SBS41
Panc-AdenoCA	SBS1*, SBS2, SBS3, SBS5*, SBS6, SBS8, SBS13, SBS17a, SBS17b, SBS18, SBS20, SBS26, SBS28, SBS30, SBS40, SBS51*
Panc-Endocrine	SBS1*, SBS2, SBS3, SBS5*, SBS6, SBS8, SBS9, SBS11*, SBS13, SBS26, SBS30, SBS36, SBS39
Prost-AdenoCA	SBS1*, SBS2, SBS3, SBS5*, SBS8, SBS13, SBS18, SBS33, SBS37, SBS40, SBS41, SBS45*, SBS52*, SBS58*
Skin-Melanoma.cutaneous	SBS1*, SBS2, SBS5*, SBS7a*, SBS7b*, SBS7c*, SBS7d*, SBS13, SBS17a, SBS17b, SBS38, SBS40, SBS58*
Stomach-AdenoCA	SBS1*, SBS2, SBS3, SBS5*, SBS9, SBS13, SBS15, SBS17a, SBS17b, SBS18, SBS20, SBS21, SBS26, SBS28, SBS40, SBS41, SBS43*, SBS44, SBS51*, SBS58*
Thy-AdenoCA	SBS1*, SBS2, SBS5*, SBS13, SBS40, SBS58*
Uterus-AdenoCA	SBS1*, SBS2, SBS3, SBS5*, SBS6, SBS10a, SBS10b, SBS13, SBS14, SBS15, SBS26, SBS28, SBS40, SBS44

Table S6: Active signatures in each of the cancer types, according to the PCAWG analyses. With asterisks, signatures which are considered exogenous for these analysis: those include signatures known to represent exogenous mutational processes (based on the COSMIC annotation of signatures), as well as SBS1 and SBS5. The last signature is used as baseline. Should this signature be exogenous, the last signature without an asterisk is used as baseline.

Cancer type	Group	Min	Max	Mean	Median
Bone-Osteosarc	Clonal	152	11274	3393.78	2986
Bone-Osteosarc	Subclonal	227	3635	1370.59	1334
Breast-AdenoCA	Clonal	557	24655	4351.21	3170
Breast-AdenoCA	Subclonal	136	40540	2663.91	1643.50
CNS-GBM	Clonal	1477	9482	5119.32	5083.50
CNS-GBM	Subclonal	474	175464	6996	1479
CNS-Medullo	Clonal	121	3413	893	646.50
CNS-Medullo	Subclonal	57	2151	508.78	368
CNS-PiloAstro	Clonal	16	645	125.74	79.50
CNS-PiloAstro	Subclonal	31	776	160.95	87
ColoRect-AdenoCA	Clonal	4105	719890	73922.03	10462
ColoRect-AdenoCA	Subclonal	595	1938252	80149.54	2993
Eso-AdenoCA	Clonal	1839	62315	16195.14	15277
Eso-AdenoCA	Subclonal	551	36173	8683.60	6798
Head-SCC	Clonal	1496	42607	11117.69	6871.50
Head-SCC	Subclonal	211	18413	3773.16	3078
Kidney-ChRCC	Clonal	190	6044	988.26	709.50
Kidney-ChRCC	Subclonal	128	4078	896.79	836
Kidney-RCC.clearcell	Clonal	823	23326	5027.29	4014.50
Kidney-RCC.clearcell	Subclonal	249	12993	1704.60	1371
Kidney-RCC.papillary	Clonal	1268	9675	4435.30	4292
Kidney-RCC.papillary	Subclonal	251	5075	1046.67	756
Liver-HCC	Clonal	1420	50963	9800.13	9089
Liver-HCC	Subclonal	268	20069	2880.89	1968
Lung-SCC	Clonal	4811	52291	29414.12	27600
Lung-SCC	Subclonal	515	20221	7266.18	6332.50
Lymph-BNHL	Clonal	1281	47499	7076.04	4521
Lymph-BNHL	Subclonal	181	19374	1658.04	991
Lymph-CLL	Clonal	249	3231	1596.25	1529
Lymph-CLL	Subclonal	176	4083	883.57	649
Ovary-AdenoCA	Clonal	1003	29855	6480.45	5455
Ovary-AdenoCA	Subclonal	193	10605	2436.48	1802
Panc-AdenoCA	Clonal	1145	26491	4374.25	3472
Panc-AdenoCA	Subclonal	180	29039	2377.83	1641
Panc-Endocrine	Clonal	98	8483	1692.56	1178.50
Panc-Endocrine	Subclonal	104	17275	1636.61	891
Prost-AdenoCA	Clonal	118	13366	2495.90	1761
Prost-AdenoCA	Subclonal	382	7083	1527.89	1164
Skin-Melanoma.cutaneous	Clonal	2841	767734	75208.07	30374
Skin-Melanoma.cutaneous	Subclonal	367	82458	12788.90	4105
Stomach-AdenoCA	Clonal	1345	75901	14993.20	8200
Stomach-AdenoCA	Subclonal	429	246341	13698.73	4192
Thy-AdenoCA	Clonal	95	2322	828.51	614
Thy-AdenoCA	Subclonal	88	3177	641.27	533
Uterus-AdenoCA	Clonal	1689	207015	12120.40	3985.50
Uterus-AdenoCA	Subclonal	338	29388	5219.82	2652.50

Table S7: Summary statistics of the number of mutations in each cancer type and group, across patients.

Supplementary methods

S1 Signature extraction

Consider that we are given N samples or sets of mutations and have defined F features summarizing genomic changes (e.g., $F = 96$ in COSMIC signatures), indexed by $j \in [N]$ and $f \in [F]$, where $[x]$ denote the sequence $1, \ldots, x$. The data set can then be written as a count matrix $\mathbf{V} \in \mathbb{N}^{F \times N}$, where each row represents one class of mutations (e.g. one row could represent counts of `ACA`→`ATA` mutations for each sample). The signature definition matrix $\mathbf{S} \in \mathbb{R}_+^{F \times K}$ corresponds column-wise to a probability distribution over all features, for each signature, indexed by $k' \in [K]$, and as such $\sum_f s_{fk'} = 1 \; \forall \; k'$. Note that in this manuscript we use the index k' for signatures and k for signature log-ratios. $\mathbf{S}$ was initially defined by non-negative matrix factorisation (NMF) with an initial set of samples, and it is redefined and maintained by COSMIC. We assume it is known. Let $y^*_{k'j}$ be the number of mutations attributed to signature k' in observation j, i.e. $\mathbf{Y}^* \in \mathbb{N}^{K \times N}$ is the *exposure matrix*. Exposures are estimated by decomposing the count matrix $\mathbf{V}$ into the (given) signatures $\mathbf{S}$ and their exposures in each sample $\mathbf{Y}^*$. $\mathbf{Y}^*$ can be found from $\mathbf{V}$ and $\mathbf{S}$ via quadratic programming [18]. The exposure matrix $\mathbf{Y}^*$ is our matrix of interest. In the methods section of this paper, for notational convenience, the transposed exposure matrix $\mathbf{Y} = (\mathbf{Y}^*)^\top$ is used instead of $\mathbf{Y}^*$.

The signatures of PCAWG samples have been extracted using quadratic programming after extracting trinucleotide counts in a custom code, as well as using `mutSigExtractor` [19] on the vcf files (Fig S1) for this comparison, as well as a comparison to values reported in [20] and extracted using `sigProfiler`. As patient ids are not available for download in the PCAWG data Synapse repository, these results are at the level of signature and cancer type.

S2 Compositional data

The absolute number of mutations quantified using next generation sequencing methods is inherently compositional. For instance, a higher number of mutations attributable to SBS1 in the clonal group than in the subclonal group might be attributable to a higher mutation rate of SBS1 in the clonal group, but we cannot rule out a variety of different scenarios: that clonal mutations have higher coverage

and are therefore more easily detected by SNV-calling methods, that cells containing predominantly clonal mutations are more abundant than cells that have accumulated more subclonal mutations in the sample, that a longer time has elapsed in the generation of clonal mutations leading to a higher number of mutations independently of any change in the mutation rate.

In the case under consideration, for the jth observation, we can deduce y_{jK}, the number of mutations corresponding to the last signature (also known as the exposure of signature k in observation j), as $T_j - \sum_{k=1}^{K-1} y_{jk}$, where T_j denotes the mutational toll of observation j (and to which exposures have been allocated). This makes such vectors $(K-1)$-dimensional and compositional data, lying on a space called the simplex. The additive log-ratio transformation (ALR) and inverse additive log-ratio transformation (ALR^{-1}) transformations correspond respectively to the logit and expit transformations often used in multinomial regressions. [48] address the common approaches and challenges related to the analysis of compositional data in more depth. Finally, we preferred the ALR transformation to the isometric log-ratio transformation (ILR) and centered log-ratio transformation (CLR) as CLR-transformed values cannot be used in regressions settings (as their covariance matrix is not full-rank), whilst the ALR and ILR yield the same results in this such setting, the ALR being a linear transformation of ILR. Moreover, if additional signatures are used in the model, the parameters in ALR space will not vary (provided the new signature is not used as baseline), whereas all parameters in ILR space would. In Section S2.2 we show the equivalence of results obtained when different signatures are used as ALR basis.

S2.1 Presence of zeros in signature exposures

The presence of zeros is a notorious complication for compositional models. In our model it is the coefficients $\boldsymbol{\beta}_0, \boldsymbol{\beta}_1$ (as well as the multivariate intercepts) which are in ALR space - note that we do not take the ALR transformation of the counts for each observation, where zeros do occur and where this would be problematic, possibly warranting replacement of zero exposures by a small non-zero value, which in turn could cause undesirable results in the model. Instead, the response of model is count data modelled by a multinomial distribution, where zero is included in the support.

It would be problematic, however, to include signatures of extremely low exposure, as it would lead to $\boldsymbol{\beta}$ coefficients being 0 or $\to \infty$ (and this would apply to both $\boldsymbol{\beta}_0$ and $\boldsymbol{\beta}_1$). This is the reason why only active signatures are used. Similarly, if a signature is only present in the first group, $\boldsymbol{\beta}_0$ can be

estimated, but $\boldsymbol{\beta}_1$ cannot, as $\boldsymbol{\beta}_{1j} \to -\infty$.

PCAWG exposures data do often contain zeros, as shown in Table S3: the fraction of zero exposures in a dataset varies from 2.7% to 38.6%, and, for a single signature, the fraction of samples with a zero exposure can be as high as 93%.

Simulation C1 contains sparse exposures, as they are created by mixing in varying proportions exposures from two cancer types in which only a small fraction of signatures overlap. Therefore, it represents a scenario where some signatures the subclonal exposures of which are zero, and the clonal exposures of which are not zero, or vice versa, where π is high - i.e. where the first group represents mutations from a cancer type and the second group mutations from the other. It is of note that after signature re-extraction these exposures are small but never zero in all patients. For SBS3 and SBS13, the exposures are practically 0 in the first group (they account for 2% and 0.3% of mutations across patients). For SBS1 and SBS9, they are almost 0 in the second (they account for 2% of mutations across patients). The model still recovers the coefficients satisfactorily.

S2.2 Choice of baseline for ALR

The choice of baseline signature is not important for differential abundance results - both the softmax $\widehat{\boldsymbol{\beta}}$ and the p-values are the same. We show these results by extending the simulation paradigm B2, with the following change: we modify the $\boldsymbol{\beta}_1$ coefficients so that all $\boldsymbol{\beta}_1$ are equal to a small number (0.1), which corresponds to only the last signature (SBS13) being differentially abundant. This allows us to have a scenario of subtle differential abundance, with a spread of p-values, but which also allows us to use as baseline either the signature that corresponds to the differentially-abundant signature, or another signature (here chosen as the fourth signature, SBS8, which is the signature of highest abundance in the whole dataset). The results are shown in Fig S30.

S3 Implementation of the models

Our results might benefit from the good properties of LA in regards to bias and mean-square errors [49] as well as convergence rate and coverage [50]. Speed is also an attractive feature of the Laplace approximation compared to alternatives [28], as well as the fact that no parameters for integration, such

as the number of quadrature points, have to be specified. In the model in [27], the computational Gauss-Hermite quadrature is used to approximate the random effect integrals instead.

S4 Assessment of the proposed models

S4.1 Bias and coverage of the estimator

The first set of simulations, referred to as **A1-A3**, are generated assuming

- a case involving $K = 5$ signatures and a number of mutations per sample of $T_j = 180$,
- a positive-definite matrix with no zero off-diagonal values in the random effect covariance matrix

$$\Sigma = \begin{pmatrix} 1.5 & -1.3 & -1.26 & 02 \\ -1.3 & 2.55 & 1.4 & -0.01 \\ -1.26 & 1.4 & 1.35 & -0.02 \\ 02 & -0.01 & -0.02 & 0.09 \end{pmatrix},$$

- varying precision parameter values, $\lambda = \{2, 20, 100\}$, assumed equal per group (i.e., $\lambda_j = \lambda \; \forall \; j$),
- $\boldsymbol{\beta}_0 = [5.5, 0.9, 0.35, 3]$ and $\boldsymbol{\beta}_1 = [-0.27, -1.28, 0.97, -1.28]$.

These sets of simulations, generated with lambda $\lambda = \{2, 20, 100\}$ respectively. We focus here on the results corresponding to the models `diagREDM` and `fullREDM`. Both models allow the precision parameter to be group-dependent as well as allowing for within-patient dependence, but differ in regards to the random effects correlation. In `diagREDM` and `fullREDM`, the random effects of the different signatures are respectively assumed to be independent and possibly dependent. Figs S2 and S3 respectively show the results for the `diagREDM` and `fullREDM` models. The upper plots show the bias (y-axes) for each of the $K - 1 = 4$ log-ratios of signatures (x-axes) for each element of $\boldsymbol{\beta}_0$ (left) and $\boldsymbol{\beta}_1$ (right) for three fixed values of λ (plots). The lower plots shows the coverage of 95% confidence intervals (y-axes) for the same configurations. The yellow bands correspond to the Monte Carlo tolerance area (defined by the quantiles 0.025 and 0.975 of a binomial with 1000 draws and a probability of success of 95%, the theoretical coverage). We can note that `diagREDM` shows biases for the $\boldsymbol{\beta}_0$ elements, leading to very poor

observed coverages. Estimates of the elements of $\boldsymbol{\beta}_1$ are however reliably estimated with good coverages, suggesting that assuming an independence structure for the random effects is not problematic for the target parameter inference. Comparatively, `fullREDM` typically obtains lower bias and better coverages for elements of $\boldsymbol{\beta}_0$. For $\boldsymbol{\beta}_1$, `fullREDM` and `diagREDM` lead to similar results for large values of λ and worse results for $\lambda = 2$, corresponding to a case with extremely low level of precision. However, in Fig S4, which shows the equivalent results for `singleREDM` we showcase the need for multivariate intercepts, as these biases and poor coverages are exhacerbated when using intercepts drawn from a single σ. In fact, as it follows from the softmax transformation, this model leads to the random effect only changing the abundance of the last signature, which is used as baseline. To summarise, our results suggest that, if assuming independence between the random effects lead to biases in the estimation of elements of $\boldsymbol{\beta}_0$, this does not seem to translate to elements of $\boldsymbol{\beta}_1$, our parameter of interest, provided multivariate random effects are used. Due to the strong reduction in the number of parameters to estimate, `diagREDM` thus appear as very attractive compared to `fullREDM`, whilst not being as restrictive as using a diagonal covariance matrix with shared elements along the diagonal.

Additionally, we performed equivalent simulations, considering `diagREDM` and `fullREDM`, for biologically-relevant parameters, taken from the PCAWG cohort by estimating the parameters using the `diagREDM` (in the case of uncorrelated data) and `fullREDM` models (in the case of correlated data). Three simulations are shown: (B1) for CNS-GBM without any signature correlations, (B2) for Lung-SCC without any signature correlations, and (B3) for Lung-SCC with positive and negative signature correlations. In all cases 1000 Monte Carlo simulations are performed for 200 samples.

The parameters are as follows. For simulation (**B1**) using parameters from CNS-GBM without any signature correlations, the parameters are

- $K = 6$ signatures and a number of mutations per sample of $T_j = 3401$ (median of the observed number of mutations),
- a positive-definite matrix of zero off-diagonal values in the random effect covariance matrix

$$\Sigma = \begin{pmatrix} 7.8e-06 & 0 & 0 & 0 & 0 \\ 0 & 0.45 & 0 & 0 & 0 \\ 0 & 0 & 1.19 & 0 & 0 \\ 0 & 0 & 0 & 5.8 & 0 \\ 0 & 0 & 0 & 0 & 0.37 \end{pmatrix},$$

- precision parameter value $\lambda = 18$, assumed equal per group (i.e., $\lambda_j = \lambda \ \forall \ j$, corresponding to the average between the two estimated λ),
- $\boldsymbol{\beta}_0 = [-0.38, -1.13, -3.6, -6.96, -2.48]$ and $\boldsymbol{\beta}_1 = [-0.43, -0.17, 0.41, 1.03, -0.06]$.

For simulation (**B2**), using parameters from Lung-SCC without any signature correlations, the parameters are

- $K = 6$ signatures and a number of mutations per sample of $T_j = 14072$ (median of the observed number of mutations),
- a positive-definite matrix with no zero off-diagonal values in the random effect covariance matrix

$$\Sigma = \begin{pmatrix} 0.27 & 0 & 0 & 0 & 0 \\ 0 & 0.02 & 0 & 0 & 0 \\ 0 & 0 & 1.73 & 0 & 0 \\ 0 & 0 & 0 & 0.69 & 0 \\ 0 & 0 & 0 & 0 & 0.59 \end{pmatrix},$$

- precision parameter value $\lambda = 80$, assumed equal per group (i.e., $\lambda_j = \lambda \ \forall \ j$, corresponding to the average between the two estimated λ),
- $\boldsymbol{\beta}_0 = [-2.56, -0.39, 1.53, 1.77, 0.37]$ and $\boldsymbol{\beta}_1 = [1.07, 0.04, -0.74, -0.37, 07]$.

For simulation (**B3**) using parameters from Lung-SCC including a covariance matrix with positive and negative signature correlations, the same parameters as in (2) are used, except for $\boldsymbol{\lambda}$ and $\boldsymbol{\Sigma}$:

- positive-definite matrix with no zero off-diagonal values in the random effect covariance matrix

$$\Sigma = \begin{pmatrix} 0.63 & 0.53 & 0.03 & 0.47 & 0.25 \\ 0.53 & 0.2 & -0.38 & -0.36 & -0.58 \\ 0.031 & -0.39 & 1.33 & 0.8 & 0.82 \\ 0.47 & -0.37 & 0.8 & 0.9 & 0.96 \\ 0.25 & -0.58 & 0.82 & 0.96 & 0.81 \end{pmatrix},$$

- a precision parameter value $\lambda = 87$, assumed equal per group (i.e., $\lambda_j = \lambda \; \forall \; j$), corresponding to the average between the two estimated λ).

Fig S5 shows the inference results using the un-correlated mixed-effects DM (`diagREDM`) and correlated mixed-effects DM (`fullREDM`) from simulation scenario (B1) of data simulated using previously-estimated parameters from the CNS-GBM cohort, with $N_s = 200$, no correlations, and a shared $\boldsymbol{\lambda}$. All remaining parameters are taken as estimated. Given that uncorrelated data are simulated, both models give the same results for bias and coverage. Both $\boldsymbol{\beta}_0$ and $\boldsymbol{\beta}_1$ are well recovered, although the penultimate element of $\boldsymbol{\beta}_0$ has some biases. This corresponds to the $\boldsymbol{\beta}_0$ in which the numerator of the log-ratio is the signature of lowest abundance.

In Fig S6 the equivalent simulation as above is carried out, but this time data are simulated from scenario (B2) using previously-estimated parameters from the Lung-SCC cohort, with $N_s = 200$, no correlations, and a shared $\boldsymbol{\lambda}$ (average between the two estimated λ). All remaining parameters are taken as estimated. Again, given that uncorrelated data are simulated, both models give the same results for bias and coverage. Bias and coverage results are satisfactory for both $\boldsymbol{\beta}_0$ and $\boldsymbol{\beta}_1$.

In Fig S7 data are simulated from the Lung-SCC cohort in simulation scenario (B3), with $N_s = 200$ and a shared $\boldsymbol{\lambda}$ (average between the two estimated λ). However, unlike (B1) and (B2), all remaining parameters are taken as estimated from the `fullREDM` model, and this includes correlations. The results for bias and coverage differ between the models owing to the presence of correlations. Bias and coverage results are satisfactory in both cases for $\boldsymbol{\beta}_1$, but there is bias and low coverage for $\boldsymbol{\beta}_0$ in the non-correlated `diagREDM` model (Figs S7a and S7c), as expected.

In conclusion, the results of these simulations – including simulations in which values are chosen to be representative of this type of data – indicate that $\boldsymbol{\beta}_0$ might be more difficult to recover if the

simpler model `diagREDM` is used, whereas $\boldsymbol{\beta}_1$ is still well recovered. These results are consistent across simulations.

S4.2 Effect of the number of mutations on bias and coverage

The effect of the number of mutations on bias and coverage is assessed with biologically-informed simulation **B4**. Using parameters from Eso-AdenoCA without any signature correlations, the parameters are

- $K = 10$ signatures
- a varying number of mutations per sample: (1) equal to the observed number of mutations, and preserving the group (clonal/subclonal) information, ranging from 551 to 62315 ('observed T'), (2) values half the size, ranging from 276 to 31158 ('lower T'), (3) values ten times smaller, ranging from 55 to 6232 ('lowest T').
- a positive-definite matrix with no zero off-diagonal values in the random effect covariance matrix

$$\Sigma = \begin{pmatrix} 1 & 0.18 & -0.27 & 0.93 & 0.17 & -0.44 & -0.37 & 0.73 & -0.49 \\ 0.18 & 1 & 0.18 & 0.16 & 0.96 & 0.19 & 0.27 & -0 & -0.09 \\ -0.27 & 0.18 & 1 & -0.09 & 0.26 & 0.02 & 0.04 & -0.45 & 0.17 \\ 0.93 & 0.16 & -0.09 & 1 & 0.14 & -0.23 & -0.17 & 0.55 & -0.22 \\ 0.17 & 0.96 & 0.26 & 0.14 & 1 & 0.12 & 0.19 & 0.13 & -0.08 \\ -0.44 & 0.19 & 0.02 & -0.23 & 0.12 & 1 & 0.99 & -0.37 & 0.72 \\ -0.37 & 0.27 & 0.04 & -0.17 & 0.19 & 0.99 & 1 & -0.36 & 0.65 \\ 0.73 & -0 & -0.45 & 0.55 & 0.13 & -0.37 & -0.36 & 1 & -0.46 \\ -0.49 & -0.09 & 0.17 & -0.22 & -0.08 & 0.72 & 0.65 & -0.46 & 1 \end{pmatrix},$$

- two precision parameters with values $\lambda^{(1)} = 105.4$ and $\lambda^{(2)} = 50.4$,
- $\boldsymbol{\beta}_0 = [-0.87, -2.2, 8 - 3.67, -0.10, -3.17, -0.76, -0.03, -0.84, -3.21]$
- $\boldsymbol{\beta}_1 = [-0.27, 0.17, -0.19, -0.28, 0.05, -0.63, -0.7, 0 - 0.33, -0.21]$.

S4.3 Bias and coverage simulating from PCAWG data

Fig S10 shows the bias and coverage for datasets simulated based on each of the cohorts in PCAWG. `diagREDM`, as reported in the paper, has been used to estimate all parameters, and the same parameters are used to generate data under a Dirichlet-multinomial with random intercepts. The estimated values used for simulation are $\boldsymbol{\beta}$, $\boldsymbol{\lambda}$, and uncorrelated covariance matrices. The number of mutations is shared for all observations in each clonal/subclonal group, and equal to the median number of mutations in the corresponding group of the observed datasets. Bias and coverage of $\widehat{\boldsymbol{\beta}_0}$ and $\widehat{\boldsymbol{\beta}}_1$ are assessed for the model `diagREDM`, indicating very satisfactory results for all cases, especially for $\widehat{\boldsymbol{\beta}}_1$, with biased results only for an individual coefficient in Head-SCC, and excellent coverage and unbiased estimates elsewhere.

S4.4 Comparison with previous models

In this section we compare the output of our model to that of HiLDA and TCSM. We wanted to mention, additionally, [27], who they describe, from a theoretical perspective, a multivariate and unconstrained structure for the random effects as well as a covariate-dependent overdispersion parameter. Their estimator and implementation actually only consider a single random intercept parameter for all log-ratios of categories as well as a shared overdispersion parameter, thus imposing strong assumptions on the data dependence structure and variability, and the models we present here are extensions of this model.

S4.5 CCF regression

S4.5.1 Data simulation: C1, C2, C3

The need to generate trinucleotide mutations The simulation of data which can be used as input for TCSM and HiLDA is more complex than the simulation of data for *CompSign*, as both TCSM and HiLDA take as input substitution categories. In the case of TCSM the input is a trinucleotide substitutions matrix (with a total of $F = 96$ possible trinucleotide substitutions). In the case of HiLDA it is a vcf file listing all trinucleotide mutations. Moreover, in HiLDA signature extraction is performed using the pmsignature model by [51], which requires specifying the custom set of genomic features to

use. Here we use, again, the single-base substitution together with the two flanking bases, without any strand specificity. The features are treated as independent in the pmsignature model, unlike the combinatorial approach of COSMIC and TCSM signatures that leads to $F = 96$.

Generation of trinucleotides We generate trinucleotide mutations in two groups, which we call group 1 and group 2, and which are equivalent to clonal and subclonal groups. Trinucleotides in group 1 for patient i are drawn from a single distribution $W_i^{(1)}$. Trinucleotides in group 2 for patient i are drawn from a mixture of distributions $W_i^{(1)}$ and $W_i^{(2)}$, with a mixing proportion π. This is a simulation framework very similar to the one used in [30]. In particular, let $1-\pi, \pi \in [0,1]$ be the proportions in which $W_i^{(1)}$ and $W_i^{(2)}$ are mixed to create trinucleotide mutations in group 2. For low values of π, trinucleotides from group 2 are mostly drawn from the same distribution as trinucleotides from group 1 (no differential abundance), whereas for high values of π the trinucleotides from group 1 are drawn from one distribution and for group 2 from another (differential abundance). With increasing values of π, differential abundance becomes more evident. The mixing proportions π take the following values, in percentage: $\{0.034, 0.091, 0.250, 0.670, 2.3, 2.9, 3.7, 4.7, 6, 7.6, 9.5, 12, 27, 50, 62, 73\}$, and we have simulated 10 datasets with each set of parameters.

Simulation of trinucleotides from ground-truth exposures Trinucleotide sampling given a vector $W_i^{(1)}$ (and equivalently for $W_i^{(2)}$) is done as follows. First, we establish that W_i is a vector of relative exposures (that sums to 1). In the case of trinucleotides from group 1, each of the mutations $l = 1, \ldots L_i^{(1)}$ that we simulate for patient i in the group under consideration are drawn by first sampling a signature from the ground truth exposure W_i^1:

$$\text{sig}_{l,i}^{(1)} \sim \text{Cat}(W_i^{(1)})$$

and then drawing a trinucleotide from the signature definition matrix of signature $\text{sig}_{l,i}^{(1)}$:

$$\text{trinucleotide}_{l,i}^{(1)} \sim \text{Cat}(S_{\text{sig}_{l,i}^{(1)}})$$

In the case of trinucleotides from group 2, where both $W_i^{(1)}$ and $W_i^{(2)}$ are used to create the dataset,

we first partition the total number of mutations to generate for patient i in group 2 in $l = 1, \dots L_i^{(2)}$ according to π, and then trinucleotides are drawn as above, drawing a most mutations from $W_i^{(1)}$ if π is low, and a most from $W_i^{(2)}$ if π is high.

Origin of $W_i^{(1)}$ and $W_i^{(2)}$ The exposures $W_i^{(1)}$ and $W_i^{(2)}$ from which we simulate mutations differ in simulated datasets C1-3:

C1: $W_i^{(1)}$ are exposures from Lymph-CLL (using 4 signatures) and $W_i^{(2)}$ are exposures from Breast-AdenoCA (using the 4 most abundant signatures). This gives a matrix W with a total of six signatures, including signatures that are present both in Lymph-CLL and Breast-AdenoCA (SBS5, SBS40) but also signatures particular to Lymph-CLL (SBS1, SBS9) and to Breast-AdenoCA (SBS3, SBS13).

C2: $W_i^{(1)}$ are exposures from the clonal and subclonal groups of Lymph-CLL. No information about patient pairing is used, i.e. for each simulated patient, trinucleotides represent a mixture of a random row i $W_i^{(1)}$ and a random row i $W_i^{(2)}$.

C3: $W_i^{(1)}$ are exposures from the clonal and subclonal groups of Lymph-CLL. The information about patient pairing is preserved, i.e. for each simulated patient, trinucleotides represent a mixture of a given row in $W_i^{(1)}$ and the respective row in $W_i^{(2)}$.

Input for the models In the case of TCSM, which is run on the command line, this trinucleotide substitution matrix and the covariate matrix are the only two input files. In the case of HiLDA, we synthetically create a *pmsignature* object from the trinucleotide substitution matrix. The number of signatures which both HiLDA and TCSM need as input is given as the number of signatures used in the simulation. In the case of *CompSign*, signatures need to be extracted first - we do so using quadratic programming, exactly in the same way as it is done for the PCAWG samples in the Results section, and specifying the signatures from which we have simulated data. The extracted signature matrix is used as input for the *CompSign* `diagREDM` model.

S4.5.2 Comparison of $\boldsymbol{\beta}_1$ to equivalent parameters in HiLDA and TCSM

Transformation of coefficients To assess the agreement in the estimated parameters of differential abundance, we have had to transform the output of each of the models. For `diagREDM` this has been done by taking the softmax-transformed $\widehat{\boldsymbol{\beta}}_1$, giving a vector of lentgh d. For HiLDA the values $\widehat{\boldsymbol{\alpha}}$ are the two estimated parameters for Dirichlet-multinomial distributions, arranged in two columns for the clonal and subclonal group respectively. Therefore, the $\widehat{\boldsymbol{\beta}}_0$ for HiLDA is the first column of $\widehat{\boldsymbol{\alpha}}$, $\widehat{\boldsymbol{\alpha}}_0$, normalised to sum to one ($\widehat{\bar{\boldsymbol{\alpha}}}_0$), whereas $\boldsymbol{\beta}_1$ is $\text{ALR}(\widehat{\bar{\boldsymbol{\alpha}}}_1) - \text{ALR}(\widehat{\bar{\boldsymbol{\alpha}}}_0)$, which has also been softmaxed for comparison. For TCSM, the softmax transformation of the second column of the “effects“ matrix is used: this is also a signature-specific vector that indicates differential abundance the more it differs from zero.

Order of coefficients Whenever these coefficients are compared to the ground truth $\boldsymbol{\beta}_1$, they need to be sorted. In the case of `diagREDM` they are already in the same order as the ground truth. In the case of HiLDA, the comparison is done by first sorting signatures according to abundance in the clonal group, whereas TCSM coefficients have been sorted by first matching their estimated exposures with ground-truth exposures based on their cosine similarity.

Computing empirical $\boldsymbol{\beta}_1$ from the simulation The data in simulations C1-3 have not been simulated under the model in order not to favour any of the three methods - rather, the data have been generated by mixing in varying proportions mutations that are drawn from two matrices of mutational signatures. Despite not having ground truth $\boldsymbol{\beta}$ coefficients *per se*, we have estimated them based on the ground truth signature exposures from which trinucleotide mutations are drawn (and from which, in turn, exposures are re-extracted). To compute the empirical ground truth values for $\boldsymbol{\beta}_1$, the empirical ground truth values for $\boldsymbol{\beta}_0$ need to be computed first. This is done by normalising to one each of the clonal exposures in the simulation, adding them together signature-wise, and re-normalising to one. This gives the equivalent of $\bar{\boldsymbol{\alpha}}$ for the clonal group. The same is done for the subclonal group. $\boldsymbol{\beta}_0$ corresponds to $\bar{\boldsymbol{\alpha}}^{\text{ALR}}_{\text{clonal}}$, and $\boldsymbol{\beta}_1$ corresponds to $\bar{\boldsymbol{\alpha}}^{\text{ALR}}_{\text{subclonal}} - \bar{\boldsymbol{\alpha}}^{\text{ALR}}_{\text{clonal}}$.

S4.5.3 Comparison of $\boldsymbol{\beta}_0$ to equivalent parameters in HiLDA and TCSM

Transformation of coefficients To assess if the estimated abundances of signatures are in agreement with the ground truth, we have plotted the abundance of signatures in probability space. For `diagREDM` this has been done by taking the softmax-transformed $\widehat{\boldsymbol{\beta}}_0$, for HiLDA by normalising $\widehat{\boldsymbol{\alpha}}_0$ so that $\sum_1^K \widehat{\boldsymbol{\alpha}}_{0k} = 1$ (as shown above), and for TCSM by using the first column of the "effect" matrix, which is already a probability vector.

Order of coefficients This has been done in the same way as for $\boldsymbol{\beta}_1$ (Section S4.5.2) unless specified otherwise. In Fig S16 they have been ordered by signature abundance, to ensure the best possible match between HiLDA, TCSM and `diagREDM`.

Computing empirical $\boldsymbol{\beta}_0$ from the simulation See Section S4.5.2.

S4.5.4 Assessment of signature recovery from TCSM

The cosine similarities from Fig S17 are computed as follows: for each estimated signature in the definition matrix $\hat{\mathbf{S}}^{\text{TCSM}}$ we compute its cosine similarity to all COSMIC signatures $\mathbf{S}^{\text{COSMIC}}$. We then pair each estimated signature to the COSMIC signature that represents it best, and ensuring that no two estimated signatures are paired to the same COSMIC signature, by sequentially pairing the estimated signature of highest cosine similarity. This gives, for each run of TCSM (equivalently, each simulated dataset) a set of cosine similarities of length d. High cosine similarities are indicative of a good recovery of signature definitions.

S4.5.5 Assessment of signature exposure recovery

The ground truth exposures from the simulation to the exposures re-extracted using quadratic programming which are used input for `diagREDM`, and the newly estimated exposures from HiLDA and TCSM, are compared to the ground truth exposures of simulations C1-3. The Pearson correlations of these exposures are found in Fig S18, showing that the estimated exposures from HiLDA and the re-extracted exposures from quadratic programming are much more representative of the ground truth exposures than the TCSM exposures are, in all three simulations, and in simulation C1, when a large number of

mutations are included, TCSM recovers the exposures reasonably well. For quadratic programming, the number of mutations used is also a determinant factor for correct signature extraction; provided the number is representative of the number of mutations in the dataset (in this case, 1773), the exposures are perfectly recovered in C1 and C3.

S4.5.6 Comparison of runtime

All models have been run on a machine with 8 CPU and 8GB RAM. HiLDA, insofar as it is a Bayesian model, is slower than TCSM and `diagREDM`, and we were unable to run it in several datasets with a high number of mutations, as its runtime exceeded the 10-hour and even 24-hour mark. These appear as missing in Table S5 and any figures relating to simulated datasets C1 or C3. For HiLDA, the function `hildaTest` is used and timed within R. For TCSM a two-step process is needed: the `run_tcsm.R` is run, followed by `estimate_significance.py`; both are timed.

S4.5.7 Effect of active signature selection on differential abundance

We have extended the simulation strategy based on the simulation of mixtures so that we can compare the results of our models when we extract signatures using four types of subsets of active signatures. These datasets, (**C2B**), have been created in the same way as (C2), but using exposures from the four most abundant signatures in Prost-Adenoca. We consider four strategies for active-signature selection: the "true" subset of signatures used in the simulation (*Simulated signatures*), and three sets of active signatures determined by first extracting signatures de novo using all COSMIC signatures, and then refining them by selecting the top signatures that contribute to a total of 80% of mutations in a sample (*Signature sum > 80% samples*), or to a 60% of the samples (*Signature sum > 60% samples*), or signatures which are present (non-zero) in at last 80% of samples (*Signature active > 80% samples*). The signatures which are considered to be active in strategy 3 are a subset of those active in strategy 2.

S4.6 Assessment of the robustness of results in the PCAWG data

S4.6.1 Assessment of the robustness of results as active signatures change

The PCAWG datasets have been modified in a variety of ways to ensure that the results are robust to small changes in the dataset. Those simulations are D1A, D1B, D2C and D1D. Unless specified otherwise, quadratic programming is used for signature extraction. Overwhelmingly, these results of differential abundance are robust to changes in the set of active signatures in the cancer type.

D1A and D1B For D1A, signatures are re-extracted after modifying the set of active signatures (where one active signature is removed at a time). For D1B, signatures are re-extracted after modifying the set of active signatures (where one active signature is added at a time; in total four non-active signatures in each cancer type are selected at random). Signatures are extracted with this new set of signatures (we have used both quadratic programming, as used in the main results of the paper, and mutSigExtractor, for comparison). Following signature extraction, the model `diagREDM` is run, and the results of inference are compared.

We note that there is no change in the differential abundance results, at a significance of 0.05, in the vast majority of cases where a signature has been added or removed from the analysis. In Fig S20a, we count the number of simulations within each cancer type in which removing one signature at a time (left) or adding one additional signature (right) yields results in agreement with the unmodified dataset (in pale green), in disagreement (in red), or non-convergent results (in pale red). In the leave-one-out analysis there are very few cases of disagreement - corresponding to cancer types in which the removed signature contributed heavily to differential abundance. In the add-one case, there are some cancer types in which adding a certain signature gives non-convergent results: these are signatures of true negligible abundance, and the model does not converge because their $\boldsymbol{\beta}_0$ tends to $-\infty$.

These results are replicated when signature extraction is performed using mutSigExtractor (Fig S20b).

The overwhelming agreement in differential abundance gives us confidence that the results are not overly sensitive to the set of active signatures used in the analysis.

Secondly, we compare the estimates of $\boldsymbol{\beta}$ in D1A and D1B to the estimates of $\boldsymbol{\beta}$ from the original set of active signatures. In D1A we compare the $\boldsymbol{\beta}$ of corresponding to all signatures except the signature that has been removed. When it is the last signature that has been removed, as this signature

corresponds to the baseline, the comparison of $\boldsymbol{\beta}$ is not possible - hence, we have evaluated the impact of removing each active signature except for the last active signature (the agreement in differential abundance has, on the other hand, been evaluated for all leave-one-out combinations, as shown above). In D1B we compare all $\boldsymbol{\beta}$ that correspond to the original set of active signatures (i.e. $\boldsymbol{\beta}$ corresponding to all signatures in D1B except for the newly-added active signature).

We first show results where quadratic programming has been used for signature re-extraction. In Fig S20c are plotted the correlations between the estimates of $\boldsymbol{\beta}$ in D1A and D1B to the estimates of $\boldsymbol{\beta}$ from the original set of active signatures. Within each cancer type, correlations have been sorted in decreasing order. We note that for both D1A and D1B the correlations are very high in the vast majority of cases.

Removing one signature from the dataset does not have much of an effect on the $\boldsymbol{\beta}$ of the remaining signatures, neither when we consider both $\boldsymbol{\beta}_0$ and $\boldsymbol{\beta}_1$ (first plot in Fig S20c) nor when we only consider $\boldsymbol{\beta}_1$ (second plot in Figure S20c). It is true that for some cancer types - but not all - removing one signature can lead to big changes in the model results (with $\boldsymbol{\beta}$ correlations decreasing even to negative correlations, in very few cases). The most extreme case is that of Lymph-CLL: in this cancer type, upon removal of SBS9, $\boldsymbol{\beta}_1$ change entirely, because in this dataset SBS9 is the sole driver of differential abundance. However, in most cases removing one signature has practically no effect on the $\boldsymbol{\beta}$ of the remaining signatures, with correlations being often ¿ 0.95.

Adding one additional signature has even less of of an effect on the $\boldsymbol{\beta}$ of the remaining signatures, regardless whether we are considering both $\boldsymbol{\beta}_0$ and $\boldsymbol{\beta}_1$ (third plot in Figure S20c) or only $\boldsymbol{\beta}_1$ (fourth plot in Figure S20c). Practically all correlations are well above 0.95.

These results are replicated when mutSigExtractor instead of quadratic programming (Fig S20d).

D1C We have tested the effect of adding "flat" signatures (in the case of SBS5 and SBS40) or "clock" signatures (in the case of all three) by including them or removing them in the PCAWG datasets. For each cancer type, all combinations of SBS1, SBS5, and SBS40 in the active signatures have been considered (ranging from all three absent to all three present, and considering all combinations), while the rest of active signatures in the cancer type have been left unchanged. For each of these new sets of active signatures we have extracted mutational signatures using quadratic programming, and run the

model `diagREDM`. In Fig S21 it is shown whether the result of differential abundance, at a significance level of 0.05, agrees (green) or disagrees (red) with the original dataset. Pink values correspond to datasets for which `diagREDM` did not converge. The cells with a rectangle correspond to the actual set of active signatures: in all but two cases, according to the PCAWG study, all three signatures are active. In most cases, modifying the subset of SBS1, SBS5, SBS40 in the active signatures does not have repercussions in differential abundance. It is important, however, in datasets with few mutational signatures and few samples, such as Bone-Osteosarc or the two CNS cancer types.

D1D Simulation to quantify the effect of including signatures with linear dependencies. Selecting one active signature at a time, test the effect of splitting this signature into two other signatures (the linear combination of which is the original signature) in signature extraction and differential abundance.

The analysis is run for each cancer type independently and the results from the simulation are compared to the results shown in the paper for each cancer type. The simulated data are generated as follows. For each cancer type a signature is selected from the set of active signature at random - we call this signature X. We first create a mutational profile for two alternative signatures, $X*$ and $X\#$, so that a linear combination of the signature definitions of $X*$ and $X\#$ are signature definitions of X. We consider three cases: (a) that in this linear combination the coefficient of $X*$ is 0.75 and the coefficient of $X\#$ is 0.25, (b) that both coefficients are 0.5, (c) that the coefficient of $X*$ is 0.25 and the coefficient of $X\#$ is 0.75. Our expectation is that, if signature extraction is satisfactory, the abundance of X* will be greater in scenario (a), the abundances of $X*$ and $X\#$ will be the same in scenario (b), and the abundance of $X*$ will be lower in scenario (c). In our desired scenario, the abundances of the remaining signatures will not change noticeably.

We show the estimated $\widehat{\boldsymbol{\beta}_0}$ and $\widehat{\boldsymbol{\beta}}_1$ for Kidney-RCC.clearcell as an example (Fig S22), where $X*$ is SBS40$*$ and X$\#$ is SBS40$\#$. The first row corresponds to the estimated values shown in the manuscript, without any modification to the set of signatures, and the other three rows correspond to scenarios (a), (b) and (c) respectively. We note that the relative abundance of $X*$ and $X\#$ is as expected, with $\widehat{\boldsymbol{\beta}_0}$ (SBS40$*$ with respect to SBS41) being higher than $\widehat{\boldsymbol{\beta}_0}$ (SBS40$\#$ with respect to SBS41) in scenario (a), lower in scenario (c), and equal in (b). We see that the estimates for $\widehat{\boldsymbol{\beta}_0}$ are remarkably similar for the rest of signatures (Fig S23a), which is a positive indication of robustness to the set of signatures used

in the analysis. Most importantly, we can see how the estimates for $\widehat{\boldsymbol{\beta}}_1$ are practically indistinguishable from those from the original set of signatures (Fig S23b).

S4.6.2 Assessment of the robustness of results as the number of mutations or samples decrease

D2A Simulation to show that the lower precision in the subclonal group is not merely a reflection of the lower number of mutations in this group.

We expect mutation count to influence precision, but have reason to believe that the higher dispersion in the subclonal group is not merely a reflection of the generally lower number of mutations in the subclonal group based on the observed PCAWG data: four PCAWG cancer types the clonal group contains more mutations and is overdispersed (in Head-SCC, Kidney-RCC.clearcell, Kidney-RCC.papillary and Skin-Melanoma.cutaneous) and in three cancer types the subclonal group contains more mutations and is overdispersed (CNS-GBM, CNS-PiloAst, ColoRect-AdenoCA). This has been assessed through simulation D2A.

In this simulation, two cancer types with more mutations in the clonal than the subclonal group, and a higher dispersion in the subclonal group have been selected (Breast-AdenoCA and Thy-AdenoCA). Separately for each cancer type, the number of mutations in the clonal group is reduced by removing mutations at random. These mutations are removed in sequence over several iterations, and in each iteration signatures are re-extracted and saved. Finally, `diagREDM` is run on the exposures from each iteration. We show the estimated values of $\boldsymbol{\lambda}$ for each group as the number of mutations is reduced. On the x-axis, the number of mutations is indicated as a fold-change of the number of clonal-to-subclonal mutations. As more mutations are removed from the clonal group this value goes from $> 1\times$ (more mutations in the clonal group than in the subclonal group, in earlier iterations) to a value of $1\times$ (as many mutations in the clonal than in the subclonal groups) and to values of $< 1\times$ (where subclonal mutations outnumber clonal mutations, at later iterations).

In the case of Breast-AdenoCA, we see that the two $\boldsymbol{\lambda}$ are unchanged as greater fractions of clonal mutations are removed. Thy-AdenoCA shows a different pattern, with the $\boldsymbol{\lambda}$ for the clonal group indeed decreasing as more clonal mutations are removed (indicating higher dispersion due to poorer signature extraction). In the case of Breast-AdenoCA, the differential $\boldsymbol{\lambda}$ cannot be attributed to the number

of mutations being lower in the subclonal group. In fact, we observe that, even in Thy-AdenoCA, equalising the two groups does not lead to the same $\boldsymbol{\lambda}$. Instead, $\boldsymbol{\lambda}$ become equal only when subclonal mutations are 20% more abundant than clonal mutations (i.e. the amount of clonal mutations is $0.84\times$ the amount of subclonal mutations). This indicates that in Thy-AdenoCA, whilst the higher number of mutations in the clonal group partially explains their lower dispersion, there are additional reasons for the differences in $\boldsymbol{\lambda}$ - for instance subclonal heterogeneity due to the coexistence of several clones.

D2B Simulation to show how inference changes as fewer mutations are included in the PCAWG datasets. A percentage of mutations is removed iteratively. We show that the ability to extract signatures and to estimate $\widehat{\boldsymbol{\beta}_0}$ and $\widehat{\boldsymbol{\beta}}_1$ declines only very slightly as the number of mutations is reduced. In the vast majority of cancer types, up to 60% of mutations can be removed with no noticeable impact on the estimates of $\widehat{\boldsymbol{\beta}_0}$ and $\widehat{\boldsymbol{\beta}}_1$.

Simulation D2B has been devised as follows: for each cancer type separately, 10% of mutations present across patients are removed iteratively. At each step of the iteration, signature exposures are extracted for these progressively emptier trinucleotide mutation matrices. We have used quadratic programming for signature extraction, as used in the PCAWG data shown in the paper. The re-extracted signature exposure matrix is used as input for `diagREDM`, saving the output estimates at each iteration. With 6 iterations, the simulated datasets contain 90%, 80%, ..., 40% of the mutations from the original dataset. 40% of the original number of mutations corresponds to a median number of mutations per patient and group that ranges from of 34 (in CNS-PiloAstro) to 5629 (in Lung-SCC). Five replicates are used for each cancer type to account for the randomness with which mutations are removed at each iteration.

We first inspect the estimates for $\widehat{\boldsymbol{\beta}_0}$, $\widehat{\boldsymbol{\beta}}_1$ and compare them to the values reported in the manuscript (Fig S25a). This correlation of estimates is extremely high in all cases. In the case of $\widehat{\boldsymbol{\beta}}_1$ (Fig S25b), the discrepancy is highest in the case of CNS-PiloAstro (which has very few mutations in both groups, as mentioned by the reviewer), and in a few replicates of Kidney-ChRCC. Overall, there is a very satisfactory recovery of $\widehat{\boldsymbol{\beta}_0}$ and $\widehat{\boldsymbol{\beta}}_1$ as the number of mutations is reduced, and this due foremost because this analysis is performed at the cohort level, and even if some samples have a lower number of mutations, those in the cohort can be sufficient for inference provided the number of samples is high

enough. As a reminder, signatures are re-extracted following mutation subsetting, so any mismatch in $\widehat{\boldsymbol{\beta}_0}$, $\widehat{\boldsymbol{\beta}}_1$ is a reflection of possible problems in signature extraction as much as inference of differential abundance.

After analysing these estimates, we consider whether reducing the number of mutations has an impact on the ability to find differential abundance. We compute the fraction of runs that agree with the original dataset in terms of differential abundance (i.e. where both are differentially abundant, or neither are). Although this fraction goes from 87% (when only 10% of mutations are removed) to 71.3% (when 60% of mutations are), the fraction fluctuates in intermediate values (e.g. there is a 89.6% of agreement when 40]% of mutations are removed), and there is variability among replicates, indicating that in some cancer types which mutations are removed can have important downstream analysis. In other instances, such as, CNS-Medullo, the disagreement reflects progressively lower ability to detect differential abundance by the reduced power, with percentages of agreement starting at 100% when 90% are removing (and detecting differential abundance), and these values decreasing monotonically to 20% when 60% of mutations are excluded.

In conclusion, the cohort-wide results are robust even when the fraction of mutations are reduced drastically, including in cancer types with as few as 500 mutations per group (which is the case for Thy-AdenoCA), which makes the model applicable to datasets where the number of mutations are fewer than in WGS, such as WES.

D2C Simulation to show how inference changes as fewer mutations are included in the PCAWG datasets. The simulation setup is similar setup than in D2B, but in each iteration it is a fraction of matched samples (from the clonal and subclonal group) that are removed. Over 6 iterations, 10% of samples of the original dataset are progressively removed, creating datasets that have 90%, 80%, . . ., 40% of samples compared to the original. 40% corresponds to a number of samples ranging from 22 (in Bone-Osteosarc) to 166 (in Prost-AdenoCA). Five replicates are used to assess the variability of results depending on which samples are randomly chosen to be excluded.

Fig S26 shows that datasets can be greatly reduced in terms of number of samples without this reflecting on $\widehat{\boldsymbol{\beta}_0}$ and $\widehat{\boldsymbol{\beta}}_1$ estimates. The estimates in Head-SCC and Kidney-ChRCC suffer the most, the former because of a poor estimation of $\widehat{\boldsymbol{\beta}_0}$, as it has no issue estimating $\widehat{\boldsymbol{\beta}}_1$ (below).

As a greater number of samples is removed from the analysis, there is a slight tendency to a larger fraction of runs being non-convergent, although this is only critical when 50% of samples are used, where no convergent results can be achieved for the majority of cancer types (Fig S27).

S4.6.3 Assessment of the robustness of results if mutations are misclassified

D3 A percentage (5%, 10%, 20%, 40%) of mutations in trinucleotide space is misclassified - from the subclonal group to the clonal, or vice-versa. Five replicates per cancer type are used to account for the randomness in misclassifying mutations. Signature exposures are re-extracted using quadratic programming, and the model is subsequently run. Fig 4 has been replotted with the new estimates (Figure S28). As a higher number of mutations are misassigned, all $\widehat{\boldsymbol{\beta}}_1$ tend to zero, with lower differential abundance, as expected. To summarise these findings in terms of differential abundance, we have computed the percentages of runs (across cancer types and replicates) where there is an agreement on differential abundance. In Fig S29 we can see how in most cases up to 20% of mutations can be misclassified without this having an effect on the results of differential abundance. When differential abundance changes, it is to go from differential abundance in the original dataset to no differential abundance when the mutations in the dataset have been partially misclassified.

S5 Towards signature-specific differential abundance: minimal perturbation

With the consideration again that the data are still compositional, and that the coefficients in the model that relate to abundance (random effects, $\boldsymbol{\beta}$) represent log-ratios of abundances, we reiterate that a global change in signature exposures cannot be attributed directly to any signature or a set of signatures, and neither can we determine the direction of change in a signature (an increase or a decrease). Having said that, we can put forward which signatures change, and in which direction, with the assumption of minimal perturbation – the assumption that the abundances, in absolute terms, of most signatures do not change. We advise against using this as anything other than a suggestion of what the signatures behind differential abundance might be, and only as a starting point for further validation. Following the minimal perturbation framework, we can use the value $\boldsymbol{\beta}_1^{(mp)}$ which minimises the L1 norm of $\widehat{\boldsymbol{\beta}}_1$

as well as the coeffcient of 0 corresponding to the baseline signature. This is equivalent to computing the median of these d values.

The example in Fig S36 is for the $\boldsymbol{\beta}_1$ for Lymph-BNHL as reported in the main text. The red line is the line of “zero perturbation” and for each $\boldsymbol{\beta}_1$ estimate its 95% confidence interval is shown in blue. We can consider a signature to be differentially abundant if the line of “zero perturbation” is not included in the confidence interval of its $\boldsymbol{\beta}_1$. Note that we draw conclusions for the signatures included in the numerator of the log-ratios, but we are unable to get equivalent results for the baseline signature (although it is used to compute the line of “zero perturbation”). In the case of Lymph-BNHL, in 12/13 signatures there is no change (i.e. $\boldsymbol{\beta}_1$ are along the red line), including SBS1, SBS5 and SBS40, which we do not expect to see change, whereas APOBEC signature SBS13 is “more subclonal” than the rest (with higher $\boldsymbol{\beta}_1$) and, according to the minimal perturbation framework, its absolute abundance increases from clonal to subclonal stages.

In Fig 7 these results are put together with the comparison of $\widehat{\boldsymbol{\beta}}_1$ to the $\widehat{\boldsymbol{\beta}}_1$ of clock signatures. The classification is largely accordant with the comparison to clock signatures: signatures where the abundance decreases mostly have $\widehat{\boldsymbol{\beta}}_1$ lower than $\widehat{\boldsymbol{\beta}}_1^{\text{SBS1}}$ and $\widehat{\boldsymbol{\beta}}_1^{\text{SBS5}}$ (blue cells have a blue border around them), whereas for signatures the abundance of which increases they are higher (green cells have a red border around them).

S6 Additional use cases

S6.1 Chromosome analysis

We have used the model `FEDMsinglelambda`, which is our implementation of Dirichlet-multinomial regression without random effects and a single $\boldsymbol{\beta}$ shared across all patients. This model is applied to the data from the ProstAdenoCA cohort in which we have now extracted exposures for each of the chromosomes (note that four signatures of low abundance have been removed prior to signature exposure to aid with inference). Although there are no chromosome-specific $\boldsymbol{\beta}$ and no random effects are used, the model has a high number of parameters. Of those, all $\boldsymbol{\beta}$ coefficients are plotted in Fig S40. In the design structure the mutations of Chr1 are used as baseline (i.e. the $\boldsymbol{\beta}$ from the first facet are equivalent to $\boldsymbol{\beta}_0$ in the rest of the manuscript, and they indicate the signature abundance in Chr1).

The coefficients corresponding to the rest of chromosomes are the equivalent of several $\boldsymbol{\beta}_1$ and are interpreted as the difference in signature abundance between the chromosome under consideration and Chr1, with values of 0 indicating no differential abundance between the two. Inspecting the $\boldsymbol{\beta}$ values suggests that some chromosomes have similar $\boldsymbol{\beta}$ and points to some signatures with shared patterns across chromosomes or with markedly different patterns. An example of that is HRD signature SBS3: with respect to the abundance of SBS41, the abundance of SBS3 can sometimes be much lower (e.g. in Chr2 or Chr3) or much higher (e.g. Chr17, Chr19) than in Chr1.

Analysing the correlation between chromosomes leads to an interesting pattern where they are split into two groups, roughly split by chromosome size: smaller chromosomes Chr15, 16, 17, 19, 20 and 22 have similar exposures to each other. The rest of chromosomes form another group, with similar exposures among each other and different from those of the first group (Fig S41). This grouping reflects again the distinct pattern of SBS3; while in the first group it is more abundant than in Chr1, in the second group it is less abundant.

S6.1.1 Data simulation: C4

In C4 data to be used in a regression model is generated to be able to test differences between *CompSign* and TrackSig. TrackSig analyses data of quite different nature to the two-group models above and requires the additional information of the cancer cell fraction (CCF) for each mutation. The datasets we have simulated (C4A-C4D) are datasets where we have taken mutation data from the PCAWG cohort including their CCFs.

Selection of patient-specific changepoints The first step of the simulation is shared in all four C4 simulations: a single changepoint (i.e. the quantile of mutations sorted ascendingly by CCF where signature exposures changes) is drawn from a Dirichlet distribution centered at 0.6 (i.e. with 60% of mutations in the clonal group and 40% in the subclonal) and with a precision parameter that indicates the dispersion of these CCF changepoints values across simulated samples. As this precision parameter increases, the changepoints CCF of all samples become closer. For each patient, the observed CCFs are split into two groups, corresponding to CCFs before and after the changepoint.

Selecting of signatures and trinucleotide generation As a second step, a subset of samples and signatures from the original Lung-SCC cohort are selected. In C4A all mutations and signatures from the Lung-SCC cohort have been taken, in C4B only the first two signatures, in C4C only the first two signatures and 2000 samples taken at random, and in C4D only the first four signatures and 2000 samples taken at random. In the third step, trinucleotide mutations are drawn for each sample independently. In the case of C4A, this is done using the trinucleotide exposures observed in clonal and subclonal mutations. For the first group (before changepoint), trinucleotide mutations are drawn from a multinomial with a parameter that corresponds to the relative abundance of each trinucleotide in the cohort clonal group. For the second group (after the changepoint), the drawn trinucleotides come from a mixture of two multinomials: one with a parameter that corresponds to the relative abundance of each trinucleotide in the cohort clonal group (as before) and another with a parameter that corresponds to the relative abundance of each trinucleotide in the cohort subclonal group. As with simulations C1-4, the mixture of the two populations is determined by a parameter π. When $\pi = 0$, there is no differential abundance between groups before and after the changepoint. As π increases, differential abundance becomes more evident. In the case of C4B, C4C and C4D, the trinucleotides are drawn using the observed exposures in the clonal and subclonal group as a mixture of multinomials where each element in the mixture corresponds to a signature and the multinomial parameter is the signature definition. As before, mutations coming from clonal and subclonal exposures are mixed with a mixing parameter π.

Data preparation The fourth and last step of the simulation is again shared among all simulations in C4. The data so far is a list of mutations, each with a CCF and with a label (before or after the changepoint). For `diagREDM`, the data are split into the two groups of before and after the changepoint, and signatures are re-extracted for all patients together. For `Mreg`, mutations are binned in bins of length 100. From a large matrix containing all bins for all patients, signatures are re-extracted. In the design matrix we save the information about the mean CCF for each of the observations (bin and patient). Note that the number of bins is different for each patient owing to the different number of mutations. For TrackSig, a vcf-looking file with the information about each trinucleotide change and the CCF is saved to be used as input.

Comparison to TrackSig We find the TrackSig (run in two modalities) overestimates the changepoints, i.e. in non-differential abundance datasets it detects changepoints (Fig S43b), especially in C4A, C4B and C4D. Moreover, even though only one changepoint is included in the simulation, TrackSig invariably finds many. As TrackSig returns sample-specific data but we are interested in a cohort aggregation, we have summarised the data in the form of percentage of samples with some number of changepoints or more. Varying these two numbers does not lead to satisfactory TrackSig results in terms of weighted accuracy, as the high false positive rate is still apparent (Fig S43). Two CompSign models have been considered. The first one is `diagREDM`, the input data of which corresponds to the re-extracted exposures of the two groups of mutations before and after the changepoint (i.e. ground truth groups). The second CompSign model is `fullREM` in a regression setting (called `Mreg` in the figure), a multinomial regression model with correlated patient-specific intercepts. It is used instead of a Dirichlet-multinomial model for simplicity. For this model the input data are mutations grouped in bins of 100 mutations from which signatures are re-extracted. The covariates used are the mean CCF for each of the observations (bin and patient), and a baseline shared across bins and patients. Both `diagREDM` and `Mreg` show high weighted accuracies.

In the Fig S43a we show more in detail the results of C4C, where TrackSig performs best, but not better than the CompSign models. As π increases (differential abundance becomes more evident), all models find increasing fractions of differential abundance runs, but both TrackSig models have a high false positive rate (at $\pi = 0$), which are lower in `diagREDM` and `Mreg` (`fullREDM`). In these models, the sensitivity reaches 1 when $\pi = 0.1$, but for TrackSig it never does.
